# Projected Effects of Changing Global Tuberculosis Epidemiology on *Mycobacterium tuberculosis* Immunoreactivity Prevalence, 2024–2050

**DOI:** 10.3201/eid3203.251340

**Published:** 2026-03

**Authors:** Michelle Machado, Aria Ed Jordan, Alvaro Schwalb, Rein M.G.J. Houben, Peter J. Dodd, Katie Dale, Kevin Schwartzman, Jonathon R. Campbell

**Affiliations:** McGill University, Montreal, Quebec, Canada (M. Machado, K. Schwartzman, J.R. Campbell); University of Minnesota Foundation, Minneapolis, Minnesota, USA (A.E. Jordan); Instituto de Medicina Tropical Alexander von Humboldt, Universidad Peruana Cayetano Heredia, Lima, Peru (A. Schwalb); TB Modelling Group, TB Centre, London School of Hygiene and Tropical Medicine, London, UK (A. Schwalb, R.M.G.J. Houben); Sheffield Centre for Health and Related Research, School of Medicine and Population Health, University of Sheffield, Sheffield, UK (P.J. Dodd); Victorian Tuberculosis Program, Royal Melbourne Hospital, at the Peter Doherty Institute for Infection and Immunity, Melbourne, Victoria, Australia (K. Dale); The University of Melbourne, at the Peter Doherty Institute for Infection and Immunity, Melbourne (K. Dale); McGill International TB Centre, Montreal (K. Schwartzman, J.R. Campbell); Centre for Outcomes Research and Evaluation, Research Institute of the McGill University Health Centre, Montreal (K. Schwartzman, J.R. Campbell)

**Keywords:** tuberculosis and other mycobacteria, respiratory infections, bacteria, infectious disease, prevention, TB, modeling, public health, global health

## Abstract

We assessed how evolving global tuberculosis (TB) trends might influence *Mycobacterium tuberculosis* immunoreactivity and TB risk among persons immigrating to low-incidence countries. We projected annual risk for infection (ARI) in 168 countries for 2024–2050, focusing on China, India, the Philippines, and Vietnam. We applied projections to the age profile of immigrants to 4 low-incidence countries to estimate changes in *M. tuberculosis* immunoreactivity prevalence and TB risk under status quo and accelerated ARI decline scenarios. In the status quo 2024 estimate, *M. tuberculosis* immunoreactivity prevalence ranged from 14.7% in China to 40.1% in the Philippines, declining to 5.8% in China and 23.0% in the Philippines by 2050; TB risk also declined. Accelerated ARI reductions yielded greater relative decreases in disease risk than immunoreactivity prevalence. Declining global TB incidence could reduce *M. tuberculosis* immunoreactivity and disease risk among immigrant populations, which could inform cost–benefit analyses for future TB screening strategies in low-incidence settings.

Tuberculosis (TB) remains a major global public health challenge. In 2024, an estimated 10.7 million persons had TB develop, and ≈1.23 million persons died (*1*). TB, caused by *Mycobacterium tuberculosis*, begins as an asymptomatic infection but can progress to infectious disease at any time (*2*). Among persons with evidence of *M. tuberculosis* infection, risk of developing TB is highest in the first 2 years after infection (*3*). Persons with evidence of *M. tuberculosis* infection go through a short high-risk period, followed by a prolonged low-risk period (*1**–**3*).

*M. tuberculosis* infection is diagnosed by absence of clinical findings indicating TB disease, plus immunoreactivity to *M. tuberculosis* antigens measured by skin tests or interferon-γ release assays (*4*,*5*). Immunoreactivity can exhibit reversion (*6*) but is assumed to be lifelong for many infected persons. Immunoreactivity tests cannot distinguish persons at high risk for TB progression (i.e., recently infected [<2 years]) from those at low risk (i.e., remotely infected [>2 years]). The number of recently infected versus remotely infected persons will vary by local epidemiology, driven by the force of *M. tuberculosis* transmission.

In many low-incidence countries, TB disproportionately affects immigrant populations, largely because of progression of *M. tuberculosis* infection acquired in higher transmission settings before immigration. For that reason, several countries have implemented or considered large-scale *M. tuberculosis* infection screening and treatment programs among new immigrants (*7*,*8*). Critical to the cost-effectiveness of those programs are *M. tuberculosis* immunoreactivity prevalence and the likelihood of subsequent TB disease (*9*). TB risk is directly correlated with the percentage of recently infected persons; thus, understanding how changing TB epidemiology affects immunoreactivity prevalence and percentages of persons recently or remotely infected can have implications for cost-effectiveness and efficiency of TB programs.

In this study, we aimed to gain insights into how changing TB epidemiology would affect the *M. tuberculosis* immunoreactivity prevalence and risk for developing TB disease among new immigrants to low-incidence countries. Using the age distribution of new immigrants to 4 immigrant-receiving low-incidence countries, we estimated temporal changes in the overall *M. tuberculosis* immunoreactivity prevalence and risk for progression to TB disease in the year of arrival among immigrants from 168 countries. We focused on immigrants from 4 moderate- to high-incidence countries, India, China, the Philippines, and Vietnam, and modeled how further changes in *M. tuberculosis* transmission could affect those outcomes for 2024–2050.

## Methods

### Countries Evaluated

For this analysis, we considered persons immigrating from 168 countries (Appendix Table 1). Of those, we selected 4 moderate- to high-incidence countries (annual TB incidence ≥50/100,000 population) to examine more closely: China, India, the Philippines, and Vietnam. We selected those countries because all 4 are common countries of origin for immigrants moving to 4 common immigrant-receiving countries. In 2023, those 4 countries combined accounted for 54% of new immigrants to Canada, 19% to the United States, 12% to the United Kingdom, and 47% to Australia (*10*–*13*).

### Annual Risk for *M. tuberculosis* Infection Measured by Immunoreactivity

The diagnosis of *M. tuberculosis* infection is based on immunoreactivity to skin tests or interferon-γ release assays and cannot be empirically confirmed; thus, we focused on immunoreactivity. We estimated the annual risk for infection (ARI), which is the probability that in a given year someone will become *M. tuberculosis* immunoreactive, by leveraging methods and estimates generated for a previously published analysis (*14*). Estimates included 200 different ARI projections from 1889–2021. When available, we used historical tuberculin skin test surveys as the basis for ARI; in all other instances, we estimated ARI by using a modified Styblo rule, which relates the prevalence of smear-positive TB disease to the ARI. In both methods, ARI is related to measured immunoreactivity prevalence, and immunoreactivity is assumed to be lifelong. To estimate ARI beyond 2021, we estimated the average annual change in ARI during 2000–2021 and projected the same annual change from 2022 to 2050 for each country and ARI projection.

### Estimation of *M. tuberculosis* Immunoreactivity Prevalence

To estimate *M. tuberculosis* immunoreactivity prevalence, we adapted methods from a previously published study modeling immunoreactivity prevalence among foreign-born residents of Canada (*14*). In brief, we calculated the cumulative probability of *M. tuberculosis* immunoreactivity by integrating the ARI over time stratified by country of origin, year of birth, and year of migration. We applied annual ARI values sequentially up to each time point, assuming independent infection risk from year to year.

For each country, we used the 200 ARI trajectories estimated for 2024 to 2050 to generate 200 corresponding estimates of immunoreactivity prevalence for each year of migration and year of birth. We then applied those estimates to the age distribution of new immigrants from 4 immigrant-receiving low-incidence countries, to attain estimates of overall immunoreactivity prevalence for each year. Similarly, we used those same ARI trajectories to estimate the prevalence of recently acquired *M. tuberculosis* immunoreactivity (i.e., persons who became immunoreactive <2 years), using the ARI over a 2-year period. We selected a 2-year period for recent immunoreactivity because that timeframe corresponds to the highest risk period for progression to TB (*3*,*5*,*15*).

### Projections of *M. tuberculosis* Immunoreactivity Prevalence and TB Risk

To estimate the age distribution of immigrants, we analyzed immigration data from Canada, the United States, the United Kingdom, and Australia, which detail the demographic characteristics of new immigrants each year (Appendix). We categorized those values into 6 age ranges: 0–14, 15–29, 30–44, 45–59, 60–74, and >75 years (*16*). We assumed the age distribution was the same for each country of origin and that specific ages were equally distributed within each range. 

To project the age distribution into the future, we calculated the mean percentage and SD of new immigrants belonging to each age group in the most recent year from which data were available (2023–2024) across countries by using equal weighting and assumed that distribution remained stable from 2024 to 2050. We fit each age category to a β distribution (Appendix Table 2), and generated 200 sets of age distribution estimates, scaling values to ensure each set summed to 100%. Therefore, we estimated that, of all new immigrants, 16.4% (95% uncertainty interval [UI] 10.3%–24.9%) would be 0–14 years of age, 25.2% (95% UI 14.4%–36.8%) would be 15–29 years of age, 34.5% (95% UI 24.4%–47.0%) would be 30–44 years of age, 15.3% (95% UI 8.5%–24.8%) would be 45–59 years of age, 6.3% (95% UI 2.7%–11.4%) would be 60–74 years of age, and 1% (95% UI 0.3%–2.5%) would be ≥75 years of age at the time of immigration.

For each year during 2024–2050, we used each of the 200 ARI trajectories to estimate the overall *M. tuberculosis* immunoreactivity prevalence among persons immigrating from each country, as well as the acquired immunoreactivity prevalence in the previous 2 years under a status quo scenario (i.e., no change in projected ARI trajectories). Using the projections of overall and recent *M. tuberculosis* immunoreactivity, we estimated the average annual TB incidence in the year of arrival among new immigrants overall from each country, as well as the TB incidence in the year of arrival restricted to those with underlying *M. tuberculosis* immunoreactivity. 

To estimate risk for TB progression, we assumed that the annual risk for TB progression for persons who recently acquired immunoreactivity was similar to that estimated in a large systematic review of TB risk among contacts (*17*). For persons with remotely (>2 years before immigration) acquired immunoreactivity, we based estimates on observed TB incidence data in Canada (Appendix). We used those data sources to fit risks to lognormal distributions (Appendix) and generated 200 estimates, which resulted in estimates of TB risk in the year of arrival at 1.7% (95% UI 1.3%–2.2%) among persons with recent *M. tuberculosis* immunoreactivity and at 0.07% (95% UI 0.06%–0.09%) among persons with remote *M. tuberculosis* immunoreactivity.

To assess how reducing *M. tuberculosis* transmission would affect each of those outcomes, we modeled 3 different scenarios in which we reduced the ARI trajectories in our status quo projections beginning in 2025 to an absolute 1% additional reduction, 3% additional reduction, and 5% additional reduction in ARI. For example, if the status quo scenario had an estimated annual relative ARI decrease of 3% for a given country, we modeled scenarios in which the relative ARI decrease was 4%, 6%, or 8% annually. We calculated absolute and relative reductions by comparing each reduction scenario to the status quo. We stratified data by country and by age groups to identify trends and differences in estimates among strata.

We conducted a sensitivity analysis in which we loosened our assumption that *M. tuberculosis* immunoreactivity was lifelong, allowing for reversion (*6*,*18*). Because our ARI estimates are based on measured immunoreactivity, if reversion occurred, we would estimate the same level of overall measured immunoreactivity prevalence, but ARI would necessarily need to be higher to achieve that prevalence. Using those higher ARIs, recently acquired immunoreactivity prevalence would increase. Therefore, to maintain observed levels of immunoreactivity in our sensitivity analysis, we estimated the attendant increase in the ARI assuming an annual reversion rate of 10%, per previously described data (*18*,*19*). Thus, considering reversion was equivalent to a 2.9-fold increase in ARI, and we used that assumption to reestimate the prevalence of recently acquired immunoreactivity and the average annual risk for TB progression for the years 2024–2050.

We reported estimates as medians and 95% UIs (2.5th and 97.5th percentiles) across the 200 ARI trajectories. We compared outputs of *M. tuberculosis* immunoreactivity prevalence and TB disease risk to previous estimates for face validity (Appendix). We conducted all analyses in R version 4.3.1 (The R Project for Statistical Computing, https://www.r-project.org). We performed preliminary data organization and cleaning using Excel 2024 (Microsoft, https://www.microsoft.com). We created graphic representations using the ggplot2 package version 3.5.1 (https://github.com/cran/ggplot2) and GraphPad (GraphPad Software Inc., https://www.graphpad.com). This study used publicly available deidentified, aggregate data, and did not include personal identifiers in any data analyzed; thus, approval from a research ethics board was not required.

## Results

### Estimated Changes in ARI

We estimated the Philippines would have the highest 2024 ARI at 0.98% (95% UI 0.46%–2.2%), followed by India at 0.48% (95% UI 0.24%–0.95%), Vietnam at 0.41% (95% UI 0.13%–1.20%), and China at 0.19% (95% UI 0.08%–0.47%) (Table 1; Appendix Figure 1). Across all 4 countries, the estimated ARI generally declined during 2000–2021, although in all countries except India, a small number of trajectories projected increases. Specifically, we estimated the ARI would fall at a relative rate of 3.8% per year (95% UI 7.3% decline to 0.2% increase) in China, 4.1% per year (95% UI 7.1% decline to 0.2% decline) in India, 3.1% per year (95% UI 7.1% decline to 0.7% increase) in the Philippines, and 3.8% per year (95% UI 8.8% decline to 1.6% increase) in Vietnam. Following those projections, compared with 2024, the 2050 ARI was 61.8% lower (95% UI 84.7% lower to 5.0% higher) in China, 64.7% lower (95% UI 84.0% lower to 5.5% higher) in India, 53.8% lower (95% UI 83.9% lower to 19.5% higher) in the Philippines, and 61.6% lower (95% UI 89.7% lower to 46.8% higher) in Vietnam. We also calculated ARI trends for all 168 countries (Appendix Table 3).

**Table 1:** Projected annual risk for infection in a study of global tuberculosis epidemiology on *Mycobacterium tuberculosis* immunoreactivity prevalence, 2024–2050*

Country	Status quo ARI, % (95% UI)			2050 ARI, % (95% UI), under additional ARI reduction scenarios		
	2024	2050		Additional 1% reduction	Additional 3% reduction	Additional 5% reduction
China	0.19 (0.08–0.47)	0.07 (0.01–0.47)		0.05 (0.01–0.35)	0.03 (0.01–0.19)	0.02 (0–0.11)
India	0.48 (0.24–0.95)	0.17 (0.04–0.98)		0.13 (0.03–0.74)	0.07 (0.01–0.41)	0.04 (0.01–0.22)
Philippines	0.98 (0.46–2.2)	0.44 (0.08–2.7)		0.32 (0.06–2.0)	0.18 (0.03–1.1)	0.09 (0.02–0.62)
Vietnam	0.41 (0.13–1.2)	0.16 (0.01–1.7)		0.12 (0.01–1.3)	0.06 (0.01–0.70)	0.03 (0–0.38)

*Analysis of percentage ARI under the status quo scenario and 3 scenarios of additional ARI reduction of 1%, 3%, and 5%. ARI, annual risk for infection; UI, uncertainty interval.

### Projected Changes in Immunoreactivity Prevalence

**Figure 1:**
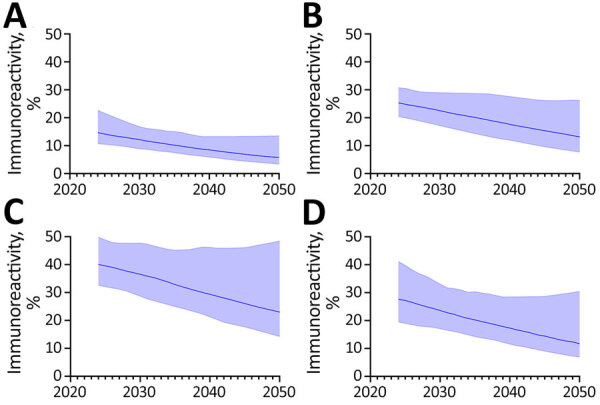
Projected immunoreactivity in study of effects of global tuberculosis epidemiology on *Mycobacterium tuberculosis* immunoreactivity prevalence, 2024–2050. Graphs show projected prevalence of *M. tuberculosis* immunoreactivity among immigrants from China (A), India (B), the Philippines (C), and Vietnam (D) under the status quo scenario, i.e., assuming continuation of projected annual risk for infection trends without additional reduction. Solid lines represent median estimates, and shaded areas indicate 95% uncertainty intervals.

In general, declining ARI trends translated into reductions in the point estimates of immunoreactivity prevalence among immigrant populations from China, India, the Philippines, and Vietnam. In 2024, we estimated prevalence among new immigrants from each country to be 14.7% (95% UI 10.7%–22.7%) for China, 25.4% (95% UI 20.4%–30.8%) for India, 40.1% (95% UI 32.6%–49.8%) for the Philippines, and 27.7% (95% UI 19.5%–41.2%) for Vietnam (Figure 1). By 2050, we projected prevalence would decrease to 5.8% (95% UI 3.4%–13.5%) for China, 13.2% (95% UI 7.7%–26.2%) for India, 23.0% (95% UI 14.3%–48.5%) for the Philippines, and 11.7% (95% UI 6.9%–30.5%) for Vietnam (Appendix Tables 4, 5). 

The prevalence of recent immunoreactivity remained relatively stable over time in the status quo scenario. By 2050, we projected the prevalence of recently acquired immunoreactivity would be 0.14% (95% UI 0.03%–0.78%) for China, 0.30% (95% UI 0.07%–1.40%) for India, 0.67% (95% UI 0.14%–2.6%) for the Philippines, and 0.28% (95% UI 0.03%–2.1%) for Vietnam (Appendix Table 6). In our reversion sensitivity analysis, corresponding estimates in 2050 were 0.4% (95% UI 0.08%–2.3%) for China, 0.86% (95% UI 0.21%–4.1%) for India, 1.9% (95% UI 0.41%–7.4%) for the Philippines, and 0.80% (95% UI 0.08%–6.0%) for Vietnam (Appendix Table 6).

**Figure 2:**
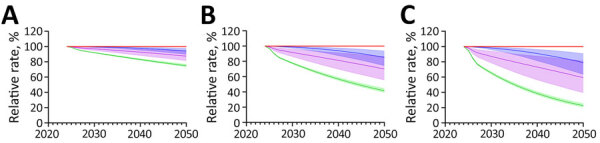
Projected effects on tuberculosis (TB) incidence and *Mycobacterium tuberculosis* immunoreactivity in study of effects of global tuberculosis epidemiology on *M. tuberculosis* immunoreactivity prevalence, 2024–2050. Graphs show effects over time among immigrants from China under 3 scenarios for reduction in annual risk for infection: A) 1% additional reduction; B) 3% additional reduction; C) 5% additional reduction. Solid lines represent median estimates; shaded areas indicate 95% uncertainty intervals. Red line indicates status quo scenario (i.e., no change in percent immunoreactivity or TB incidence); blue indicates overall *M. tuberculosis* immunoreactivity; green indicates recent (<2 years) *M. tuberculosis* immunoreactivity; and purple indicates TB disease risk in the year of immigration to low-incidence country.

**Figure 3:**
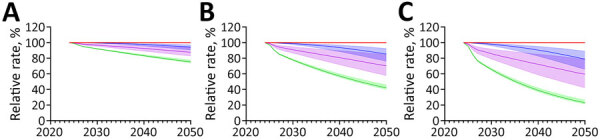
Projected effects on tuberculosis (TB) incidence and *Mycobacterium tuberculosis* immunoreactivity in study of effects of global tuberculosis epidemiology on *M. tuberculosis* immunoreactivity prevalence, 2024–2050. Graphs show effects over time among immigrants from India under 3 scenarios for reduction in annual risk for infection: A) 1% additional reduction; B) 3% additional reduction; C) 5% additional reduction. Solid lines represent median estimates; shaded areas indicate 95% uncertainty intervals. Red line indicates status quo scenario (i.e., no change in percent immunoreactivity or TB incidence); blue indicates overall *M. tuberculosis* immunoreactivity; green indicates recent (<2 years) *M. tuberculosis* immunoreactivity; and purple indicates TB disease risk in the year of immigration to low-incidence country.

**Figure 4:**
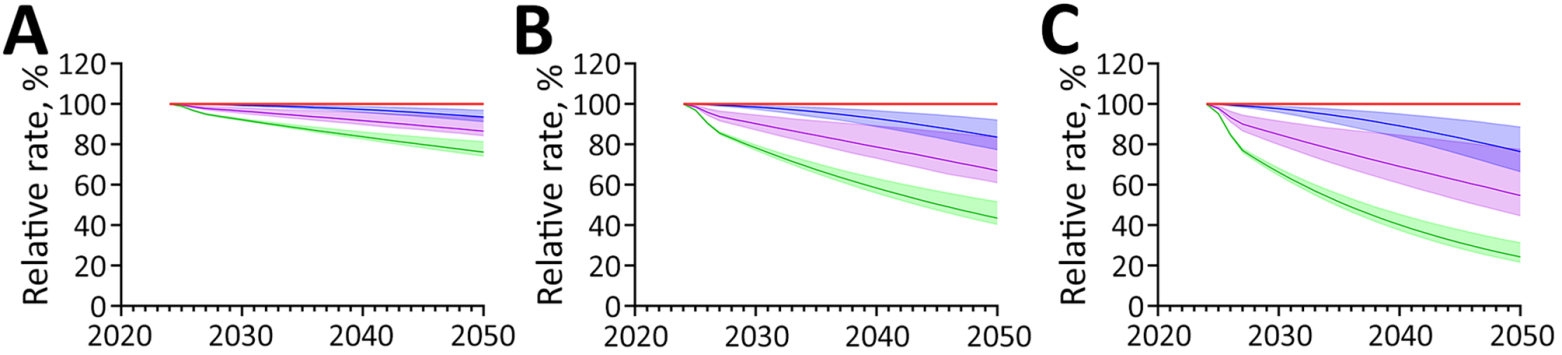
Projected effects on tuberculosis (TB) incidence and *Mycobacterium tuberculosis* immunoreactivity in study of effects of global tuberculosis epidemiology on *M. tuberculosis* immunoreactivity prevalence, 2024–2050. Graphs show effects over time among immigrants from the Philippines under 3 scenarios for reduction in annual risk for infection: A) 1% additional reduction; B) 3% additional reduction; C) 5% additional reduction. Solid lines represent median estimates; shaded areas indicate 95% uncertainty intervals. Red line indicates status quo scenario (i.e., no change in percent immunoreactivity or TB incidence); blue indicates overall *M. tuberculosis* immunoreactivity; green indicates recent (<2 years) *M. tuberculosis* immunoreactivity; and purple indicates TB disease risk in the year of immigration to low-incidence country.

**Figure 5:**
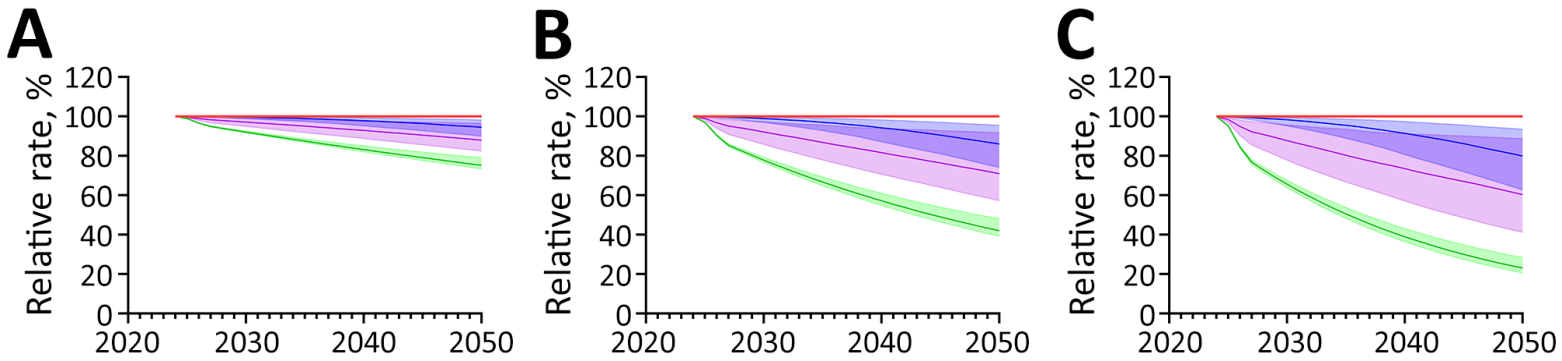
Projected effects on tuberculosis (TB) incidence and *Mycobacterium tuberculosis* immunoreactivity in study of effects of global tuberculosis epidemiology on *M. tuberculosis* immunoreactivity prevalence, 2024–2050. Graphs show effects over time among immigrants from Vietnam under 3 scenarios for additional reduction in annual risk for infection: A) 1% additional reduction; B) 3% additional reduction; C) 5% additional reduction. Solid lines represent median estimates; shaded areas indicate 95% uncertainty intervals. Red line indicates status quo scenario (i.e., no change in percent immunoreactivity or TB incidence); blue indicates overall *M. tuberculosis* immunoreactivity; green indicates recent (<2 years) *M. tuberculosis* immunoreactivity; and purple indicates TB disease risk in the year of immigration to low-incidence country.

Increasing the rate of ARI decline had modest effects on the absolute decline in immunoreactivity prevalence, regardless of the increase in the rate of decline (Appendix Tables 7, 8). We noted much greater effects on the prevalence of recent *M. tuberculosis* immunoreactivity under ARI decline scenarios (Figures 2,3,4,5). Under the additional 1% ARI decline scenario, we projected the overall immunoreactivity prevalence across China, India, the Philippines, and Vietnam would decrease by ≈6% by 2050 relative to the status quo scenario. However, recent immunoreactivity prevalence fell more sharply, declining by ≈25%. Under the additional 5% ARI decline scenario, those reductions were more pronounced; overall immunoreactivity prevalence decreased by up to 21%–23%, and recent immunoreactivity prevalence fell by 75%–77% relative to the status quo.

Age-stratified projections revealed that declines in immunoreactivity prevalence varied by age group and had a strong inverse relationship between age and reduction magnitude. Younger cohorts, having experienced fewer cumulative years of exposure, received the largest benefits from reduced ARI. For instance, by 2050 under the additional 3% ARI decline scenario, we projected the immunoreactivity in children 0–14 years of age would fall below 1% among children from China, India, and Vietnam, although it remained slightly above 1% for children from the Philippines. In contrast, older adults retained higher immunoreactivity levels due to prior exposures accumulated over their lifetimes (Appendix Table 9). The most substantial declines occurred in settings with initially high transmission, notably the Philippines and India, where the effects of accelerated ARI reductions were substantial. As expected, the percentage of recently acquired immunoreactivity was highest in the youngest age groups and increased in sensitivity analyses that incorporated reversion (Appendix Tables 10, 11).

### Projected Changes in the Average TB Risk

Under the status quo scenario, we estimated overall annual TB incidence per 100,000 new immigrants arriving in 2024 would be 16.4 (95% UI 11.0–24.9) among those from China, 29.7 (95% UI 20.1–47.4) among those from India, 47.9 (95% UI 34.5–72.0) among those from the Philippines, and 30.2 (95% UI 21.2–47.3) among those from Vietnam. We projected that by 2050 those risks among new immigrants would fall to 6.4 (95% UI 3.0–22.7) among those from China, 14.5 (95% UI 6.8–41.9) among those from India, 27.1 (95% UI 12.2–76.0) among those from the Philippines, and 12.9 (95% UI 5.9–53.9) among those from Vietnam (Table 2; Appendix Table 12). As a result of the higher estimated prevalence of recently acquired immunoreactivity, we estimated TB incidence in the arrival year would be nearly double in sensitivity analyses incorporating immunoreactivity reversion (Table 2; Appendix Tables 13, 14).

**Table 2:** Projected annual tuberculosis disease incidence among new immigrants in a study of effects of global tuberculosis epidemiology on *Mycobacterium tuberculosis* prevalence and immunoreactivity, 2024–2050*

Country	Status quo incidence (95% UI)			2050 incidence (95% UI) under additional ARI reduction scenarios		
	2024	2050		Additional 1% reduction	Additional 3% reduction	Additional 5% reduction
Primary analysis						
China	16.4 (11.0–24.9)	6.4 (3.0–22.7)		5.5 (2.7–18.6)	4.4 (2.3–12.7)	3.8 (2.1–9.1)
India	29.7 (20.1–47.4)	14.5 (6.8–41.9)		12.7 (6.3–34.7)	10.1 (5.4–24.2)	8.5 (4.9–18.3)
Philippines	47.9 (34.5–72.0)	27.1 (12.2–76.0)		23.5 (11.0–65.6)	18.5 (9.0–48.7)	15.2 (8.1–36.7)
Vietnam	30.2 (21.2–47.3)	12.9 (5.9–53.9)		11.1 (5.5–45.5)	9.1 (5.0–32.8)	7.9 (4.6–24.4)
Sensitivity analysis†						
China	26.0 (16.9–50.2)	10.6 (3.7–48.7)		8.8 (3.4–38.5)	6.2 (2.9–24.1)	4.7 (2.4–15.6)
India	51.1 (30.9–96.4)	23.5 (9.2–86.9)		19.5 (8.1–70.1)	14.2 (6.6–45.8)	10.8 (5.5–30.5)
Philippines	83.7 (51.5–139.5)	48.5 (15.6–149.4)		39.4 (14.0–122.4)	27.5 (11.8–86.5)	20.2 (9.4–61.2)
Vietnam	46.9 (29.4–92.1)	20.8 (7.2–113.0)		17.4 (6.6–92.3)	12.6 (5.6–61.2)	9.6 (5.0–38.6)

*Annual incidence per 100,000 persons under the status quo scenario and 3 scenarios of additional ARI reduction of 1%, 3%, and 5%. ARI, annual risk for infection; UI, uncertainty interval. †Assuming increased risk for recently acquired *M. tuberculosis* immunoreactivity.

Accelerating declines in ARI led to further reductions in TB risk, largely driven by reductions in the prevalence of recently acquired *M. tuberculosis* immunoreactivity (Appendix Figure 2). Under the additional 3% ARI decline scenario, we estimated the annual risk per 100,000 new immigrants arriving in 2050 would be 4.4 (95% UI 2.3–12.7) among those from China, 10.1 (95% UI 5.4–24.2) among those from India, 18.5 (95% UI 9.0–48.7) among those from the Philippines, and 9.1 (95% UI 5.0–32.8) among those from Vietnam (Table 2). As with immunoreactivity prevalence, we noted greater declines in TB risk in younger age categories (Appendix Tables 12, 13). When we estimated TB risk in arrival year only among those with *M. tuberculosis* immunoreactivity, TB risk fell by ≈7% in the additional 1% ARI decline scenario, 18% in the 3% scenario, and 25% in the 5% scenario relative to the status quo (Appendix Table 15).

## Discussion

In this study, we found that among 4 countries with moderate to high TB incidence, China, India, the Philippines, and Vietnam, recent trends suggest ARI is falling 2%–3% per year. Accordingly, we projected *M. tuberculosis* immunoreactivity prevalence, a proxy for *M. tuberculosis* infection, and average TB risk would decrease among immigrants from those countries by 2050, and the Philippines would have the greatest absolute declines. Accelerating ARI declines, even by modest amounts, had larger relative effects on TB risks compared with *M. tuberculosis* immunoreactivity prevalence, primarily driven by declines in recently acquired immunoreactivity.

The cost-effectiveness of immigration *M. tuberculosis* infection screening and treatment programs is an ongoing area of debate. Some analyses have found such programs to be highly cost-prohibitive (*20*–*22*), but others have found them to be cost-effective compared with traditionally accepted willingness-to-pay thresholds (*23*,*24*). However, the major drivers of the cost effectiveness of any *M. tuberculosis* infection screening program are the underlying prevalence of *M. tuberculosis* immunoreactivity and risk of developing TB. Our analysis projects the cost effectiveness of those programs to generally worsen over time as the immunoreactivity prevalence and risk of developing TB decline. For instance, our estimates showed that, among new immigrants from the Philippines, immunoreactivity prevalence would drop from 40.1% in 2024 to 23.0% in 2050 and TB risk in the year of arrival would fall from 47.9/100,000 immigrants in 2024 to 27.1/100,000 immigrants in 2050. However, those effects are unlikely to be homogenous within and between countries. That finding highlights the need for constant evaluation of TB screening programs where they are implemented (*25*) to ensure efficient use of healthcare funds.

A 2024 modeling study highlighted the potential economic and health benefits of improvements in global TB prevention and care in low-incidence settings (*26*). In line with those findings, we found substantial reductions in the population-level TB risk in scenarios with accelerated ARI declines. We also found that in those scenarios, TB risk declined more rapidly than overall *M. tuberculosis* immunoreactivity prevalence. That finding suggests that, in addition to the overall decline in population-level TB risk, the individual-level risk–benefit calculus of providing TB preventive treatment could change. Therefore, if declines in global TB incidence accelerate in the future, programs would need to anticipate and adapt to those changes, such as by using more targeted approaches to TB screening and treatment.

We found consideration of *M. tuberculosis* immunoreactivity reversion increased estimates of TB disease risks in the year of arrival for new immigrants, driven by higher ARIs and therefore increased prevalence of recently acquired *M. tuberculosis* immunoreactivity. That aspect has been identified by others (*27*). However, cohort data have shown TB risk persists, albeit reduced, among persons who had immunoreactivity reversion, and that reversion might not be stable (i.e., persons reconvert to immunopositive) (*6*). The extent of stable immunoreactivity reversion thus has implications on the value of prompt immunoreactivity testing and treatment after immigration (*28*), when risk for recent exposure would be highest.

Key strengths of this study are the consideration of multiple policy-relevant outcomes, use of previously reported data and methodology to perform the analyses, and consistency of our estimates with existing empiric literature. In addition, we included a breadth of countries and focused on countries comprising a large number of new immigrants to low-incidence countries. We also used uncertainty in several key underlying parameters and explored the effects of underlying assumptions around immunoreactivity reversion.

The first limitation of our study is that our analysis equated *M. tuberculosis* immunoreactivity to infection. Because current technologies to identify *M. tuberculosis* infection rely on immunoreactivity and data underlying TB progression rates reflect observations based on immunoreactivity, that was a reasonable approach. However, our main analysis assumed *M. tuberculosis* immunoreactivity would be lifelong, which might underestimate prevalence of recently acquired immunoreactivity. Therefore, we evaluated the effect of that assumption in a sensitivity analysis. When we incorporated immunoreactivity reversion, which implies a higher estimated prevalence of recently acquired immunoreactivity, the estimated TB risks increased. Second, we made several simplifying assumptions to focus the analysis on the potential effect of changing infection risks. We projected ARI trends beyond 2021 on the basis of trends from 2000–2021 and used a similar age distribution for immigrants from all countries. Those assumptions might not hold true for all countries if immigration patterns change or if global TB funding is interrupted (*1*,*29*). Third, we did not consider protection from reinfection in our model (*30*), which might lead to slight overestimation of the percentage of persons with recent infection. Fourth, we assumed no heterogeneity in risk for *M. tuberculosis* immunoreactivity or TB progression by demographic, clinical, or other factors among immigrants from each country. We did not explicitly model specific high-risk groups (e.g., persons with HIV) within immigrant cohorts and did not consider immigrants to differ systematically from nonimmigrants within any given country when considering infection or TB progression.

In summary, we found changing global TB epidemiology could lead to reduced *M. tuberculosis* immunoreactivity and TB risk among immigrants to low-incidence countries. Such changes might worsen the cost-effectiveness of *M. tuberculosis* infection screening and treatment programs, requiring further targeting of these programs over time, as well as alter the individual-level balance of risks and benefits of TB preventive treatment.

Appendix Additional information on projected effects of global tuberculosis epidemiology on *Mycobacterium tuberculosis* prevalence and immunoreactivity, 2024–2050.
