# Supplementary material for: Projected Effects of Changing Global Tuberculosis Epidemiology on Mycobacterium tuberculosis Immunoreactivity Prevalence, 2024–2050
Source: Emerg Infect Dis. 2026 Mar;32(3):332–40. doi: 10.3201/eid3203.251340 (PMC13016019; doi:10.3201/eid3203.251340)
Supplement: Appendix — Additional information on projected effects of global tuberculosis epidemiology on Mycobacterium tuberculosis prevalence and immunoreactivity, 2024–2050. [file 25-1340-Techapp-s1.pdf]

Article DOI: https://doi.org/10.3201/eid3203.251340

EID cannot ensure accessibility for supplementary materials supplied by authors. Readers who have difficulty accessing supplementary content should contact the authors for assistance.

Projected Effects of Changing Global Tuberculosis Epidemiology on *Mycobacterium tuberculosis* Prevalence and Immunoreactivity, 2024–2050

Appendix

Additional methods for estimating uncertainty in key parameters and evaluating face validity

Calculation of the proportion of new immigrants within each age stratum

To generate estimates of the age of new immigrants, we used the age profile of new permanent residents (or equivalent) from Canada (https://open.canada.ca/data/en/dataset/f7e5498e-0ad8-4417-85c9-9b8aff9b9eda/resource/d1c1f4f3-2d7f-4e02-9a79-7af98209c2f3), the USA (https://ohss.dhs.gov/topics/immigration/lawful-permanent-residents/profiles), the United Kingdom (https://www.gov.uk/government/statistical-data-sets/immigration-system-statistics-data-tables#citizenship), and Australia (https://dev.magda.io/dataset/ds-dga-e87976fd-c545-4ec0-ab5b-034080868624/distribution/dist-dga-1a627f37-7f5c-4068-aa4f-e6deafd60e2f/details?q =). We then used these data to estimate a β distribution based on the range of values presented. These are detailed in Appendix Table 2.

Calculation of tuberculosis disease risk among those with recently acquired immunoreactivity

We leveraged a large systematic review estimating the absolute risk of tuberculosis disease among recent tuberculosis contacts with immunoreactivity based on a positive interferon-gamma release assay to estimate tuberculosis disease risk among those with recently acquired. This value was 1.7 (95% CI: 1.3 to 2.2) per 100 person years. We fit this estimate to a log-

normal distribution [LogNormal(0.53,0.137)] and used this to generate 200 estimates of tuberculosis disease risk among those with recently acquired immunoreactivity.

Calculation of tuberculosis disease risk among those with remotely acquired immunoreactivity

We used tuberculosis incidence data among the foreign-born population in Canada (Tuberculosis in Canada, 2023; https://www.canada.ca/en/public-health/services/publications/diseases-conditions/tuberculosis-disease-2023-infographic.html), coupled with estimates of overall prevalence of tuberculosis infection (Jordan et al. CMAJ, 2023). In 2023, the tuberculosis incidence rate among the foreign-born population in Canada was 15.5 per 100,000 population. Tuberculosis immunoreactivity prevalence in 2021 was estimated to be 22% (95% UI: 19%–27%) among foreign-born Canadians—being relatively stable since 2001. We assumed the prevalence in 2021 would be the same in 2023. We fit the TB infection prevalence estimate to a β distribution [Beta(62.2,220.6)] and used this to generate 200 estimates of tuberculosis disease risk among those with remotely acquired immunoreactivity. We used the following:

Annual incidence of TB disease / Proportion with immunoreactivity = (15.5/100000)/TB infection prevalence = 0.07% (95%UR: 0.058 to 0.089) annual risk.

Comparison of Mtb immunoreactivity prevalence and tuberculosis disease risk for face validity

To examine if our estimates of Mtb immunoreactivity prevalence and tuberculosis disease risk were reasonable and consistent with existing literature, we compared estimates either generated in our previous analysis using these data or within this analysis, with comparable cohorts where Mtb infection testing was performed or tuberculosis disease incidence was observed.

The model underlying the current analysis was previously used to estimate Mtb immunoreactivity prevalence in Canada (Jordan et al. CMAJ, 2023), arriving at an estimate of 22% (95% UI: 19%–27%). There are no representative Mtb infection surveys in Canada to compare this estimate to, however Mtb immunoreactivity prevalence is likely similar in other low-incidence, immigrant-receiving countries. Thus, we compared this estimate to surveys done in the USA, United Kingdom, and Netherlands among immigrant groups. These estimates were 20.5% in the USA, 20% in the UK, and 20% in the Netherlands (see: Miramontes R, et al. PLoS

One 2015;10:e0140881; Pareek M, et al. Lancet Infect Dis 2011;11:435–44; Mulder C, et al. Eur Respir J 2012;40:1443–9.).

With respect to TB disease incidence, we compared our estimated risk in the year of arrival among all 168 countries to observed incidence in British Columbia (Ronald et al, CMAJ, 2018, 190(8):E209–216). In the year of arrival in British Columbia, estimated incidence per 100,000 population was ≈40 per 100,000 (see Figure 2A of the manuscript). These estimates are for immigrants arriving to British Columbia between 1985 and 2013, with a median arrival of ≈2004 based on information provided in Table 1. During the past 15 years, global tuberculosis incidence has been falling at ≈1.5% per annum according to data provided by the WHO Global TB Report in 2025 (i.e., global incidence in 2010 estimated to be 169 per 100,000 and in 2024 to be 133 per 100,000). Applying this trend to estimated tuberculosis incidence in the year of arrival among new immigrants British Columbia according to Ronald et al, would equate to an incidence of ≈30 per 100,000 in the year 2024 (i.e., if we assume incidence would fall 1.5% per annum from 2004 to 2024). Using our modeled estimates and fit to the distribution of immigrants to Canada according to Immigrants, Refugees, and Citizenship Canada (https://open.canada.ca/data/en/dataset/f7e5498e-0ad8-4417-85c9-9b8aff9b9eda/resource/d1c1f4f3-2d7f-4e02-9a79-7af98209c2f3), we estimated the incidence to be 29 per 100,000 in the year 2024. While not a perfect comparison, it demonstrated our estimates are comparable to observed population-level tuberculosis incidence data among new immigrants to an immigrant-receiving, low-incidence country.

Appendix Table 1. ISO3 codes of countries included in the analysis

ISO3	Country
AFG	Afghanistan
AGO	Angola
ALB	Albania
ARE	United Arab Emirates
ARG	Argentina
ARM	Armenia
ATG	Antigua and Barbuda
AUS	Australia
AUT	Austria
AZE	Azerbaijan
BDI	Burundi
BEL	Belgium
BEN	Benin, Republic of
BFA	Burkina-Faso
BGD	Bangladesh
BGR	Bulgaria
BHR	Bahrain
BHS	Bahama Islands, The
BIH	Bosnia-Herzegovina

ISO3	Country
BLR	Belarus
BLZ	Belize
BOL	Bolivia
BRA	Brazil
BRB	Barbados
BRN	Brunei
BTN	Bhutan
BWA	Botswana, Republic of
CAF	Central African Republic
CHE	Switzerland
CHL	Chile
CIV	Ivory Coast, Republic of
CMR	Cameroon, Federal Republic of
COD	Congo, Democratic Republic of the
COG	Congo, Republic of the
COL	Colombia
COM	Comoros
CRI	Costa Rica
CUB	Cuba
CYP	Cyprus
CZE	Czech Republic
DEU	Germany
DJI	Djibouti, Republic of
DMA	Dominica
DNK	Denmark
DOM	Dominican Republic
DZA	Algeria
ECU	Ecuador
EGY	Egypt
ERI	Eritrea
ESP	Spain
EST	Estonia
ETH	Ethiopia
FIN	Finland
FJI	Fiji
FRA	France
GAB	Gabon Republic
GBR	United Kingdom and Overseas Territories
GEO	Georgia
GHA	Ghana
GIN	Guinea, Republic of
GMB	Gambia
GNB	Guinea-Bissau
GRC	Greece
GRD	Grenada
GTM	Guatemala
GUY	Guyana
HKG	Hong Kong Special Administrative Region, China SAR
HND	Honduras
HRV	Croatia
HTI	Haiti
HUN	Hungary
IDN	Indonesia, Republic of
IRL	Ireland, Republic of
IRN	Iran
IRQ	Iraq
ISL	Iceland
ISR	Israel
ITA	Italy
JAM	Jamaica
JOR	Jordan
JPN	Japan
KAZ	Kazakhstan
KEN	Kenya

ISO3	Country
KGZ	Kyrgyzstan
KHM	Cambodia
KNA	St. Kitts-Nevis
KOR	Korea, Republic of
KWT	Kuwait
LAO	Lao People’s Democratic Republic
LBN	Lebanon
LBR	Liberia
LBY	Libya
LCA	St. Lucia
LKA	Sri Lanka
LTU	Lithuania
LUX	Luxembourg
LVA	Latvia
MAC	Macau SAR
MAR	Morocco
MDA	Moldova
MDG	Madagascar
MEX	Mexico
MKD	Macedonia
MLI	Mali, Republic of
MLT	Malta
MMR	Myanmar (Burma)
MNE	Montenegro, Republic of
MNG	Mongolia, People's Republic of
MOZ	Mozambique
MRT	Mauritania
MUS	Mauritius
MWI	Malawi
MYS	Malaysia
NAM	Namibia
NER	Niger, Republic of the
NGA	Nigeria
NIC	Nicaragua
NLD	Netherlands, The
NOR	Norway
NPL	Nepal
NZL	New Zealand
OMN	Oman
PAK	Pakistan
PAN	Panama, Republic of
PER	Peru
POL	Poland
PRT	Portugal
PRY	Paraguay
PSE	Palestinian Authority (Gaza/West Bank)
ROU	Romania
RUS	Russia
RWA	Rwanda
SAU	Saudi Arabia
SDN	Sudan, Democratic Republic of
SEN	Senegal
SGP	Singapore
SLE	Sierra Leone
SLV	El Salvador
SOM	Somalia, Democratic Republic of
SRB	Serbia, Republic of
SSD	South Sudan, Republic of
SUR	Surinam
SVK	Slovak Republic
SVN	Slovenia
SWE	Sweden
SWZ	Swaziland
SYC	Seychelles
SYR	Syria

ISO3	Country
TCD	Chad, Republic of
TGO	Togo, Republic of
THA	Thailand
TJK	Tajikistan
TKM	Turkmenistan
TTO	Trinidad and Tobago, Republic of
TUN	Tunisia
TUR	Turkey
TZA	Tanzania, United Republic of
UGA	Uganda
UKR	Ukraine
URY	Uruguay
USA	United States of America
UZB	Uzbekistan
VCT	St. Vincent and the Grenadines
VEN	Venezuela
YEM	Yemen
ZAF	South Africa, Republic of
ZMB	Zambia
ZWE	Zimbabwe

Appendix Table 2. Data use to estimate a β distribution based on the range of values presented

Age group	Canada	USA	UK	Australia	Beta Parameters
0–14 y	0.179	0.11	0.20	0.16	(14.7,76.1)
15–29 y	0.318	0.27	0.16	0.26	(10.5,31.3)
30–44 y	0.36	0.385	0.40	0.23	(12.5,23.9)
45–59 y	0.086	0.165	0.18	0.18	(9.6,53.2)
60–74 y	0.047	0.06	0.05	0.1	(6.3,93.0)
≥75 y	0.01	0.01	0.01	0.06	(3.6,334)

*As sampled values may not sum to 1 across age groups, we reweighted all estimates after sampling to ensure the sum of the age distribution was equal to 1.

Appendix Table 3. Projected annual risk of tuberculosis infection by country and year (2024 and 2050), under the status quo scenario and three scenarios of additional annual risk of infection reduction (1%, 3% and 5%) for all other countries

ISO3	Status quo		2050 (95% UI) with additional reduction in ARI		
Code	2024 (95% UI)	2050 (95% UI)	Additional 1% reduction	Additional 3% reduction	Additional 5% reduction
AFG	0.69% (0.29% to 1.6%)	0.43% (0.06% to 2.9%)	0.32% (0.05% to 2.2%)	0.18% (0.02% to 1.2%)	0.09% (0.01% to 0.69%)
AGO	1.4% (0.54% to 3.8%)	1.5% (0.16% to 11.0%)	1.1% (0.12% to 8.3%)	0.60% (0.06% to 4.7%)	0.33% (0.03% to 2.6%)
ALB	0.05% (0.02% to 0.15%)	0.03% (0.00% to 0.38%)	0.02% (0.00% to 0.29%)	0.01% (0.00% to 0.16%)	0.01% (0.00% to 0.09%)
ARE	0.00% (0.00% to 0.01%)	0.00% (0.00% to 0.00%)	0.00% (0.00% to 0.00%)	0.00% (0.00% to 0.00%)	0.00% (0.00% to 0.00%)
ARG	0.06% (0.02% to 0.15%)	0.02% (0.00% to 0.22%)	0.02% (0.00% to 0.16%)	0.01% (0.00% to 0.09%)	0.00% (0.00% to 0.05%)
ARM	0.19% (0.05% to 0.53%)	0.17% (0.02% to 1.3%)	0.13% (0.01% to 0.99%)	0.07% (0.01% to 0.56%)	0.04% (0.00% to 0.31%)
ATG	0.02% (0.01% to 0.06%)	0.02% (0.00% to 0.27%)	0.02% (0.00% to 0.20%)	0.01% (0.00% to 0.12%)	0.00% (0.00% to 0.07%)
AUS	0.02% (0.01% to 0.07%)	0.02% (0.00% to 0.32%)	0.02% (0.00% to 0.25%)	0.01% (0.00% to 0.14%)	0.00% (0.00% to 0.08%)
AUT	0.02% (0.01% to 0.05%)	0.01% (0.00% to 0.05%)	0.00% (0.00% to 0.04%)	0.00% (0.00% to 0.02%)	0.00% (0.00% to 0.01%)
AZE	0.13% (0.05% to 0.36%)	0.00% (0.00% to 0.03%)	0.00% (0.00% to 0.02%)	0.00% (0.00% to 0.01%)	0.00% (0.00% to 0.01%)
BDI	0.40% (0.17% to 1.1%)	0.14% (0.02% to 1.1%)	0.10% (0.02% to 0.84%)	0.06% (0.01% to 0.46%)	0.03% (0.00% to 0.25%)
BEL	0.03% (0.01% to 0.07%)	0.01% (0.00% to 0.10%)	0.01% (0.00% to 0.08%)	0.01% (0.00% to 0.04%)	0.00% (0.00% to 0.02%)
BEN	0.17% (0.07% to 0.40%)	0.07% (0.01% to 0.42%)	0.06% (0.01% to 0.32%)	0.03% (0.00% to 0.17%)	0.02% (0.00% to 0.10%)
BFA	0.23% (0.09% to 0.57%)	0.20% (0.03% to 1.5%)	0.15% (0.02% to 1.2%)	0.08% (0.01% to 0.66%)	0.04% (0.01% to 0.37%)
BGD	1.1% (0.50% to 3.1%)	1.1% (0.17% to 9.7%)	0.79% (0.13% to 7.4%)	0.44% (0.07% to 4.2%)	0.24% (0.04% to 2.4%)
BGR	0.08% (0.03% to 0.25%)	0.03% (0.00% to 0.30%)	0.02% (0.00% to 0.23%)	0.01% (0.00% to 0.13%)	0.01% (0.00% to 0.07%)
BHR	0.04% (0.02% to 0.11%)	0.02% (0.00% to 0.17%)	0.01% (0.00% to 0.13%)	0.01% (0.00% to 0.07%)	0.00% (0.00% to 0.04%)
BHS	0.02% (0.01% to 0.07%)	0.01% (0.00% to 0.06%)	0.01% (0.00% to 0.05%)	0.00% (0.00% to 0.03%)	0.00% (0.00% to 0.01%)
BIH	0.15% (0.05% to 0.46%)	0.05% (0.01% to 0.58%)	0.04% (0.00% to 0.43%)	0.02% (0.00% to 0.24%)	0.01% (0.00% to 0.13%)
BLR	0.24% (0.08% to 0.73%)	0.17% (0.02% to 2.0%)	0.13% (0.01% to 1.5%)	0.07% (0.01% to 0.84%)	0.04% (0.00% to 0.47%)
BLZ	0.09% (0.04% to 0.27%)	0.05% (0.01% to 0.61%)	0.04% (0.01% to 0.46%)	0.02% (0.00% to 0.26%)	0.01% (0.00% to 0.14%)
BOL	0.39% (0.16% to 1.1%)	0.19% (0.03% to 1.5%)	0.14% (0.02% to 1.1%)	0.08% (0.01% to 0.63%)	0.04% (0.01% to 0.35%)
BRA	0.11% (0.04% to 0.26%)	0.06% (0.01% to 0.42%)	0.04% (0.00% to 0.32%)	0.02% (0.00% to 0.18%)	0.01% (0.00% to 0.10%)
BRB	0.00% (0.00% to 0.00%)	0.00% (0.00% to 0.00%)	0.00% (0.00% to 0.00%)	0.00% (0.00% to 0.00%)	0.00% (0.00% to 0.00%)
BRN	0.21% (0.07% to 0.66%)	0.17% (0.02% to 2.0%)	0.13% (0.01% to 1.5%)	0.07% (0.01% to 0.85%)	0.04% (0.00% to 0.47%)
BTN	0.25% (0.10% to 0.68%)	0.03% (0.00% to 0.29%)	0.02% (0.00% to 0.22%)	0.01% (0.00% to 0.12%)	0.01% (0.00% to 0.06%)
BWA	0.71% (0.32% to 1.7%)	0.28% (0.05% to 2.7%)	0.20% (0.03% to 2.0%)	0.11% (0.02% to 1.1%)	0.06% (0.01% to 0.63%)
CAF	0.73% (0.26% to 1.9%)	0.15% (0.02% to 1.2%)	0.11% (0.01% to 0.90%)	0.06% (0.01% to 0.50%)	0.03% (0.00% to 0.27%)
CHE	0.01% (0.01% to 0.04%)	0.01% (0.00% to 0.07%)	0.00% (0.00% to 0.05%)	0.00% (0.00% to 0.03%)	0.00% (0.00% to 0.02%)
CHL	0.03% (0.01% to 0.09%)	0.01% (0.00% to 0.16%)	0.01% (0.00% to 0.12%)	0.01% (0.00% to 0.07%)	0.00% (0.00% to 0.04%)
CIV	0.49% (0.19% to 1.3%)	0.16% (0.02% to 1.4%)	0.12% (0.01% to 1.0%)	0.06% (0.01% to 0.58%)	0.03% (0.00% to 0.32%)
CMR	0.82% (0.34% to 2.1%)	0.52% (0.08% to 4.5%)	0.38% (0.06% to 3.4%)	0.21% (0.03% to 1.9%)	0.11% (0.02% to 1.1%)
COD	1.5% (0.54% to 3.3%)	1.5% (0.17% to 9.8%)	1.1% (0.12% to 7.4%)	0.63% (0.07% to 4.2%)	0.34% (0.04% to 2.3%)
COG	1.6% (0.59% to 4.6%)	2.7% (0.28% to 25.9%)	2.0% (0.21% to 19.7%)	1.1% (0.11% to 11.3%)	0.62% (0.06% to 6.4%)
COL	0.08% (0.03% to 0.22%)	0.04% (0.00% to 0.33%)	0.03% (0.00% to 0.25%)	0.01% (0.00% to 0.14%)	0.01% (0.00% to 0.08%)
COM	0.16% (0.06% to 0.44%)	0.11% (0.02% to 1.3%)	0.08% (0.01% to 1.0%)	0.04% (0.01% to 0.57%)	0.02% (0.00% to 0.32%)
CRI	0.02% (0.01% to 0.08%)	0.01% (0.00% to 0.12%)	0.01% (0.00% to 0.09%)	0.00% (0.00% to 0.05%)	0.00% (0.00% to 0.03%)
CUB	0.02% (0.01% to 0.06%)	0.01% (0.00% to 0.07%)	0.01% (0.00% to 0.05%)	0.00% (0.00% to 0.03%)	0.00% (0.00% to 0.02%)
CYP	0.02% (0.01% to 0.07%)	0.03% (0.00% to 0.30%)	0.02% (0.00% to 0.22%)	0.01% (0.00% to 0.13%)	0.01% (0.00% to 0.07%)
CZE	0.01% (0.00% to 0.03%)	0.00% (0.00% to 0.01%)	0.00% (0.00% to 0.01%)	0.00% (0.00% to 0.01%)	0.00% (0.00% to 0.00%)
DEU	0.01% (0.00% to 0.04%)	0.00% (0.00% to 0.04%)	0.00% (0.00% to 0.03%)	0.00% (0.00% to 0.02%)	0.00% (0.00% to 0.01%)
DJI	2.5% (1.0% to 6.7%)	3.7% (0.55% to 25.9%)	2.8% (0.41% to 19.7%)	1.6% (0.22% to 11.3%)	0.86% (0.12% to 6.4%)
DMA	0.00% (0.00% to 0.01%)	0.00% (0.00% to 0.01%)	0.00% (0.00% to 0.01%)	0.00% (0.00% to 0.00%)	0.00% (0.00% to 0.00%)
DNK	0.02% (0.01% to 0.06%)	0.01% (0.00% to 0.13%)	0.01% (0.00% to 0.10%)	0.00% (0.00% to 0.05%)	0.00% (0.00% to 0.03%)
DOM	0.14% (0.05% to 0.36%)	0.04% (0.01% to 0.32%)	0.03% (0.00% to 0.24%)	0.02% (0.00% to 0.13%)	0.01% (0.00% to 0.07%)
DZA	0.22% (0.10% to 0.49%)	0.13% (0.02% to 0.69%)	0.10% (0.02% to 0.52%)	0.05% (0.01% to 0.29%)	0.03% (0.00% to 0.16%)

ISO3 Code	Status quo		2050 (95% UI) with additional reduction in ARI		
	2024 (95% UI)	2050 (95% UI)	Additional 1% reduction	Additional 3% reduction	Additional 5% reduction
ECU	0.16% (0.06% to 0.45%)	0.06% (0.01% to 0.53%)	0.04% (0.00% to 0.40%)	0.02% (0.00% to 0.22%)	0.01% (0.00% to 0.12%)
EGY	0.05% (0.02% to 0.15%)	0.03% (0.00% to 0.19%)	0.02% (0.00% to 0.15%)	0.01% (0.00% to 0.08%)	0.01% (0.00% to 0.04%)
ERI	0.28% (0.11% to 0.70%)	0.12% (0.02% to 0.90%)	0.09% (0.01% to 0.67%)	0.05% (0.01% to 0.37%)	0.03% (0.00% to 0.21%)
ESP	0.03% (0.01% to 0.11%)	0.01% (0.00% to 0.17%)	0.01% (0.00% to 0.13%)	0.01% (0.00% to 0.07%)	0.00% (0.00% to 0.04%)
EST	0.06% (0.02% to 0.15%)	0.01% (0.00% to 0.12%)	0.01% (0.00% to 0.09%)	0.01% (0.00% to 0.05%)	0.00% (0.00% to 0.03%)
ETH	0.40% (0.18% to 0.94%)	0.14% (0.02% to 0.95%)	0.10% (0.02% to 0.71%)	0.06% (0.01% to 0.39%)	0.03% (0.00% to 0.22%)
FIN	0.01% (0.01% to 0.04%)	0.00% (0.00% to 0.04%)	0.00% (0.00% to 0.03%)	0.00% (0.00% to 0.02%)	0.00% (0.00% to 0.01%)
FJI	0.24% (0.08% to 0.57%)	0.19% (0.01% to 1.2%)	0.14% (0.01% to 0.93%)	0.08% (0.01% to 0.52%)	0.04% (0.00% to 0.29%)
FRA	0.03% (0.01% to 0.07%)	0.01% (0.00% to 0.10%)	0.01% (0.00% to 0.08%)	0.01% (0.00% to 0.04%)	0.00% (0.00% to 0.02%)
GAB	1.8% (0.63% to 4.3%)	1.7% (0.21% to 13.4%)	1.3% (0.16% to 10.2%)	0.70% (0.08% to 5.8%)	0.38% (0.04% to 3.2%)
GBR	0.05% (0.02% to 0.11%)	0.06% (0.01% to 0.36%)	0.04% (0.00% to 0.27%)	0.02% (0.00% to 0.15%)	0.01% (0.00% to 0.09%)
GEO	0.25% (0.07% to 0.66%)	0.04% (0.00% to 0.36%)	0.03% (0.00% to 0.26%)	0.02% (0.00% to 0.14%)	0.01% (0.00% to 0.08%)
GHA	0.66% (0.27% to 2.3%)	0.36% (0.04% to 4.2%)	0.27% (0.03% to 3.1%)	0.15% (0.02% to 1.8%)	0.08% (0.01% to 0.97%)
GIN	0.37% (0.15% to 1.2%)	0.18% (0.02% to 2.0%)	0.13% (0.01% to 1.5%)	0.07% (0.01% to 0.85%)	0.04% (0.00% to 0.47%)
GMB	0.30% (0.15% to 0.66%)	0.21% (0.05% to 1.1%)	0.16% (0.04% to 0.82%)	0.09% (0.02% to 0.46%)	0.05% (0.01% to 0.26%)
GNB	1.2% (0.43% to 3.3%)	1.3% (0.12% to 9.8%)	0.95% (0.09% to 7.4%)	0.53% (0.05% to 4.2%)	0.29% (0.03% to 2.3%)
GRC	0.01% (0.00% to 0.04%)	0.01% (0.00% to 0.10%)	0.00% (0.00% to 0.08%)	0.00% (0.00% to 0.04%)	0.00% (0.00% to 0.02%)
GRD	0.00% (0.00% to 0.01%)	0.00% (0.00% to 0.03%)	0.00% (0.00% to 0.02%)	0.00% (0.00% to 0.01%)	0.00% (0.00% to 0.01%)
GTM	0.28% (0.12% to 0.70%)	0.21% (0.03% to 1.7%)	0.15% (0.02% to 1.3%)	0.08% (0.01% to 0.74%)	0.05% (0.01% to 0.42%)
GUY	0.30% (0.11% to 0.80%)	0.18% (0.02% to 1.6%)	0.13% (0.02% to 1.2%)	0.07% (0.01% to 0.69%)	0.04% (0.00% to 0.39%)
HKG	0.21% (0.07% to 0.57%)	0.11% (0.01% to 1.1%)	0.08% (0.01% to 0.83%)	0.04% (0.00% to 0.46%)	0.02% (0.00% to 0.26%)
HND	0.09% (0.03% to 0.30%)	0.02% (0.00% to 0.25%)	0.01% (0.00% to 0.19%)	0.01% (0.00% to 0.10%)	0.00% (0.00% to 0.06%)
HRV	0.03% (0.01% to 0.09%)	0.00% (0.00% to 0.05%)	0.00% (0.00% to 0.03%)	0.00% (0.00% to 0.02%)	0.00% (0.00% to 0.01%)
HTI	0.59% (0.22% to 1.7%)	0.34% (0.03% to 3.0%)	0.25% (0.02% to 2.3%)	0.14% (0.01% to 1.3%)	0.07% (0.01% to 0.71%)
HUN	0.03% (0.01% to 0.07%)	0.01% (0.00% to 0.04%)	0.00% (0.00% to 0.03%)	0.00% (0.00% to 0.01%)	0.00% (0.00% to 0.01%)
IDN	1.6% (0.72% to 3.6%)	1.1% (0.19% to 5.7%)	0.84% (0.14% to 4.3%)	0.46% (0.07% to 2.4%)	0.25% (0.04% to 1.3%)
IRL	0.02% (0.01% to 0.06%)	0.01% (0.00% to 0.10%)	0.01% (0.00% to 0.08%)	0.00% (0.00% to 0.04%)	0.00% (0.00% to 0.02%)
IRN	0.07% (0.03% to 0.20%)	0.05% (0.01% to 0.39%)	0.04% (0.00% to 0.29%)	0.02% (0.00% to 0.17%)	0.01% (0.00% to 0.09%)
IRQ	0.19% (0.07% to 0.61%)	0.27% (0.03% to 3.2%)	0.20% (0.02% to 2.5%)	0.11% (0.01% to 1.4%)	0.06% (0.01% to 0.80%)
ISL	0.01% (0.00% to 0.03%)	0.01% (0.00% to 0.06%)	0.00% (0.00% to 0.05%)	0.00% (0.00% to 0.03%)	0.00% (0.00% to 0.01%)
ISR	0.01% (0.00% to 0.04%)	0.01% (0.00% to 0.04%)	0.00% (0.00% to 0.03%)	0.00% (0.00% to 0.02%)	0.00% (0.00% to 0.01%)
ITA	0.02% (0.01% to 0.05%)	0.01% (0.00% to 0.09%)	0.01% (0.00% to 0.06%)	0.00% (0.00% to 0.04%)	0.00% (0.00% to 0.02%)
JAM	0.02% (0.01% to 0.04%)	0.01% (0.00% to 0.08%)	0.01% (0.00% to 0.06%)	0.00% (0.00% to 0.03%)	0.00% (0.00% to 0.02%)
JOR	0.02% (0.01% to 0.04%)	0.01% (0.00% to 0.08%)	0.01% (0.00% to 0.06%)	0.00% (0.00% to 0.03%)	0.00% (0.00% to 0.02%)
JPN	0.05% (0.02% to 0.14%)	0.02% (0.00% to 0.18%)	0.01% (0.00% to 0.14%)	0.01% (0.00% to 0.08%)	0.00% (0.00% to 0.04%)
KAZ	0.42% (0.15% to 1.2%)	0.33% (0.04% to 2.9%)	0.24% (0.03% to 2.2%)	0.13% (0.01% to 1.3%)	0.07% (0.01% to 0.71%)
KEN	0.76% (0.28% to 1.8%)	1.0% (0.12% to 6.7%)	0.79% (0.09% to 5.0%)	0.44% (0.05% to 2.9%)	0.24% (0.03% to 1.6%)
KGZ	0.48% (0.20% to 1.3%)	0.21% (0.03% to 1.9%)	0.15% (0.02% to 1.5%)	0.08% (0.01% to 0.81%)	0.04% (0.01% to 0.44%)
KHM	1.3% (0.58% to 3.4%)	0.39% (0.07% to 3.2%)	0.29% (0.05% to 2.4%)	0.15% (0.03% to 1.3%)	0.08% (0.01% to 0.73%)
KNA	0.03% (0.01% to 0.08%)	0.04% (0.00% to 0.31%)	0.03% (0.00% to 0.24%)	0.01% (0.00% to 0.14%)	0.01% (0.00% to 0.08%)
KOR	0.18% (0.07% to 0.59%)	0.10% (0.01% to 1.0%)	0.08% (0.01% to 0.77%)	0.04% (0.00% to 0.43%)	0.02% (0.00% to 0.24%)
KWT	0.07% (0.03% to 0.15%)	0.04% (0.01% to 0.24%)	0.03% (0.00% to 0.18%)	0.02% (0.00% to 0.10%)	0.01% (0.00% to 0.06%)
LAO	0.83% (0.32% to 2.2%)	0.26% (0.03% to 2.0%)	0.19% (0.02% to 1.5%)	0.10% (0.01% to 0.81%)	0.06% (0.01% to 0.44%)
LBN	0.05% (0.01% to 0.12%)	0.04% (0.00% to 0.37%)	0.03% (0.00% to 0.28%)	0.02% (0.00% to 0.16%)	0.01% (0.00% to 0.09%)
LBR	1.5% (0.61% to 4.5%)	2.2% (0.34% to 21.5%)	1.6% (0.25% to 16.3%)	0.91% (0.14% to 9.4%)	0.50% (0.07% to 5.3%)
LBY	0.13% (0.05% to 0.33%)	0.10% (0.02% to 0.81%)	0.08% (0.01% to 0.61%)	0.04% (0.01% to 0.34%)	0.02% (0.00% to 0.19%)
LCA	0.02% (0.01% to 0.05%)	0.01% (0.00% to 0.07%)	0.01% (0.00% to 0.05%)	0.00% (0.00% to 0.03%)	0.00% (0.00% to 0.02%)
LKA	0.25% (0.10% to 0.69%)	0.22% (0.03% to 1.8%)	0.16% (0.02% to 1.4%)	0.09% (0.01% to 0.77%)	0.05% (0.01% to 0.43%)

ISO3	Status quo		2050 (95% UI) with additional reduction in ARI		
Code	2024 (95% UI)	2050 (95% UI)	Additional 1% reduction	Additional 3% reduction	Additional 5% reduction
LTU	0.22% (0.08% to 0.54%)	0.11% (0.01% to 0.87%)	0.08% (0.01% to 0.65%)	0.05% (0.01% to 0.37%)	0.02% (0.00% to 0.20%)
LUX	0.02% (0.01% to 0.05%)	0.01% (0.00% to 0.07%)	0.01% (0.00% to 0.05%)	0.00% (0.00% to 0.03%)	0.00% (0.00% to 0.02%)
LVA	0.10% (0.03% to 0.28%)	0.02% (0.00% to 0.17%)	0.01% (0.00% to 0.13%)	0.01% (0.00% to 0.07%)	0.00% (0.00% to 0.04%)
MAC	0.25% (0.09% to 0.87%)	0.16% (0.02% to 1.5%)	0.12% (0.01% to 1.1%)	0.06% (0.01% to 0.63%)	0.03% (0.00% to 0.35%)
MAR	0.32% (0.11% to 0.82%)	0.25% (0.03% to 2.1%)	0.19% (0.02% to 1.6%)	0.10% (0.01% to 0.88%)	0.06% (0.01% to 0.49%)
MDA	0.74% (0.30% to 2.1%)	0.83% (0.11% to 7.3%)	0.62% (0.08% to 5.6%)	0.34% (0.04% to 3.2%)	0.19% (0.02% to 1.8%)
MDG	0.92% (0.31% to 2.6%)	0.53% (0.05% to 3.8%)	0.39% (0.04% to 2.8%)	0.21% (0.02% to 1.6%)	0.11% (0.01% to 0.88%)
MEX	0.04% (0.02% to 0.16%)	0.02% (0.00% to 0.22%)	0.01% (0.00% to 0.17%)	0.01% (0.00% to 0.09%)	0.00% (0.00% to 0.05%)
MKD	0.04% (0.02% to 0.12%)	0.02% (0.00% to 0.14%)	0.01% (0.00% to 0.10%)	0.01% (0.00% to 0.06%)	0.00% (0.00% to 0.03%)
MLI	0.22% (0.09% to 0.65%)	0.15% (0.02% to 1.1%)	0.11% (0.01% to 0.86%)	0.06% (0.01% to 0.49%)	0.03% (0.00% to 0.27%)
MLT	0.05% (0.02% to 0.18%)	0.14% (0.02% to 2.2%)	0.10% (0.02% to 1.7%)	0.06% (0.01% to 0.98%)	0.03% (0.01% to 0.57%)
MMR	0.99% (0.39% to 2.2%)	0.44% (0.05% to 2.4%)	0.33% (0.04% to 1.8%)	0.18% (0.02% to 1.0%)	0.10% (0.01% to 0.55%)
MNE	0.05% (0.02% to 0.11%)	0.01% (0.00% to 0.05%)	0.01% (0.00% to 0.04%)	0.00% (0.00% to 0.02%)	0.00% (0.00% to 0.01%)
MNG	0.39% (0.13% to 1.1%)	0.12% (0.01% to 1.3%)	0.09% (0.01% to 0.96%)	0.05% (0.00% to 0.53%)	0.03% (0.00% to 0.29%)
MOZ	1.4% (0.50% to 3.4%)	0.95% (0.14% to 6.7%)	0.70% (0.11% to 5.0%)	0.39% (0.06% to 2.8%)	0.21% (0.03% to 1.6%)
MRT	0.30% (0.11% to 0.82%)	0.08% (0.01% to 0.68%)	0.06% (0.01% to 0.51%)	0.03% (0.00% to 0.28%)	0.02% (0.00% to 0.16%)
MUS	0.09% (0.04% to 0.22%)	0.06% (0.01% to 0.41%)	0.05% (0.01% to 0.31%)	0.03% (0.00% to 0.17%)	0.01% (0.00% to 0.10%)
MWI	1.00% (0.33% to 2.7%)	1.1% (0.10% to 8.3%)	0.86% (0.08% to 6.3%)	0.48% (0.04% to 3.6%)	0.26% (0.02% to 2.0%)
MYS	0.36% (0.14% to 0.89%)	0.48% (0.05% to 3.4%)	0.36% (0.04% to 2.6%)	0.20% (0.02% to 1.5%)	0.11% (0.01% to 0.85%)
NAM	2.1% (0.77% to 5.3%)	1.9% (0.24% to 15.1%)	1.4% (0.18% to 11.4%)	0.77% (0.10% to 6.5%)	0.42% (0.05% to 3.6%)
NER	0.23% (0.09% to 0.63%)	0.05% (0.00% to 0.48%)	0.04% (0.00% to 0.36%)	0.02% (0.00% to 0.20%)	0.01% (0.00% to 0.11%)
NGA	0.84% (0.42% to 1.9%)	0.87% (0.17% to 4.7%)	0.65% (0.13% to 3.5%)	0.36% (0.07% to 2.0%)	0.20% (0.04% to 1.1%)
NIC	0.08% (0.03% to 0.23%)	0.07% (0.01% to 0.86%)	0.05% (0.01% to 0.65%)	0.03% (0.00% to 0.37%)	0.02% (0.00% to 0.21%)
NLD	0.02% (0.01% to 0.04%)	0.01% (0.00% to 0.08%)	0.01% (0.00% to 0.06%)	0.00% (0.00% to 0.03%)	0.00% (0.00% to 0.02%)
NOR	0.03% (0.01% to 0.09%)	0.03% (0.00% to 0.39%)	0.02% (0.00% to 0.30%)	0.01% (0.00% to 0.17%)	0.01% (0.00% to 0.10%)
NPL	0.50% (0.17% to 1.4%)	0.43% (0.03% to 3.1%)	0.32% (0.02% to 2.4%)	0.17% (0.01% to 1.3%)	0.10% (0.01% to 0.75%)
NZL	0.02% (0.01% to 0.06%)	0.01% (0.00% to 0.14%)	0.01% (0.00% to 0.10%)	0.01% (0.00% to 0.06%)	0.00% (0.00% to 0.03%)
OMN	0.03% (0.01% to 0.08%)	0.02% (0.00% to 0.14%)	0.01% (0.00% to 0.11%)	0.01% (0.00% to 0.06%)	0.00% (0.00% to 0.03%)
PAK	0.81% (0.36% to 1.7%)	0.51% (0.10% to 2.3%)	0.38% (0.07% to 1.8%)	0.21% (0.04% to 0.98%)	0.11% (0.02% to 0.54%)
PAN	0.17% (0.06% to 0.45%)	0.14% (0.02% to 1.2%)	0.10% (0.01% to 0.89%)	0.06% (0.01% to 0.51%)	0.03% (0.00% to 0.28%)
PER	0.35% (0.14% to 1.2%)	0.16% (0.02% to 1.8%)	0.12% (0.02% to 1.3%)	0.06% (0.01% to 0.75%)	0.03% (0.00% to 0.42%)
POL	0.05% (0.02% to 0.15%)	0.02% (0.00% to 0.18%)	0.02% (0.00% to 0.14%)	0.01% (0.00% to 0.08%)	0.00% (0.00% to 0.04%)
PRT	0.05% (0.02% to 0.15%)	0.02% (0.00% to 0.18%)	0.01% (0.00% to 0.14%)	0.01% (0.00% to 0.08%)	0.00% (0.00% to 0.04%)
PRY	0.14% (0.05% to 0.42%)	0.09% (0.01% to 1.0%)	0.07% (0.01% to 0.77%)	0.04% (0.00% to 0.44%)	0.02% (0.00% to 0.24%)
PSE	0.02% (0.00% to 0.05%)	0.01% (0.00% to 0.11%)	0.01% (0.00% to 0.08%)	0.00% (0.00% to 0.05%)	0.00% (0.00% to 0.02%)
ROU	0.18% (0.07% to 0.47%)	0.04% (0.00% to 0.32%)	0.03% (0.00% to 0.24%)	0.02% (0.00% to 0.13%)	0.01% (0.00% to 0.07%)
RUS	0.33% (0.11% to 0.87%)	0.21% (0.02% to 2.0%)	0.15% (0.01% to 1.5%)	0.08% (0.01% to 0.84%)	0.05% (0.00% to 0.47%)
RWA	0.24% (0.10% to 0.48%)	0.22% (0.03% to 0.99%)	0.17% (0.02% to 0.75%)	0.09% (0.01% to 0.42%)	0.05% (0.01% to 0.23%)
SAU	0.04% (0.01% to 0.14%)	0.03% (0.00% to 0.36%)	0.02% (0.00% to 0.27%)	0.01% (0.00% to 0.15%)	0.01% (0.00% to 0.09%)
SDN	0.33% (0.11% to 0.85%)	0.19% (0.02% to 1.4%)	0.14% (0.02% to 1.1%)	0.08% (0.01% to 0.59%)	0.04% (0.00% to 0.33%)
SEN	0.50% (0.17% to 1.2%)	0.35% (0.04% to 2.5%)	0.26% (0.03% to 1.9%)	0.14% (0.02% to 1.1%)	0.08% (0.01% to 0.59%)
SGP	0.14% (0.04% to 0.33%)	0.10% (0.01% to 0.80%)	0.08% (0.01% to 0.60%)	0.04% (0.00% to 0.34%)	0.02% (0.00% to 0.19%)
SLE	1.2% (0.43% to 3.2%)	0.85% (0.09% to 9.0%)	0.63% (0.07% to 6.8%)	0.35% (0.04% to 3.9%)	0.19% (0.02% to 2.2%)
SLV	0.11% (0.03% to 0.32%)	0.13% (0.01% to 1.5%)	0.09% (0.01% to 1.1%)	0.05% (0.00% to 0.63%)	0.03% (0.00% to 0.36%)
SOM	1.1% (0.50% to 3.7%)	0.90% (0.14% to 11.2%)	0.67% (0.10% to 8.4%)	0.37% (0.06% to 4.8%)	0.20% (0.03% to 2.7%)
SRB	0.06% (0.02% to 0.21%)	0.01% (0.00% to 0.24%)	0.01% (0.00% to 0.18%)	0.01% (0.00% to 0.10%)	0.00% (0.00% to 0.06%)
SSD	0.82% (0.23% to 2.2%)	1.2% (0.08% to 8.9%)	0.88% (0.06% to 6.7%)	0.49% (0.03% to 3.8%)	0.27% (0.02% to 2.2%)
SUR	0.07% (0.03% to 0.20%)	0.01% (0.00% to 0.11%)	0.01% (0.00% to 0.08%)	0.00% (0.00% to 0.05%)	0.00% (0.00% to 0.02%)

ISO3	Status quo		2050 (95% UI) with additional reduction in ARI		
Code	2024 (95% UI)	2050 (95% UI)	Additional 1% reduction	Additional 3% reduction	Additional 5% reduction
SVK	0.01% (0.00% to 0.04%)	0.00% (0.00% to 0.02%)	0.00% (0.00% to 0.02%)	0.00% (0.00% to 0.01%)	0.00% (0.00% to 0.00%)
SVN	0.02% (0.01% to 0.06%)	0.00% (0.00% to 0.04%)	0.00% (0.00% to 0.03%)	0.00% (0.00% to 0.02%)	0.00% (0.00% to 0.01%)
SWE	0.02% (0.01% to 0.09%)	0.04% (0.00% to 0.52%)	0.03% (0.00% to 0.39%)	0.02% (0.00% to 0.23%)	0.01% (0.00% to 0.13%)
SWZ	2.1% (0.74% to 5.0%)	3.7% (0.48% to 23.0%)	2.8% (0.35% to 17.5%)	1.6% (0.19% to 10.0%)	0.87% (0.10% to 5.7%)
SYC	0.07% (0.02% to 0.20%)	0.05% (0.01% to 0.52%)	0.03% (0.00% to 0.40%)	0.02% (0.00% to 0.22%)	0.01% (0.00% to 0.13%)
SYR	0.04% (0.01% to 0.12%)	0.01% (0.00% to 0.14%)	0.01% (0.00% to 0.11%)	0.00% (0.00% to 0.06%)	0.00% (0.00% to 0.03%)
TCD	0.55% (0.18% to 1.5%)	0.51% (0.05% to 4.7%)	0.38% (0.03% to 3.6%)	0.21% (0.02% to 2.0%)	0.11% (0.01% to 1.1%)
TGO	0.22% (0.07% to 0.53%)	0.38% (0.04% to 2.8%)	0.28% (0.03% to 2.1%)	0.16% (0.01% to 1.2%)	0.09% (0.01% to 0.69%)
THA	0.62% (0.27% to 1.3%)	0.38% (0.07% to 1.9%)	0.28% (0.05% to 1.5%)	0.16% (0.03% to 0.81%)	0.08% (0.01% to 0.44%)
TJK	0.34% (0.13% to 0.80%)	0.09% (0.01% to 0.69%)	0.07% (0.01% to 0.52%)	0.04% (0.00% to 0.28%)	0.02% (0.00% to 0.15%)
TKM	0.23% (0.07% to 0.67%)	0.04% (0.00% to 0.43%)	0.03% (0.00% to 0.32%)	0.02% (0.00% to 0.17%)	0.01% (0.00% to 0.09%)
TTO	0.07% (0.03% to 0.19%)	0.13% (0.01% to 0.89%)	0.10% (0.01% to 0.68%)	0.05% (0.01% to 0.39%)	0.03% (0.00% to 0.22%)
TUN	0.12% (0.04% to 0.31%)	0.16% (0.02% to 1.3%)	0.12% (0.01% to 0.96%)	0.07% (0.01% to 0.55%)	0.04% (0.00% to 0.31%)
TUR	0.04% (0.02% to 0.10%)	0.01% (0.00% to 0.10%)	0.01% (0.00% to 0.07%)	0.00% (0.00% to 0.04%)	0.00% (0.00% to 0.02%)
TZA	1.5% (0.55% to 4.0%)	1.6% (0.22% to 11.8%)	1.2% (0.16% to 8.9%)	0.66% (0.09% to 5.0%)	0.36% (0.05% to 2.8%)
UGA	0.22% (0.10% to 0.63%)	0.07% (0.01% to 0.55%)	0.05% (0.01% to 0.41%)	0.03% (0.00% to 0.23%)	0.02% (0.00% to 0.12%)
UKR	0.34% (0.12% to 0.87%)	0.24% (0.03% to 1.8%)	0.18% (0.02% to 1.4%)	0.10% (0.01% to 0.78%)	0.05% (0.01% to 0.43%)
URY	0.08% (0.03% to 0.25%)	0.10% (0.01% to 0.79%)	0.08% (0.01% to 0.60%)	0.04% (0.00% to 0.34%)	0.02% (0.00% to 0.19%)
USA	0.01% (0.00% to 0.02%)	0.00% (0.00% to 0.02%)	0.00% (0.00% to 0.01%)	0.00% (0.00% to 0.01%)	0.00% (0.00% to 0.00%)
UZB	0.41% (0.13% to 0.97%)	0.41% (0.05% to 2.6%)	0.31% (0.03% to 2.0%)	0.17% (0.02% to 1.1%)	0.09% (0.01% to 0.63%)
VCT	0.07% (0.03% to 0.21%)	0.03% (0.00% to 0.35%)	0.02% (0.00% to 0.27%)	0.01% (0.00% to 0.15%)	0.01% (0.00% to 0.08%)
VEN	0.07% (0.02% to 0.19%)	0.05% (0.00% to 0.44%)	0.04% (0.00% to 0.33%)	0.02% (0.00% to 0.19%)	0.01% (0.00% to 0.10%)
YEM	0.10% (0.04% to 0.27%)	0.02% (0.00% to 0.19%)	0.01% (0.00% to 0.14%)	0.01% (0.00% to 0.08%)	0.00% (0.00% to 0.04%)
ZAF	2.2% (0.79% to 5.3%)	3.5% (0.36% to 23.3%)	2.6% (0.26% to 17.7%)	1.5% (0.14% to 10.2%)	0.81% (0.08% to 5.8%)
ZMB	0.87% (0.31% to 2.3%)	0.40% (0.05% to 4.5%)	0.30% (0.04% to 3.4%)	0.16% (0.02% to 1.9%)	0.09% (0.01% to 1.0%)
ZWE	0.62% (0.25% to 1.7%)	0.36% (0.05% to 2.9%)	0.27% (0.04% to 2.2%)	0.15% (0.02% to 1.2%)	0.08% (0.01% to 0.67%)

Appendix Table 4. Projected prevalence of Mtb immunoreactivity infection among immigrants under the status quo scenario for selected years (2024, 2030, 2040, and 2050), China, India, Philippines, Viet Nam

Country	2024 (95% UI)	2030 (95% UI)	2040 (95% UI)	2050 (95% UI)
China	14.7% (10.7% to 22.7%)	12.2% (8.9% to 16.8%)	8.5% (5.9% to 13.3%)	5.8% (3.4% to 13.5%)
India	25.4% (20.4% to 30.8%)	22.5% (17.2% to 29.0%)	17.6% (12.0% to 27.6%)	13.2% (7.7% to 26.2%)
Philippines	40.1% (32.6% to 49.8%)	36.6% (28.7% to 47.7%)	29.4% (21.3% to 46.2%)	23.0% (14.3% to 48.5%)
Viet Nam	27.7% (19.5% to 41.2%)	23.6% (17.1% to 33.0%)	17.4% (11.6% to 28.5%)	11.7% (6.9% to 30.5%)

Appendix Table 5. Projected prevalence of Mtb immunoreactivity infection among immigrants under the status quo scenario for selected years (2024, 2030, 2040, and 2050), all other countries

ISO3 Code	2024 (95% UI)	2030 (95% UI)	2040 (95% UI)	2050 (95% UI)
AFG	28.8% (23.5% to 37.7%)	26.2% (20.7% to 36.2%)	21.6% (14.7% to 37.2%)	18.5% (9.5% to 41.7%)
AGO	36.4% (28.1% to 46.5%)	36.0% (26.3% to 51.5%)	36.1% (21.3% to 63.4%)	35.5% (15.5% to 78.4%)
ALB	2.7% (1.6% to 10.3%)	2.5% (1.6% to 6.5%)	2.1% (1.1% to 4.5%)	1.6% (0.70% to 5.7%)
ARE	3.6% (0.97% to 14.2%)	2.2% (0.71% to 8.9%)	0.87% (0.40% to 3.7%)	0.34% (0.20% to 1.2%)
ARG	5.3% (3.7% to 7.9%)	4.1% (2.9% to 5.9%)	2.7% (1.7% to 5.1%)	1.8% (1.0% to 5.6%)
ARM	6.2% (4.5% to 9.5%)	6.3% (4.3% to 10.1%)	6.2% (3.3% to 14.5%)	5.8% (2.3% to 18.5%)
ATG	0.57% (0.38% to 1.7%)	0.59% (0.38% to 1.2%)	0.60% (0.32% to 1.9%)	0.61% (0.23% to 3.3%)
AUS	0.86% (0.55% to 2.1%)	0.84% (0.52% to 1.7%)	0.73% (0.41% to 2.2%)	0.68% (0.28% to 4.0%)
AUT	2.5% (1.2% to 11.4%)	1.9% (1.1% to 7.3%)	1.2% (0.71% to 3.1%)	0.76% (0.39% to 1.6%)
AZE	46.1% (34.4% to 56.8%)	36.0% (26.6% to 45.2%)	21.3% (15.2% to 29.9%)	11.1% (7.2% to 15.9%)
BDI	24.2% (17.3% to 34.9%)	20.8% (15.4% to 28.8%)	15.7% (10.8% to 26.8%)	11.6% (6.4% to 28.8%)
BEL	2.0% (1.1% to 8.5%)	1.7% (1.0% to 4.8%)	1.3% (0.71% to 2.7%)	0.86% (0.47% to 2.2%)
BEN	13.8% (8.0% to 26.8%)	11.4% (7.0% to 20.2%)	8.3% (4.7% to 13.6%)	5.7% (3.0% to 11.7%)
BFA	8.3% (6.0% to 17.6%)	8.2% (5.7% to 14.0%)	7.5% (4.4% to 16.6%)	6.8% (2.9% to 20.3%)
BGD	30.7% (25.0% to 41.5%)	30.3% (23.0% to 46.1%)	29.4% (18.8% to 57.0%)	28.5% (14.1% to 71.8%)
BGR	6.4% (4.3% to 15.0%)	5.6% (3.8% to 11.4%)	4.1% (2.4% to 7.5%)	2.6% (1.4% to 7.7%)
BHR	4.0% (2.7% to 7.3%)	3.2% (2.2% to 5.4%)	2.2% (1.5% to 3.7%)	1.5% (0.81% to 4.0%)
BHS	2.8% (1.4% to 11.0%)	2.2% (1.3% to 7.3%)	1.4% (0.86% to 3.3%)	0.90% (0.50% to 2.3%)
BIH	11.3% (7.3% to 23.3%)	9.5% (6.4% to 16.1%)	7.0% (4.5% to 12.8%)	4.8% (2.6% to 14.2%)
BLR	8.5% (6.1% to 13.6%)	8.3% (5.7% to 14.5%)	7.5% (4.3% to 19.0%)	6.9% (3.0% to 26.4%)
BLZ	4.5% (3.0% to 11.9%)	4.1% (2.8% to 8.1%)	3.5% (2.1% to 8.3%)	2.7% (1.4% to 10.0%)
BOL	22.1% (16.5% to 36.1%)	19.7% (14.4% to 28.7%)	14.7% (10.1% to 26.8%)	11.0% (6.3% to 29.9%)
BRA	7.8% (5.4% to 15.1%)	6.6% (4.4% to 11.8%)	4.9% (2.9% to 9.1%)	3.6% (1.7% to 9.9%)
BRB	0.23% (0.12% to 2.1%)	0.17% (0.10% to 0.98%)	0.11% (0.07% to 0.30%)	0.06% (0.03% to 0.12%)
BRN	8.1% (5.4% to 17.0%)	8.0% (5.2% to 13.6%)	7.0% (4.1% to 18.6%)	6.1% (2.8% to 26.7%)
BTN	42.2% (31.8% to 51.6%)	33.1% (24.7% to 41.6%)	20.4% (14.3% to 29.8%)	11.6% (7.5% to 20.4%)
BWA	35.1% (28.0% to 44.7%)	31.0% (24.3% to 41.2%)	24.3% (17.1% to 41.9%)	18.9% (11.0% to 45.7%)
CAF	47.8% (37.8% to 56.2%)	40.4% (32.1% to 48.5%)	28.9% (21.4% to 43.7%)	19.7% (12.8% to 40.3%)
CHE	1.7% (0.84% to 8.8%)	1.3% (0.69% to 5.6%)	0.90% (0.50% to 2.3%)	0.57% (0.30% to 1.5%)
CHL	4.1% (2.0% to 15.6%)	3.0% (1.7% to 10.0%)	2.1% (1.2% to 4.5%)	1.3% (0.67% to 3.5%)
CIV	31.2% (23.3% to 42.2%)	26.8% (20.5% to 35.3%)	19.2% (14.1% to 33.1%)	13.9% (8.1% to 33.0%)
CMR	26.3% (20.5% to 33.7%)	25.3% (18.3% to 36.7%)	23.5% (14.3% to 42.5%)	20.4% (10.3% to 52.5%)
COD	38.4% (30.5% to 49.9%)	36.8% (28.8% to 50.9%)	35.8% (22.5% to 63.8%)	35.4% (15.9% to 75.2%)
COG	31.9% (22.9% to 44.3%)	33.8% (22.6% to 54.5%)	37.7% (19.1% to 78.5%)	42.4% (15.2% to 91.8%)
COL	6.2% (3.9% to 16.5%)	5.4% (3.6% to 11.4%)	3.9% (2.6% to 7.2%)	2.7% (1.5% to 7.3%)
COM	7.4% (4.9% to 17.1%)	6.8% (4.6% to 13.1%)	5.9% (3.2% to 12.8%)	4.9% (2.1% to 18.9%)
CRI	3.7% (1.9% to 12.4%)	2.8% (1.5% to 8.0%)	1.8% (1.0% to 4.2%)	1.1% (0.59% to 3.1%)
CUB	3.6% (1.6% to 16.1%)	2.5% (1.4% to 10.6%)	1.6% (0.91% to 4.1%)	0.98% (0.49% to 2.1%)
CYP	0.67% (0.49% to 1.8%)	0.69% (0.47% to 1.8%)	0.70% (0.38% to 2.3%)	0.75% (0.28% to 3.9%)
CZE	3.1% (1.2% to 13.3%)	2.1% (1.0% to 8.5%)	1.1% (0.64% to 3.5%)	0.60% (0.33% to 1.2%)
DEU	2.3% (0.97% to 10.1%)	1.7% (0.87% to 6.6%)	0.93% (0.57% to 2.8%)	0.56% (0.32% to 1.5%)
DJI	48.8% (38.9% to 60.0%)	49.9% (37.6% to 67.6%)	52.8% (32.0% to 81.6%)	56.5% (25.9% to 91.2%)
DMA	7.5% (0.92% to 30.6%)	4.6% (0.75% to 19.9%)	1.7% (0.51% to 10.2%)	0.62% (0.25% to 4.3%)
DNK	1.2% (0.77% to 4.3%)	1.1% (0.69% to 2.7%)	0.93% (0.52% to 1.9%)	0.72% (0.30% to 2.4%)
DOM	16.5% (10.3% to 30.1%)	12.8% (8.8% to 22.5%)	8.5% (5.5% to 13.4%)	5.1% (3.0% to 10.4%)
DZA	12.5% (9.6% to 17.0%)	10.6% (8.1% to 14.8%)	8.1% (5.4% to 13.9%)	6.6% (3.5% to 14.6%)
ECU	14.3% (8.5% to 27.6%)	11.5% (7.3% to 20.5%)	7.9% (5.2% to 13.3%)	5.3% (3.1% to 13.3%)

ISO3 Code	2024 (95% UI)	2030 (95% UI)	2040 (95% UI)	2050 (95% UI)
EGY	5.4% (3.0% to 18.1%)	4.3% (2.5% to 12.4%)	3.0% (1.6% to 5.9%)	2.1% (0.97% to 4.4%)
ERI	18.8% (11.9% to 32.7%)	16.2% (10.5% to 25.5%)	11.9% (7.8% to 20.6%)	8.5% (4.6% to 20.5%)
ESP	2.6% (1.5% to 10.0%)	2.3% (1.4% to 6.3%)	1.7% (0.88% to 3.6%)	1.2% (0.54% to 3.9%)
EST	4.9% (3.0% to 13.8%)	4.1% (2.7% to 9.3%)	3.0% (1.8% to 5.3%)	1.9% (1.1% to 4.1%)
ETH	27.0% (21.6% to 35.7%)	23.0% (18.1% to 29.4%)	16.5% (12.3% to 25.5%)	11.4% (6.9% to 24.5%)
FIN	1.8% (0.84% to 8.5%)	1.4% (0.72% to 5.1%)	0.88% (0.51% to 1.9%)	0.54% (0.31% to 1.3%)
FJI	11.0% (7.0% to 21.7%)	10.1% (6.2% to 16.8%)	8.8% (4.5% to 15.5%)	7.5% (2.9% to 19.8%)
FRA	2.4% (1.2% to 10.2%)	1.9% (1.1% to 6.8%)	1.4% (0.79% to 3.2%)	0.93% (0.49% to 2.4%)
GAB	40.4% (30.8% to 52.6%)	40.5% (29.5% to 55.8%)	40.3% (25.2% to 70.8%)	40.2% (18.1% to 82.7%)
GBR	1.5% (1.1% to 3.7%)	1.6% (1.1% to 2.8%)	1.6% (0.83% to 3.3%)	1.6% (0.60% to 5.0%)
GEO	31.0% (20.5% to 43.8%)	24.3% (16.5% to 33.4%)	15.2% (10.8% to 22.2%)	8.9% (5.6% to 17.6%)
GHA	30.8% (21.9% to 41.7%)	27.7% (20.1% to 39.2%)	22.3% (14.7% to 46.5%)	17.8% (9.5% to 54.5%)
GIN	23.4% (15.9% to 37.2%)	20.0% (14.2% to 29.7%)	15.0% (10.3% to 27.6%)	10.6% (6.0% to 32.3%)
GMB	14.5% (10.8% to 22.3%)	13.2% (9.2% to 18.5%)	10.8% (7.0% to 18.8%)	8.8% (5.0% to 21.4%)
GNB	34.5% (25.9% to 45.4%)	33.7% (24.1% to 47.9%)	32.4% (18.7% to 61.0%)	32.9% (13.5% to 75.7%)
GRC	1.1% (0.64% to 5.4%)	0.94% (0.57% to 3.1%)	0.70% (0.40% to 1.5%)	0.48% (0.24% to 1.9%)
GRD	0.20% (0.14% to 0.35%)	0.19% (0.14% to 0.30%)	0.17% (0.10% to 0.34%)	0.16% (0.06% to 0.46%)
GTM	12.0% (8.6% to 22.9%)	11.4% (7.8% to 18.4%)	9.8% (5.5% to 19.8%)	8.3% (3.6% to 26.6%)
GUY	14.0% (9.5% to 26.2%)	12.9% (8.6% to 20.7%)	10.5% (6.6% to 20.5%)	8.6% (4.0% to 25.8%)
HKG	12.6% (8.4% to 25.8%)	11.3% (7.3% to 19.7%)	8.5% (5.2% to 16.5%)	6.4% (3.3% to 20.1%)
HND	13.7% (7.6% to 29.7%)	10.3% (6.5% to 20.8%)	6.4% (4.2% to 11.0%)	3.8% (2.2% to 8.6%)
HRV	7.1% (3.1% to 20.2%)	5.0% (2.5% to 13.7%)	2.8% (1.6% to 6.2%)	1.5% (0.83% to 3.1%)
HTI	25.5% (19.5% to 38.8%)	23.7% (17.0% to 33.0%)	19.8% (12.2% to 35.1%)	16.6% (7.6% to 44.3%)
HUN	5.3% (2.4% to 17.7%)	3.9% (2.0% to 11.6%)	2.3% (1.3% to 5.0%)	1.3% (0.74% to 2.4%)
IDN	46.5% (39.2% to 56.9%)	44.1% (35.1% to 57.6%)	40.0% (27.2% to 62.6%)	36.2% (18.9% to 68.0%)
IRL	1.9% (1.0% to 8.7%)	1.6% (0.91% to 5.9%)	1.1% (0.66% to 2.9%)	0.70% (0.39% to 2.2%)
IRN	4.0% (2.6% to 12.7%)	3.7% (2.4% to 8.9%)	3.1% (1.7% to 6.0%)	2.4% (1.0% to 7.4%)
IRQ	6.0% (4.3% to 10.7%)	6.1% (4.0% to 10.7%)	6.1% (3.3% to 18.3%)	6.7% (2.1% to 31.8%)
ISL	0.72% (0.41% to 3.6%)	0.61% (0.37% to 2.1%)	0.48% (0.27% to 1.0%)	0.33% (0.18% to 1.0%)
ISR	0.96% (0.64% to 3.1%)	0.84% (0.56% to 2.0%)	0.64% (0.40% to 1.1%)	0.45% (0.25% to 1.2%)
ITA	1.1% (0.67% to 3.9%)	1.0% (0.64% to 2.3%)	0.81% (0.51% to 1.5%)	0.59% (0.31% to 1.9%)
JAM	0.74% (0.51% to 3.2%)	0.70% (0.47% to 2.0%)	0.61% (0.36% to 1.2%)	0.51% (0.23% to 1.4%)
JOR	1.3% (0.69% to 6.5%)	1.1% (0.62% to 4.1%)	0.79% (0.43% to 1.8%)	0.55% (0.27% to 1.6%)
JPN	5.0% (2.6% to 16.7%)	3.9% (2.3% to 10.9%)	2.7% (1.6% to 5.3%)	1.7% (0.95% to 4.5%)
KAZ	13.0% (9.9% to 20.1%)	13.1% (9.2% to 20.9%)	12.4% (7.1% to 27.7%)	11.9% (4.9% to 37.7%)
KEN	18.8% (14.9% to 25.7%)	19.5% (14.8% to 29.6%)	20.5% (12.0% to 42.4%)	22.4% (8.8% to 59.8%)
KGZ	21.5% (16.5% to 34.6%)	19.8% (15.2% to 30.4%)	16.2% (10.7% to 31.0%)	12.6% (6.7% to 36.5%)
KHM	56.2% (46.9% to 65.2%)	50.3% (41.7% to 61.5%)	39.6% (28.8% to 57.6%)	29.6% (18.2% to 58.0%)
KNA	0.77% (0.52% to 1.5%)	0.85% (0.52% to 1.5%)	0.91% (0.46% to 2.5%)	0.97% (0.35% to 4.2%)
KOR	18.1% (13.9% to 24.7%)	14.0% (10.4% to 20.5%)	9.4% (6.2% to 18.6%)	6.3% (3.3% to 19.5%)
KWT	3.9% (2.9% to 5.7%)	3.4% (2.5% to 5.1%)	2.7% (1.6% to 4.8%)	2.2% (0.97% to 5.5%)
LAO	45.9% (37.1% to 56.5%)	40.2% (31.5% to 49.8%)	30.6% (21.8% to 47.4%)	22.1% (12.7% to 45.7%)
LBN	2.7% (1.5% to 8.4%)	2.4% (1.4% to 5.4%)	2.0% (1.1% to 4.0%)	1.6% (0.64% to 5.9%)
LBR	33.7% (24.9% to 46.3%)	34.7% (25.1% to 55.1%)	36.3% (21.5% to 75.2%)	39.5% (17.3% to 89.2%)
LBY	7.9% (5.5% to 12.3%)	6.5% (4.5% to 10.1%)	5.1% (3.1% to 10.6%)	4.1% (2.0% to 14.0%)
LCA	2.4% (1.2% to 9.8%)	1.8% (1.1% to 6.1%)	1.2% (0.72% to 2.6%)	0.82% (0.43% to 1.8%)
LKA	9.4% (6.8% to 19.1%)	9.3% (6.3% to 15.9%)	8.4% (4.8% to 17.8%)	7.6% (3.3% to 26.9%)
LTU	9.3% (6.6% to 16.7%)	8.9% (6.0% to 14.5%)	7.4% (4.4% to 14.0%)	6.0% (2.9% to 16.1%)
LUX	1.3% (0.81% to 4.3%)	1.1% (0.73% to 2.9%)	0.85% (0.52% to 1.7%)	0.59% (0.32% to 1.7%)

ISO3 Code	2024 (95% UI)	2030 (95% UI)	2040 (95% UI)	2050 (95% UI)
LVA	13.7% (7.5% to 28.2%)	10.5% (6.1% to 19.7%)	6.6% (4.1% to 10.3%)	3.9% (2.3% to 7.9%)
MAC	11.4% (8.0% to 23.2%)	10.8% (7.4% to 17.6%)	8.9% (5.4% to 19.6%)	7.2% (3.3% to 24.3%)
MAR	14.8% (10.3% to 25.3%)	13.6% (9.3% to 20.6%)	11.4% (7.0% to 23.0%)	9.6% (4.4% to 30.8%)
MDA	18.6% (13.6% to 25.5%)	19.6% (13.7% to 32.3%)	20.4% (11.2% to 45.3%)	21.6% (8.5% to 60.6%)
MDG	40.3% (29.9% to 52.3%)	35.6% (26.1% to 47.7%)	29.6% (17.8% to 52.6%)	23.8% (11.4% to 56.9%)
MEX	7.2% (3.2% to 18.7%)	5.2% (2.9% to 12.6%)	3.1% (1.9% to 6.3%)	1.9% (1.1% to 5.6%)
MKD	4.6% (2.6% to 16.0%)	3.8% (2.2% to 9.9%)	2.5% (1.5% to 5.0%)	1.6% (0.92% to 4.0%)
MLI	11.1% (7.5% to 20.4%)	10.1% (7.2% to 15.3%)	8.2% (5.5% to 16.9%)	6.7% (3.6% to 20.7%)
MLT	1.1% (0.74% to 1.9%)	1.2% (0.74% to 2.9%)	1.6% (0.78% to 6.8%)	2.2% (0.78% to 16.3%)
MMR	41.8% (33.1% to 51.5%)	37.4% (30.3% to 47.6%)	30.1% (20.7% to 46.3%)	23.7% (12.4% to 47.4%)
MNE	9.9% (4.5% to 22.3%)	6.9% (3.7% to 15.3%)	3.9% (2.2% to 7.8%)	2.1% (1.3% to 3.6%)
MNG	33.8% (23.9% to 45.9%)	27.2% (20.4% to 37.9%)	18.8% (13.1% to 31.2%)	12.7% (7.3% to 30.7%)
MOZ	44.6% (34.3% to 57.6%)	41.0% (31.5% to 54.6%)	36.6% (24.0% to 61.5%)	34.1% (16.3% to 67.9%)
MRT	30.6% (21.9% to 41.5%)	24.1% (18.5% to 33.3%)	15.9% (11.5% to 25.7%)	10.4% (6.3% to 22.3%)
MUS	4.5% (2.9% to 11.2%)	4.1% (2.7% to 8.3%)	3.4% (2.1% to 6.6%)	2.8% (1.4% to 8.3%)
MWI	27.4% (20.8% to 38.8%)	27.0% (18.6% to 40.7%)	26.4% (14.7% to 54.7%)	27.6% (9.8% to 69.6%)
MYS	10.0% (7.2% to 17.7%)	10.4% (6.7% to 16.6%)	10.9% (5.9% to 24.0%)	11.7% (4.2% to 35.8%)
NAM	45.1% (35.7% to 56.1%)	44.7% (33.7% to 63.0%)	44.4% (28.0% to 74.9%)	43.4% (19.8% to 84.4%)
NER	29.5% (19.5% to 44.4%)	23.4% (16.1% to 34.7%)	14.9% (9.8% to 22.1%)	9.0% (5.0% to 18.4%)
NGA	25.5% (20.1% to 34.3%)	25.0% (19.0% to 32.8%)	23.6% (15.6% to 41.9%)	23.2% (11.7% to 53.5%)
NIC	3.5% (2.3% to 10.3%)	3.4% (2.1% to 7.1%)	2.9% (1.5% to 7.5%)	2.6% (1.0% to 11.4%)
NLD	1.3% (0.74% to 7.7%)	1.1% (0.69% to 4.6%)	0.81% (0.52% to 2.0%)	0.56% (0.32% to 1.7%)
NOR	1.00% (0.61% to 2.0%)	0.99% (0.58% to 1.9%)	0.98% (0.44% to 3.0%)	0.97% (0.32% to 5.0%)
NPL	21.4% (15.3% to 33.9%)	19.9% (13.7% to 29.1%)	17.0% (9.5% to 32.1%)	14.7% (5.7% to 39.5%)
NZL	1.3% (0.79% to 3.7%)	1.2% (0.76% to 2.5%)	0.95% (0.57% to 2.0%)	0.71% (0.33% to 2.6%)
OMN	2.1% (1.3% to 7.9%)	1.8% (1.1% to 5.1%)	1.4% (0.81% to 3.2%)	0.97% (0.50% to 3.0%)
PAK	31.5% (24.5% to 40.8%)	28.7% (22.0% to 40.0%)	24.8% (15.9% to 41.7%)	20.8% (10.8% to 45.3%)
PAN	6.4% (4.5% to 11.9%)	6.3% (4.1% to 10.0%)	5.6% (3.2% to 12.1%)	5.1% (2.2% to 16.9%)
PER	22.6% (15.4% to 36.3%)	19.2% (13.7% to 29.5%)	14.3% (9.6% to 27.9%)	10.4% (6.3% to 31.7%)
POL	4.5% (2.7% to 15.5%)	3.7% (2.4% to 10.6%)	2.7% (1.6% to 5.2%)	1.8% (0.96% to 4.8%)
PRT	6.2% (3.4% to 16.5%)	4.8% (2.9% to 11.2%)	3.2% (1.9% to 6.1%)	2.0% (1.1% to 4.8%)
PRY	6.6% (4.5% to 15.4%)	6.1% (4.1% to 12.9%)	5.0% (2.9% to 12.3%)	4.1% (1.9% to 16.6%)
PSE	1.0% (0.61% to 3.7%)	0.89% (0.56% to 2.1%)	0.67% (0.37% to 1.5%)	0.48% (0.24% to 2.0%)
ROU	20.3% (13.2% to 34.5%)	16.2% (11.1% to 25.6%)	10.7% (7.2% to 17.4%)	6.7% (3.9% to 13.2%)
RUS	12.7% (9.6% to 20.8%)	12.1% (8.8% to 19.2%)	10.5% (6.5% to 23.2%)	8.9% (4.2% to 30.3%)
RWA	8.3% (6.1% to 17.0%)	8.3% (5.9% to 12.9%)	7.7% (4.7% to 13.3%)	7.2% (3.2% to 17.0%)
SAU	2.1% (1.3% to 4.8%)	2.0% (1.2% to 3.6%)	1.6% (0.95% to 4.1%)	1.4% (0.63% to 5.9%)
SDN	17.2% (11.6% to 28.8%)	15.7% (10.8% to 24.3%)	12.0% (8.1% to 23.1%)	9.8% (5.0% to 27.1%)
SEN	19.8% (15.0% to 30.0%)	18.6% (14.1% to 26.8%)	15.7% (10.5% to 30.3%)	13.5% (6.5% to 37.6%)
SGP	5.9% (3.5% to 14.9%)	5.5% (3.2% to 10.7%)	4.7% (2.6% to 9.4%)	4.0% (1.7% to 13.2%)
SLE	38.7% (30.4% to 50.7%)	36.7% (27.6% to 50.7%)	31.8% (20.7% to 59.3%)	28.6% (14.3% to 71.2%)
SLV	5.2% (3.3% to 13.3%)	5.1% (3.2% to 9.5%)	4.5% (2.4% to 10.5%)	4.0% (1.6% to 17.7%)
SOM	37.0% (28.9% to 47.6%)	34.5% (26.6% to 52.9%)	31.2% (18.9% to 60.2%)	28.8% (13.8% to 74.5%)
SRB	8.8% (4.3% to 24.2%)	6.5% (3.5% to 17.1%)	4.0% (2.4% to 8.9%)	2.3% (1.4% to 7.3%)
SSD	21.7% (16.0% to 32.2%)	21.5% (15.3% to 33.6%)	23.0% (12.1% to 48.6%)	24.8% (7.6% to 66.6%)
SUR	12.9% (7.0% to 25.1%)	9.6% (5.9% to 18.1%)	5.7% (3.8% to 10.1%)	3.3% (2.0% to 5.7%)
SVK	4.3% (1.6% to 14.6%)	3.0% (1.4% to 9.6%)	1.5% (0.86% to 4.2%)	0.79% (0.44% to 1.6%)
SVN	4.9% (1.8% to 15.6%)	3.2% (1.5% to 10.8%)	1.6% (0.97% to 5.0%)	0.85% (0.50% to 2.2%)
SWE	0.81% (0.57% to 2.3%)	0.85% (0.55% to 1.8%)	0.89% (0.46% to 2.9%)	0.95% (0.32% to 5.6%)

ISO3 Code	2024 (95% UI)	2030 (95% UI)	2040 (95% UI)	2050 (95% UI)
SWZ	36.7% (28.1% to 48.7%)	39.9% (27.2% to 59.5%)	45.6% (24.6% to 77.9%)	52.9% (20.6% to 91.0%)
SYC	3.2% (2.1% to 9.4%)	3.0% (2.0% to 5.9%)	2.5% (1.5% to 6.1%)	2.1% (1.1% to 8.7%)
SYR	5.2% (3.1% to 10.1%)	3.9% (2.5% to 6.8%)	2.4% (1.6% to 4.4%)	1.4% (0.76% to 4.2%)
TCD	16.8% (12.3% to 26.0%)	17.0% (11.7% to 26.6%)	16.1% (9.1% to 35.2%)	15.7% (6.2% to 47.8%)
TGO	6.3% (4.5% to 11.7%)	6.5% (4.3% to 11.2%)	7.2% (3.4% to 16.4%)	8.2% (2.6% to 28.2%)
THA	24.5% (18.7% to 32.2%)	22.6% (16.6% to 29.8%)	19.1% (12.7% to 31.6%)	16.6% (8.6% to 36.4%)
TJK	20.3% (14.5% to 33.2%)	18.2% (13.2% to 26.0%)	13.5% (9.1% to 21.7%)	9.5% (5.3% to 19.9%)
TKM	20.7% (15.0% to 35.5%)	17.6% (12.7% to 26.8%)	12.2% (8.6% to 20.4%)	7.7% (4.7% to 16.5%)
TTO	1.8% (1.2% to 3.3%)	2.0% (1.3% to 3.7%)	2.3% (1.1% to 6.1%)	2.7% (0.86% to 10.3%)
TUN	3.5% (2.6% to 8.1%)	3.7% (2.4% to 6.5%)	4.0% (2.0% to 9.6%)	4.3% (1.5% to 15.3%)
TUR	4.0% (2.4% to 14.7%)	3.3% (2.1% to 10.3%)	2.3% (1.3% to 5.2%)	1.4% (0.79% to 3.4%)
TZA	37.8% (30.2% to 48.8%)	37.5% (27.2% to 53.5%)	36.8% (21.2% to 66.3%)	36.3% (14.8% to 79.4%)
UGA	18.6% (12.0% to 30.4%)	15.5% (10.5% to 23.8%)	10.8% (7.3% to 18.8%)	7.4% (4.3% to 17.3%)
UKR	11.5% (7.8% to 19.5%)	11.3% (7.1% to 16.8%)	10.5% (5.3% to 21.9%)	9.4% (3.6% to 28.5%)
URY	3.1% (1.9% to 7.0%)	3.2% (1.8% to 5.1%)	2.9% (1.5% to 7.2%)	2.8% (1.1% to 11.0%)
USA	1.3% (0.55% to 7.7%)	0.90% (0.50% to 4.5%)	0.54% (0.32% to 1.5%)	0.32% (0.18% to 0.71%)
UZB	11.9% (9.1% to 18.8%)	12.1% (8.9% to 19.3%)	12.1% (7.1% to 25.8%)	12.0% (4.8% to 34.4%)
VCT	5.2% (3.1% to 13.7%)	4.4% (2.9% to 9.6%)	3.2% (2.0% to 6.4%)	2.3% (1.2% to 7.4%)
VEN	3.4% (2.2% to 10.0%)	3.2% (2.0% to 6.7%)	2.6% (1.5% to 5.6%)	2.2% (0.95% to 7.3%)
YEM	17.2% (9.7% to 31.8%)	13.0% (8.2% to 23.7%)	7.8% (5.0% to 13.7%)	4.5% (2.5% to 8.2%)
ZAF	39.8% (32.7% to 51.0%)	41.5% (30.9% to 59.8%)	45.6% (26.0% to 76.4%)	50.8% (20.4% to 88.8%)
ZMB	39.3% (29.9% to 52.1%)	34.7% (26.9% to 47.8%)	27.5% (20.2% to 49.9%)	21.9% (11.9% to 55.7%)
ZWE	24.6% (17.8% to 34.0%)	22.8% (16.2% to 32.3%)	19.4% (12.2% to 35.8%)	16.6% (7.7% to 41.8%)

Appendix Table 6. Projected prevalence of recently acquired Mtb immunoreactivity among immigrants under the status quo scenario for selected years (2024, 2030, 2040, and 2050), China, India, Philippines, Viet Nam

Country	2024 (95% UI)	2030 (95% UI)	2040 (95% UI)	2050 (95% UI)
Primary analysis				
China	0.32% (0.14% to 0.74%)	0.26% (0.10% to 0.76%)	0.19% (0.05% to 0.78%)	0.14% (0.03% to 0.78%)
India	0.71% (0.35% to 1.4%)	0.57% (0.25% to 1.4%)	0.42% (0.14% to 1.3%)	0.30% (0.07% to 1.4%)
Philippines	1.1% (0.56% to 2.3%)	1.0% (0.43% to 2.3%)	0.83% (0.25% to 2.5%)	0.67% (0.14% to 2.6%)
Viet Nam	0.55% (0.21% to 1.7%)	0.49% (0.14% to 1.8%)	0.37% (0.06% to 1.9%)	0.28% (0.03% to 2.1%)
Sensitivity analysis allowing for reversion of immunoreactivity				
China	0.93% (0.41% to 2.1%)	0.76% (0.28% to 2.2%)	0.56% (0.15% to 2.3%)	0.40% (0.08% to 2.3%)
India	2.1% (1.0% to 4.0%)	1.7% (0.72% to 4.0%)	1.2% (0.41% to 3.9%)	0.86% (0.21% to 4.1%)
Philippines	3.3% (1.6% to 6.6%)	3.0% (1.2% to 6.8%)	2.4% (0.73% to 7.2%)	1.9% (0.41% to 7.4%)
Viet Nam	1.6% (0.60% to 5.0%)	1.4% (0.40% to 5.2%)	1.1% (0.19% to 5.6%)	0.80% (0.08% to 6.0%)

Appendix Table 7. Absolute difference (in percentage points) in overall prevalence of Mtb immunoreactivity compared to status quo (2024) under additional ARI reduction scenarios, China, India, Philippines, Viet Nam

	Additional 1% reduction in ARI scenario			Additional 3% reduction in ARI scenario			Additional 5% reduction in ARI scenario		
Country	2030 (95% UI)	2040 (95% UI)	2050 (95% UI)	2030 (95% UI)	2040 (95% UI)	2050 (95% UI)	2030 (95% UI)	2040 (95% UI)	2050 (95% UI)
China	−0.05% (−0.13% to −0.02%)	−0.18% (−0.60% to −0.06%)	−0.33% (−1.3% to −0.09%)	−0.14% (−0.36% to −0.05%)	−0.49% (−1.6% to −0.17%)	−0.84% (−3.4% to −0.23%)	−0.21% (−0.56% to −0.08%)	−0.73% (−2.4% to −0.25%)	−1.2% (−4.8% to −0.33%)
India	−0.11% (−0.23% to −0.05%)	−0.41% (−1.1% to −0.17%)	−0.74% (−2.2% to −0.25%)	−0.30% (−0.64% to −0.14%)	−1.1% (−2.9% to −0.45%)	−1.9% (−5.8% to −0.65%)	−0.47% (−1.0% to −0.22%)	−1.7% (−4.4% to −0.67%)	−2.7% (−8.3% to −0.94%)
Philippines	−0.18% (−0.38% to −0.08%)	−0.77% (−1.8% to −0.30%)	−1.5% (−3.9% to −0.45%)	−0.52% (−1.1% to −0.24%)	−2.1% (−4.9% to −0.80%)	−3.8% (−10.4% to −1.1%)	−0.81% (−1.7% to −0.37%)	−3.1% (−7.4% to −1.2%)	−5.5% (−15.4% to −1.7%)
Viet Nam	−0.09% (−0.29% to −0.03%)	−0.36% (−1.4% to −0.09%)	−0.65% (−3.0% to −0.12%)	−0.25% (−0.83% to −0.08%)	−0.96% (−3.7% to −0.24%)	−1.7% (−7.8% to −0.32%)	−0.40% (−1.3% to −0.12%)	−1.4% (−5.6% to −0.36%)	−2.4% (−11.3% to −0.46%)

Appendix Table 8. Absolute difference (in percentage points) in overall prevalence of Mtb immunoreactivity compared to status quo (2024) under additional ARI reduction scenarios, all other countries

ISO3	Additional 1% reduction in ARI scenario			Additional 3% reduction in ARI scenario			Additional 5% reduction in ARI scenario		
Code	2030 (95% UI)	2040 (95% UI)	2050 (95% UI)	2030 (95% UI)	2040 (95% UI)	2050 (95% UI)	2030 (95% UI)	2040 (95% UI)	2050 (95% UI)
AFG	−0.15% (−0.35% to −0.06%)	−0.67% (−1.8% to −0.22%)	−1.3% (−4.1% to −0.37%)	−0.44% (−0.99% to −0.17%)	−1.8% (−4.8% to −0.58%)	−3.4% (−11.0% to −0.94%)	−0.69% (−1.6% to −0.27%)	−2.7% (−7.3% to −0.87%)	−4.9% (−16.1% to −1.4%)
AGO	−0.29% (−0.62% to −0.11%)	−1.4% (−2.7% to −0.42%)	−3.0% (−5.3% to −0.74%)	−0.83% (−1.8% to −0.31%)	−3.8% (−7.9% to −1.1%)	−7.9% (−15.2% to −1.9%)	−1.3% (−2.8% to −0.49%)	−5.8% (−12.4% to −1.7%)	−11.4% (−24.0% to −2.7%)
ALB	−0.02% (−0.05% to −0.01%)	−0.07% (−0.29% to −0.02%)	−0.13% (−0.78% to −0.02%)	−0.04% (−0.15% to −0.01%)	−0.18% (−0.77% to −0.04%)	−0.32% (−2.0% to −0.06%)	−0.07% (−0.23% to −0.02%)	−0.27% (−1.1% to −0.06%)	−0.46% (−2.8% to −0.08%)
ARE	−0.00% (−0.00% to −0.00%)	−0.00% (−0.01% to −0.00%)	−0.00% (−0.01% to −0.00%)	−0.00% (−0.01% to −0.00%)	−0.01% (−0.03% to −0.00%)	−0.01% (−0.04% to −0.00%)	−0.01% (−0.01% to −0.00%)	−0.01% (−0.04% to −0.00%)	−0.01% (−0.05% to −0.00%)
ARG	−0.02% (−0.05% to −0.01%)	−0.06% (−0.25% to −0.02%)	−0.11% (−0.61% to −0.02%)	−0.05% (−0.14% to −0.02%)	−0.16% (−0.68% to −0.05%)	−0.27% (−1.5% to −0.06%)	−0.07% (−0.21% to −0.03%)	−0.24% (−1.0% to −0.07%)	−0.38% (−2.2% to −0.09%)
ARM	−0.06% (−0.18% to −0.01%)	−0.27% (−0.90% to −0.05%)	−0.55% (−2.4% to −0.09%)	−0.16% (−0.50% to −0.04%)	−0.72% (−2.4% to −0.14%)	−1.4% (−6.1% to −0.23%)	−0.26% (−0.79% to −0.06%)	−1.1% (−3.6% to −0.21%)	−2.0% (−8.8% to −0.33%)
ATG	−0.01% (−0.02% to −0.00%)	−0.03% (−0.16% to −0.01%)	−0.06% (−0.52% to −0.01%)	−0.02% (−0.06% to −0.01%)	−0.08% (−0.42% to −0.02%)	−0.16% (−1.3% to −0.03%)	−0.03% (−0.10% to −0.01%)	−0.11% (−0.62% to −0.03%)	−0.23% (−1.8% to −0.05%)
AUS	−0.01% (−0.03% to −0.00%)	−0.03% (−0.17% to −0.01%)	−0.07% (−0.59% to −0.01%)	−0.02% (−0.07% to −0.01%)	−0.08% (−0.47% to −0.02%)	−0.17% (−1.5% to −0.03%)	−0.03% (−0.11% to −0.01%)	−0.13% (−0.69% to −0.03%)	−0.24% (−2.1% to −0.04%)
AUT	−0.01% (−0.02% to −0.00%)	−0.02% (−0.07% to −0.00%)	−0.03% (−0.16% to −0.01%)	−0.02% (−0.04% to −0.00%)	−0.05% (−0.19% to −0.01%)	−0.09% (−0.39% to −0.02%)	−0.03% (−0.07% to −0.01%)	−0.08% (−0.28% to −0.02%)	−0.12% (−0.56% to −0.02%)
AZE	−0.02% (−0.05% to −0.01%)	−0.05% (−0.17% to −0.02%)	−0.06% (−0.25% to −0.02%)	−0.05% (−0.15% to −0.02%)	−0.14% (−0.46% to −0.04%)	−0.16% (−0.66% to −0.05%)	−0.08% (−0.24% to −0.03%)	−0.20% (−0.69% to −0.06%)	−0.23% (−0.96% to −0.07%)
BDI	−0.09% (−0.25% to −0.04%)	−0.36% (−1.2% to −0.12%)	−0.63% (−2.6% to −0.18%)	−0.26% (−0.72% to −0.10%)	−0.96% (−3.2% to −0.32%)	−1.6% (−6.6% to −0.46%)	−0.41% (−1.1% to −0.16%)	−1.4% (−4.8% to −0.48%)	−2.3% (−9.6% to −0.66%)
BEL	−0.01% (−0.02% to −0.00%)	−0.03% (−0.11% to −0.01%)	−0.06% (−0.28% to −0.02%)	−0.02% (−0.06% to −0.01%)	−0.08% (−0.29% to −0.03%)	−0.14% (−0.70% to −0.04%)	−0.04% (−0.10% to −0.02%)	−0.12% (−0.44% to −0.05%)	−0.20% (−0.98% to −0.06%)
BEN	−0.04% (−0.11% to −0.02%)	−0.18% (−0.54% to −0.06%)	−0.32% (−1.2% to −0.09%)	−0.12% (−0.31% to −0.05%)	−0.47% (−1.5% to −0.16%)	−0.80% (−3.1% to −0.22%)	−0.19% (−0.49% to −0.07%)	−0.71% (−2.2% to −0.23%)	−1.1% (−4.3% to −0.32%)

ISO3 Code	Additional 1% reduction in ARI scenario			Additional 3% reduction in ARI scenario			Additional 5% reduction in ARI scenario		
	2030 (95% UI)	2040 (95% UI)	2050 (95% UI)	2030 (95% UI)	2040 (95% UI)	2050 (95% UI)	2030 (95% UI)	2040 (95% UI)	2050 (95% UI)
BFA	−0.07% (−0.19% to −0.02%)	−0.30% (−0.98% to −0.09%)	−0.63% (−2.6% to −0.15%)	−0.19% (−0.53% to −0.07%)	−0.79% (−2.6% to −0.24%)	−1.6% (−6.7% to −0.37%)	−0.30% (−0.84% to −0.11%)	−1.2% (−4.0% to −0.35%)	−2.3% (−9.5% to −0.53%)
BGD	−0.25% (−0.55% to −0.11%)	−1.1% (−2.6% to −0.42%)	−2.4% (−5.1% to −0.75%)	−0.70% (−1.6% to −0.31%)	−3.1% (−7.3% to −1.1%)	−6.3% (−15.0% to −1.9%)	−1.1% (−2.5% to −0.48%)	−4.7% (−11.2% to −1.7%)	−9.2% (−23.6% to −2.8%)
BGR	−0.02% (−0.08% to −0.01%)	−0.08% (−0.38% to −0.02%)	−0.13% (−0.86% to −0.02%)	−0.06% (−0.22% to −0.02%)	−0.21% (−1.0% to −0.05%)	−0.34% (−2.2% to −0.06%)	−0.10% (−0.35% to −0.03%)	−0.32% (−1.5% to −0.07%)	−0.49% (−3.1% to −0.09%)
BHR	−0.01% (−0.03% to −0.00%)	−0.05% (−0.18% to −0.01%)	−0.09% (−0.47% to −0.02%)	−0.04% (−0.09% to −0.01%)	−0.13% (−0.49% to −0.04%)	−0.22% (−1.2% to −0.05%)	−0.06% (−0.15% to −0.02%)	−0.19% (−0.73% to −0.06%)	−0.32% (−1.7% to −0.08%)
BHS	−0.01% (−0.02% to −0.00%)	−0.03% (−0.10% to −0.01%)	−0.04% (−0.21% to −0.01%)	−0.02% (−0.06% to −0.01%)	−0.07% (−0.27% to −0.02%)	−0.11% (−0.54% to −0.03%)	−0.03% (−0.09% to −0.01%)	−0.10% (−0.40% to −0.03%)	−0.15% (−0.77% to −0.04%)
BIH	−0.04% (−0.13% to −0.01%)	−0.15% (−0.66% to −0.04%)	−0.26% (−1.5% to −0.05%)	−0.11% (−0.38% to −0.03%)	−0.40% (−1.8% to −0.10%)	−0.66% (−3.8% to −0.14%)	−0.18% (−0.60% to −0.05%)	−0.59% (−2.6% to −0.15%)	−0.94% (−5.4% to −0.20%)
BLR	−0.07% (−0.23% to −0.02%)	−0.30% (−1.3% to −0.07%)	−0.60% (−3.3% to −0.11%)	−0.20% (−0.64% to −0.06%)	−0.81% (−3.4% to −0.18%)	−1.5% (−8.5% to −0.29%)	−0.31% (−1.0% to −0.09%)	−1.2% (−5.1% to −0.27%)	−2.2% (−12.2% to −0.42%)
BLZ	−0.03% (−0.09% to −0.01%)	−0.11% (−0.49% to −0.03%)	−0.21% (−1.3% to −0.05%)	−0.08% (−0.25% to −0.03%)	−0.30% (−1.3% to −0.09%)	−0.54% (−3.3% to −0.12%)	−0.12% (−0.39% to −0.04%)	−0.44% (−1.9% to −0.13%)	−0.76% (−4.7% to −0.18%)
BOL	−0.09% (−0.26% to −0.03%)	−0.38% (−1.3% to −0.12%)	−0.72% (−3.0% to −0.19%)	−0.26% (−0.75% to −0.10%)	−1.0% (−3.5% to −0.33%)	−1.8% (−7.8% to −0.49%)	−0.41% (−1.2% to −0.15%)	−1.5% (−5.2% to −0.50%)	−2.6% (−11.3% to −0.71%)
BRA	−0.03% (−0.08% to −0.01%)	−0.13% (−0.44% to −0.03%)	−0.24% (−1.2% to −0.04%)	−0.09% (−0.24% to −0.03%)	−0.34% (−1.2% to −0.08%)	−0.61% (−3.0% to −0.11%)	−0.14% (−0.37% to −0.04%)	−0.51% (−1.7% to −0.12%)	−0.87% (−4.2% to −0.16%)
BRB	−0.00% (−0.00% to −0.00%)	−0.00% (−0.00% to −0.00%)	−0.00% (−0.01% to −0.00%)	−0.00% (−0.00% to −0.00%)	−0.00% (−0.01% to −0.00%)	−0.00% (−0.02% to −0.00%)	−0.00% (−0.01% to −0.00%)	−0.01% (−0.02% to −0.00%)	−0.01% (−0.03% to −0.00%)
BRN	−0.06% (−0.21% to −0.02%)	−0.26% (−1.2% to −0.06%)	−0.56% (−3.3% to −0.09%)	−0.17% (−0.59% to −0.05%)	−0.70% (−3.3% to −0.16%)	−1.4% (−8.6% to −0.23%)	−0.27% (−0.94% to −0.08%)	−1.1% (−5.0% to −0.24%)	−2.0% (−12.5% to −0.33%)
BTN	−0.04% (−0.14% to −0.02%)	−0.14% (−0.60% to −0.05%)	−0.22% (−1.1% to −0.06%)	−0.12% (−0.40% to −0.04%)	−0.39% (−1.6% to −0.12%)	−0.56% (−2.9% to −0.16%)	−0.19% (−0.62% to −0.07%)	−0.58% (−2.4% to −0.19%)	−0.81% (−4.2% to −0.23%)
BWA	−0.14% (−0.34% to −0.06%)	−0.58% (−1.7% to −0.21%)	−1.1% (−3.9% to −0.31%)	−0.40% (−0.98% to −0.18%)	−1.6% (−4.7% to −0.55%)	−2.7% (−10.5% to −0.80%)	−0.63% (−1.5% to −0.28%)	−2.4% (−7.2% to −0.83%)	−3.9% (−15.4% to −1.2%)
CAF	−0.12% (−0.29% to −0.04%)	−0.46% (−1.3% to −0.12%)	−0.79% (−2.7% to −0.18%)	−0.34% (−0.84% to −0.11%)	−1.2% (−3.6% to −0.33%)	−2.0% (−7.1% to −0.46%)	−0.53% (−1.3% to −0.18%)	−1.9% (−5.4% to −0.50%)	−2.9% (−10.3% to −0.66%)
CHE	−0.00% (−0.01% to −0.00%)	−0.02% (−0.07% to −0.01%)	−0.03% (−0.18% to −0.01%)	−0.01% (−0.04% to −0.01%)	−0.05% (−0.20% to −0.01%)	−0.08% (−0.44% to −0.02%)	−0.02% (−0.06% to −0.01%)	−0.07% (−0.29% to −0.02%)	−0.11% (−0.62% to −0.03%)
CHL	−0.01% (−0.03% to −0.00%)	−0.04% (−0.17% to −0.01%)	−0.06% (−0.41% to −0.01%)	−0.03% (−0.08% to −0.01%)	−0.10% (−0.44% to −0.03%)	−0.16% (−1.0% to −0.03%)	−0.04% (−0.13% to −0.02%)	−0.14% (−0.66% to −0.04%)	−0.23% (−1.5% to −0.05%)
CIV	−0.10% (−0.28% to −0.03%)	−0.40% (−1.3% to −0.11%)	−0.71% (−2.9% to −0.15%)	−0.29% (−0.81% to −0.10%)	−1.1% (−3.6% to −0.28%)	−1.8% (−7.6% to −0.39%)	−0.45% (−1.3% to −0.15%)	−1.6% (−5.4% to −0.43%)	−2.6% (−11.0% to −0.57%)
CMR	−0.19% (−0.43% to −0.08%)	−0.80% (−2.2% to −0.26%)	−1.6% (−5.0% to −0.43%)	−0.53% (−1.2% to −0.21%)	−2.2% (−6.0% to −0.70%)	−4.1% (−13.6% to −1.1%)	−0.84% (−1.9% to −0.34%)	−3.2% (−9.1% to −1.0%)	−5.8% (−20.4% to −1.6%)
COD	−0.29% (−0.58% to −0.11%)	−1.4% (−2.6% to −0.45%)	−3.1% (−5.2% to −0.76%)	−0.84% (−1.7% to −0.32%)	−3.8% (−7.5% to −1.2%)	−8.0% (−14.9% to −2.0%)	−1.3% (−2.6% to −0.50%)	−5.8% (−11.8% to −1.8%)	−11.6% (−23.3% to −2.8%)
COG	−0.36% (−0.80% to −0.14%)	−1.8% (−3.0% to −0.57%)	−3.9% (−5.6% to −1.1%)	−1.0% (−2.3% to −0.39%)	−4.8% (−8.7% to −1.5%)	−10.4% (−16.2% to −2.7%)	−1.6% (−3.7% to −0.62%)	−7.3% (−13.8% to −2.3%)	−15.5% (−25.4% to −3.9%)
COL	−0.02% (−0.07% to −0.01%)	−0.09% (−0.35% to −0.02%)	−0.16% (−0.85% to −0.03%)	−0.07% (−0.19% to −0.02%)	−0.25% (−0.93% to −0.06%)	−0.42% (−2.2% to −0.08%)	−0.11% (−0.30% to −0.04%)	−0.37% (−1.4% to −0.09%)	−0.59% (−3.0% to −0.11%)
COM	−0.05% (−0.15% to −0.02%)	−0.20% (−0.89% to −0.06%)	−0.40% (−2.5% to −0.10%)	−0.13% (−0.42% to −0.05%)	−0.55% (−2.4% to −0.16%)	−1.0% (−6.4% to −0.26%)	−0.21% (−0.66% to −0.07%)	−0.82% (−3.6% to −0.24%)	−1.4% (−9.1% to −0.37%)
CRI	−0.01% (−0.03% to −0.00%)	−0.03% (−0.15% to −0.01%)	−0.05% (−0.38% to −0.01%)	−0.02% (−0.08% to −0.01%)	−0.07% (−0.40% to −0.02%)	−0.11% (−0.95% to −0.03%)	−0.03% (−0.12% to −0.01%)	−0.11% (−0.60% to −0.03%)	−0.16% (−1.3% to −0.04%)

ISO3 Code	Additional 1% reduction in ARI scenario			Additional 3% reduction in ARI scenario			Additional 5% reduction in ARI scenario		
	2030 (95% UI)	2040 (95% UI)	2050 (95% UI)	2030 (95% UI)	2040 (95% UI)	2050 (95% UI)	2030 (95% UI)	2040 (95% UI)	2050 (95% UI)
CUB	−0.01% (−0.02% to −0.00%)	−0.03% (−0.09% to −0.01%)	−0.05% (−0.20% to −0.01%)	−0.02% (−0.05% to −0.01%)	−0.07% (−0.24% to −0.02%)	−0.11% (−0.51% to −0.03%)	−0.03% (−0.08% to −0.01%)	−0.10% (−0.36% to −0.03%)	−0.16% (−0.72% to −0.04%)
CYP	−0.01% (−0.03% to −0.00%)	−0.04% (−0.18% to −0.01%)	−0.09% (−0.58% to −0.01%)	−0.02% (−0.08% to −0.01%)	−0.09% (−0.49% to −0.02%)	−0.21% (−1.5% to −0.04%)	−0.03% (−0.12% to −0.01%)	−0.14% (−0.73% to −0.03%)	−0.30% (−2.1% to −0.05%)
CZE	−0.00% (−0.01% to −0.00%)	−0.01% (−0.03% to −0.00%)	−0.01% (−0.06% to −0.00%)	−0.01% (−0.02% to −0.00%)	−0.02% (−0.09% to −0.01%)	−0.04% (−0.15% to −0.01%)	−0.01% (−0.04% to −0.01%)	−0.04% (−0.13% to −0.01%)	−0.05% (−0.21% to −0.01%)
DEU	−0.00% (−0.01% to −0.00%)	−0.01% (−0.06% to −0.00%)	−0.02% (−0.13% to −0.01%)	−0.01% (−0.03% to −0.00%)	−0.04% (−0.15% to −0.01%)	−0.06% (−0.33% to −0.02%)	−0.02% (−0.05% to −0.01%)	−0.05% (−0.22% to −0.02%)	−0.08% (−0.46% to −0.02%)
DJI	−0.42% (−0.75% to −0.18%)	−2.0% (−2.7% to −0.77%)	−4.1% (−5.1% to −1.4%)	−1.2% (−2.2% to −0.52%)	−5.5% (−7.9% to −2.1%)	−11.4% (−14.8% to −3.7%)	−1.9% (−3.5% to −0.82%)	−8.4% (−12.7% to −3.1%)	−17.4% (−23.5% to −5.6%)
DMA	−0.00% (−0.00% to −0.00%)	−0.00% (−0.02% to −0.00%)	−0.00% (−0.04% to −0.00%)	−0.00% (−0.01% to −0.00%)	−0.01% (−0.05% to −0.00%)	−0.01% (−0.09% to −0.00%)	−0.00% (−0.02% to −0.00%)	−0.01% (−0.07% to −0.00%)	−0.01% (−0.13% to −0.00%)
DNK	−0.01% (−0.02% to −0.00%)	−0.03% (−0.12% to −0.01%)	−0.05% (−0.32% to −0.01%)	−0.02% (−0.06% to −0.01%)	−0.08% (−0.32% to −0.02%)	−0.14% (−0.80% to −0.02%)	−0.03% (−0.10% to −0.01%)	−0.11% (−0.48% to −0.02%)	−0.19% (−1.1% to −0.03%)
DOM	−0.03% (−0.10% to −0.01%)	−0.12% (−0.46% to −0.04%)	−0.20% (−1.0% to −0.06%)	−0.10% (−0.27% to −0.03%)	−0.32% (−1.2% to −0.11%)	−0.51% (−2.6% to −0.15%)	−0.15% (−0.43% to −0.05%)	−0.49% (−1.8% to −0.16%)	−0.74% (−3.6% to −0.21%)
DZA	−0.06% (−0.14% to −0.03%)	−0.25% (−0.68% to −0.09%)	−0.49% (−1.6% to −0.14%)	−0.17% (−0.39% to −0.07%)	−0.68% (−1.8% to −0.23%)	−1.2% (−4.0% to −0.35%)	−0.27% (−0.61% to −0.11%)	−1.0% (−2.7% to −0.35%)	−1.8% (−5.7% to −0.50%)
ECU	−0.04% (−0.13% to −0.01%)	−0.16% (−0.62% to −0.04%)	−0.27% (−1.4% to −0.06%)	−0.12% (−0.36% to −0.04%)	−0.42% (−1.7% to −0.11%)	−0.70% (−3.6% to −0.15%)	−0.18% (−0.57% to −0.06%)	−0.62% (−2.5% to −0.16%)	−1.00% (−5.1% to −0.21%)
EGY	−0.02% (−0.05% to −0.00%)	−0.06% (−0.24% to −0.01%)	−0.12% (−0.53% to −0.02%)	−0.04% (−0.14% to −0.01%)	−0.17% (−0.63% to −0.04%)	−0.31% (−1.3% to −0.05%)	−0.07% (−0.22% to −0.02%)	−0.26% (−0.94% to −0.06%)	−0.45% (−1.9% to −0.08%)
ERI	−0.07% (−0.19% to −0.02%)	−0.28% (−0.92% to −0.08%)	−0.51% (−2.1% to −0.13%)	−0.20% (−0.54% to −0.07%)	−0.74% (−2.5% to −0.22%)	−1.3% (−5.4% to −0.34%)	−0.31% (−0.85% to −0.11%)	−1.1% (−3.7% to −0.32%)	−1.9% (−7.8% to −0.48%)
ESP	−0.01% (−0.04% to −0.00%)	−0.04% (−0.19% to −0.01%)	−0.07% (−0.48% to −0.01%)	−0.03% (−0.10% to −0.01%)	−0.10% (−0.50% to −0.02%)	−0.17% (−1.2% to −0.03%)	−0.04% (−0.16% to −0.01%)	−0.15% (−0.74% to −0.04%)	−0.25% (−1.7% to −0.05%)
EST	−0.02% (−0.05% to −0.01%)	−0.05% (−0.20% to −0.02%)	−0.08% (−0.42% to −0.02%)	−0.04% (−0.13% to −0.02%)	−0.14% (−0.53% to −0.04%)	−0.21% (−1.1% to −0.05%)	−0.07% (−0.20% to −0.03%)	−0.21% (−0.79% to −0.06%)	−0.30% (−1.5% to −0.08%)
ETH	−0.09% (−0.22% to −0.04%)	−0.35% (−1.0% to −0.11%)	−0.63% (−2.2% to −0.16%)	−0.25% (−0.62% to −0.10%)	−0.94% (−2.8% to −0.30%)	−1.6% (−5.7% to −0.42%)	−0.40% (−0.97% to −0.16%)	−1.4% (−4.2% to −0.45%)	−2.3% (−8.2% to −0.62%)
FIN	−0.00% (−0.01% to −0.00%)	−0.02% (−0.06% to −0.00%)	−0.03% (−0.13% to −0.01%)	−0.01% (−0.04% to −0.00%)	−0.04% (−0.16% to −0.01%)	−0.06% (−0.33% to −0.02%)	−0.02% (−0.06% to −0.01%)	−0.06% (−0.24% to −0.02%)	−0.09% (−0.47% to −0.02%)
FJI	−0.07% (−0.17% to −0.02%)	−0.30% (−0.96% to −0.06%)	−0.62% (−2.4% to −0.09%)	−0.19% (−0.50% to −0.05%)	−0.80% (−2.6% to −0.15%)	−1.6% (−6.2% to −0.22%)	−0.29% (−0.78% to −0.08%)	−1.2% (−3.9% to −0.22%)	−2.2% (−8.9% to −0.32%)
FRA	−0.01% (−0.02% to −0.00%)	−0.03% (−0.12% to −0.01%)	−0.06% (−0.27% to −0.02%)	−0.02% (−0.06% to −0.01%)	−0.08% (−0.31% to −0.03%)	−0.14% (−0.67% to −0.04%)	−0.04% (−0.10% to −0.01%)	−0.12% (−0.46% to −0.04%)	−0.20% (−0.95% to −0.06%)
GAB	−0.33% (−0.68% to −0.12%)	−1.5% (−2.7% to −0.47%)	−3.1% (−5.1% to −0.86%)	−0.93% (−2.0% to −0.34%)	−4.1% (−7.8% to −1.3%)	−8.6% (−14.8% to −2.2%)	−1.5% (−3.1% to −0.53%)	−6.1% (−12.5% to −1.9%)	−12.5% (−23.3% to −3.1%)
GBR	−0.02% (−0.04% to −0.01%)	−0.08% (−0.23% to −0.02%)	−0.17% (−0.72% to −0.03%)	−0.04% (−0.11% to −0.01%)	−0.20% (−0.63% to −0.05%)	−0.43% (−1.8% to −0.08%)	−0.07% (−0.18% to −0.02%)	−0.30% (−0.93% to −0.08%)	−0.60% (−2.5% to −0.12%)
GEO	−0.05% (−0.15% to −0.01%)	−0.18% (−0.64% to −0.03%)	−0.28% (−1.2% to −0.04%)	−0.15% (−0.44% to −0.04%)	−0.47% (−1.7% to −0.09%)	−0.72% (−3.2% to −0.11%)	−0.23% (−0.69% to −0.06%)	−0.71% (−2.6% to −0.14%)	−1.0% (−4.5% to −0.17%)
GHA	−0.15% (−0.48% to −0.05%)	−0.62% (−2.2% to −0.19%)	−1.2% (−4.7% to −0.28%)	−0.42% (−1.4% to −0.15%)	−1.7% (−6.2% to −0.52%)	−3.1% (−12.9% to −0.72%)	−0.66% (−2.2% to −0.24%)	−2.5% (−9.4% to −0.78%)	−4.5% (−19.4% to −1.0%)
GIN	−0.09% (−0.29% to −0.03%)	−0.37% (−1.4% to −0.10%)	−0.69% (−3.4% to −0.15%)	−0.26% (−0.83% to −0.09%)	−0.99% (−3.9% to −0.26%)	−1.8% (−9.0% to −0.37%)	−0.40% (−1.3% to −0.14%)	−1.5% (−5.9% to −0.39%)	−2.5% (−13.0% to −0.53%)
GMB	−0.08% (−0.19% to −0.04%)	−0.35% (−0.97% to −0.15%)	−0.70% (−2.3% to −0.25%)	−0.23% (−0.54% to −0.11%)	−0.93% (−2.6% to −0.39%)	−1.8% (−5.9% to −0.63%)	−0.37% (−0.85% to −0.17%)	−1.4% (−3.9% to −0.59%)	−2.5% (−8.5% to −0.90%)

ISO3 Code	Additional 1% reduction in ARI scenario			Additional 3% reduction in ARI scenario			Additional 5% reduction in ARI scenario		
	2030 (95% UI)	2040 (95% UI)	2050 (95% UI)	2030 (95% UI)	2040 (95% UI)	2050 (95% UI)	2030 (95% UI)	2040 (95% UI)	2050 (95% UI)
GNB	−0.27% (−0.66% to −0.09%)	−1.3% (−2.8% to −0.32%)	−2.8% (−5.3% to −0.56%)	−0.77% (−1.9% to −0.25%)	−3.5% (−7.9% to −0.86%)	−7.2% (−15.1% to −1.4%)	−1.2% (−3.0% to −0.39%)	−5.2% (−12.4% to −1.3%)	−10.4% (−24.6% to −2.1%)
GRC	−0.00% (−0.02% to −0.00%)	−0.02% (−0.09% to −0.00%)	−0.03% (−0.25% to −0.01%)	−0.01% (−0.05% to −0.00%)	−0.05% (−0.25% to −0.01%)	−0.08% (−0.63% to −0.01%)	−0.02% (−0.07% to −0.01%)	−0.07% (−0.37% to −0.02%)	−0.11% (−0.88% to −0.02%)
GRD	−0.00% (−0.00% to −0.00%)	−0.01% (−0.02% to −0.00%)	−0.01% (−0.06% to −0.00%)	−0.01% (−0.01% to −0.00%)	−0.02% (−0.06% to −0.01%)	−0.04% (−0.15% to −0.01%)	−0.01% (−0.02% to −0.00%)	−0.03% (−0.08% to −0.01%)	−0.05% (−0.21% to −0.01%)
GTM	−0.08% (−0.21% to −0.03%)	−0.34% (−1.1% to −0.11%)	−0.71% (−3.1% to −0.18%)	−0.22% (−0.58% to −0.09%)	−0.92% (−3.1% to −0.30%)	−1.8% (−7.9% to −0.46%)	−0.35% (−0.92% to −0.14%)	−1.4% (−4.6% to −0.44%)	−2.6% (−11.3% to −0.66%)
GUY	−0.08% (−0.23% to −0.03%)	−0.34% (−1.2% to −0.10%)	−0.65% (−3.0% to −0.16%)	−0.22% (−0.65% to −0.08%)	−0.90% (−3.2% to −0.26%)	−1.7% (−7.7% to −0.40%)	−0.35% (−1.0% to −0.13%)	−1.3% (−4.9% to −0.39%)	−2.4% (−11.1% to −0.58%)
HKG	−0.05% (−0.16% to −0.01%)	−0.22% (−0.88% to −0.05%)	−0.42% (−2.2% to −0.07%)	−0.15% (−0.47% to −0.04%)	−0.58% (−2.4% to −0.13%)	−1.1% (−5.6% to −0.18%)	−0.23% (−0.74% to −0.07%)	−0.86% (−3.6% to −0.19%)	−1.5% (−8.0% to −0.26%)
HND	−0.02% (−0.08% to −0.01%)	−0.08% (−0.38% to −0.02%)	−0.12% (−0.79% to −0.02%)	−0.06% (−0.24% to −0.02%)	−0.20% (−1.0% to −0.04%)	−0.30% (−2.0% to −0.05%)	−0.10% (−0.38% to −0.03%)	−0.31% (−1.5% to −0.06%)	−0.43% (−2.8% to −0.08%)
HRV	−0.01% (−0.03% to −0.00%)	−0.02% (−0.11% to −0.01%)	−0.03% (−0.19% to −0.01%)	−0.02% (−0.08% to −0.01%)	−0.06% (−0.29% to −0.02%)	−0.09% (−0.49% to −0.02%)	−0.03% (−0.12% to −0.01%)	−0.09% (−0.44% to −0.03%)	−0.13% (−0.70% to −0.03%)
HTI	−0.14% (−0.38% to −0.05%)	−0.58% (−1.9% to −0.15%)	−1.1% (−4.4% to −0.22%)	−0.39% (−1.1% to −0.13%)	−1.6% (−5.3% to −0.40%)	−2.9% (−11.7% to −0.57%)	−0.61% (−1.7% to −0.21%)	−2.4% (−8.1% to −0.60%)	−4.2% (−17.3% to −0.82%)
HUN	−0.01% (−0.02% to −0.00%)	−0.03% (−0.08% to −0.01%)	−0.04% (−0.15% to −0.01%)	−0.02% (−0.06% to −0.01%)	−0.07% (−0.22% to −0.01%)	−0.10% (−0.39% to −0.02%)	−0.03% (−0.09% to −0.01%)	−0.10% (−0.32% to −0.02%)	−0.14% (−0.56% to −0.02%)
IDN	−0.27% (−0.50% to −0.13%)	−1.2% (−2.3% to −0.51%)	−2.6% (−4.7% to −0.89%)	−0.77% (−1.4% to −0.37%)	−3.3% (−6.3% to −1.4%)	−6.8% (−13.2% to −2.3%)	−1.2% (−2.3% to −0.59%)	−5.0% (−9.7% to −2.1%)	−9.8% (−20.6% to −3.3%)
IRL	−0.01% (−0.02% to −0.00%)	−0.02% (−0.11% to −0.01%)	−0.04% (−0.27% to −0.01%)	−0.02% (−0.06% to −0.01%)	−0.06% (−0.29% to −0.02%)	−0.09% (−0.67% to −0.02%)	−0.03% (−0.09% to −0.01%)	−0.09% (−0.43% to −0.03%)	−0.13% (−0.95% to −0.03%)
IRN	−0.02% (−0.07% to −0.01%)	−0.09% (−0.36% to −0.02%)	−0.19% (−0.92% to −0.04%)	−0.06% (−0.19% to −0.02%)	−0.25% (−0.96% to −0.07%)	−0.48% (−2.3% to −0.10%)	−0.10% (−0.30% to −0.03%)	−0.38% (−1.4% to −0.10%)	−0.69% (−3.3% to −0.14%)
IRQ	−0.06% (−0.21% to −0.02%)	−0.30% (−1.3% to −0.07%)	−0.71% (−4.1% to −0.13%)	−0.17% (−0.58% to −0.05%)	−0.80% (−3.5% to −0.19%)	−1.8% (−10.7% to −0.33%)	−0.27% (−0.93% to −0.08%)	−1.2% (−5.3% to −0.28%)	−2.5% (−15.6% to −0.47%)
ISL	−0.00% (−0.01% to −0.00%)	−0.01% (−0.05% to −0.00%)	−0.02% (−0.14% to −0.00%)	−0.01% (−0.03% to −0.00%)	−0.03% (−0.13% to −0.01%)	−0.06% (−0.35% to −0.01%)	−0.01% (−0.04% to −0.00%)	−0.05% (−0.20% to −0.01%)	−0.09% (−0.49% to −0.02%)
ISR	−0.00% (−0.01% to −0.00%)	−0.02% (−0.06% to −0.00%)	−0.03% (−0.12% to −0.00%)	−0.01% (−0.03% to −0.00%)	−0.05% (−0.15% to −0.01%)	−0.08% (−0.31% to −0.01%)	−0.02% (−0.05% to −0.01%)	−0.07% (−0.23% to −0.01%)	−0.11% (−0.44% to −0.02%)
ITA	−0.01% (−0.02% to −0.00%)	−0.02% (−0.09% to −0.01%)	−0.04% (−0.23% to −0.01%)	−0.02% (−0.05% to −0.01%)	−0.06% (−0.24% to −0.02%)	−0.11% (−0.58% to −0.02%)	−0.03% (−0.07% to −0.01%)	−0.09% (−0.36% to −0.03%)	−0.16% (−0.81% to −0.04%)
JAM	−0.01% (−0.01% to −0.00%)	−0.02% (−0.07% to −0.01%)	−0.04% (−0.19% to −0.01%)	−0.01% (−0.04% to −0.01%)	−0.06% (−0.19% to −0.02%)	−0.11% (−0.48% to −0.02%)	−0.02% (−0.06% to −0.01%)	−0.09% (−0.28% to −0.02%)	−0.15% (−0.67% to −0.03%)
JOR	−0.00% (−0.01% to −0.00%)	−0.02% (−0.07% to −0.01%)	−0.03% (−0.20% to −0.01%)	−0.01% (−0.04% to −0.01%)	−0.05% (−0.19% to −0.02%)	−0.09% (−0.50% to −0.02%)	−0.02% (−0.06% to −0.01%)	−0.07% (−0.29% to −0.02%)	−0.12% (−0.70% to −0.03%)
JPN	−0.01% (−0.05% to −0.00%)	−0.05% (−0.22% to −0.01%)	−0.08% (−0.51% to −0.02%)	−0.04% (−0.13% to −0.01%)	−0.12% (−0.59% to −0.03%)	−0.20% (−1.3% to −0.05%)	−0.06% (−0.20% to −0.02%)	−0.18% (−0.88% to −0.05%)	−0.28% (−1.8% to −0.07%)
KAZ	−0.12% (−0.32% to −0.04%)	−0.51% (−1.7% to −0.13%)	−1.0% (−4.2% to −0.21%)	−0.33% (−0.92% to −0.11%)	−1.4% (−4.7% to −0.36%)	−2.6% (−11.1% to −0.55%)	−0.52% (−1.5% to −0.17%)	−2.1% (−7.1% to −0.54%)	−3.7% (−16.2% to −0.78%)
KEN	−0.20% (−0.45% to −0.07%)	−0.98% (−2.5% to −0.29%)	−2.3% (−5.5% to −0.54%)	−0.58% (−1.3% to −0.19%)	−2.6% (−6.9% to −0.79%)	−5.8% (−15.5% to −1.4%)	−0.91% (−2.0% to −0.30%)	−4.0% (−10.5% to −1.2%)	−8.3% (−23.7% to −2.0%)
KGZ	−0.11% (−0.34% to −0.05%)	−0.45% (−1.6% to −0.15%)	−0.84% (−3.7% to −0.22%)	−0.32% (−0.96% to −0.13%)	−1.2% (−4.4% to −0.40%)	−2.1% (−9.7% to −0.57%)	−0.50% (−1.5% to −0.20%)	−1.8% (−6.6% to −0.59%)	−3.1% (−14.1% to −0.83%)
KHM	−0.18% (−0.41% to −0.08%)	−0.75% (−1.9% to −0.30%)	−1.4% (−4.1% to −0.47%)	−0.51% (−1.2% to −0.23%)	−2.0% (−5.3% to −0.80%)	−3.7% (−11.1% to −1.2%)	−0.80% (−1.9% to −0.36%)	−3.0% (−8.2% to −1.2%)	−5.3% (−16.6% to −1.7%)

ISO3 Code	Additional 1% reduction in ARI scenario			Additional 3% reduction in ARI scenario			Additional 5% reduction in ARI scenario		
	2030 (95% UI)	2040 (95% UI)	2050 (95% UI)	2030 (95% UI)	2040 (95% UI)	2050 (95% UI)	2030 (95% UI)	2040 (95% UI)	2050 (95% UI)
KNA	−0.01% (−0.03% to −0.00%)	−0.05% (−0.20% to −0.01%)	−0.11% (−0.63% to −0.02%)	−0.03% (−0.09% to −0.01%)	−0.13% (−0.53% to −0.04%)	−0.28% (−1.6% to −0.06%)	−0.05% (−0.14% to −0.02%)	−0.19% (−0.78% to −0.05%)	−0.39% (−2.2% to −0.09%)
KOR	−0.05% (−0.16% to −0.02%)	−0.19% (−0.90% to −0.06%)	−0.38% (−2.2% to −0.09%)	−0.13% (−0.47% to −0.05%)	−0.52% (−2.4% to −0.16%)	−0.97% (−5.7% to −0.23%)	−0.20% (−0.74% to −0.08%)	−0.78% (−3.6% to −0.25%)	−1.4% (−8.1% to −0.34%)
KWT	−0.02% (−0.05% to −0.01%)	−0.09% (−0.27% to −0.02%)	−0.16% (−0.63% to −0.04%)	−0.06% (−0.14% to −0.02%)	−0.23% (−0.71% to −0.06%)	−0.41% (−1.6% to −0.09%)	−0.09% (−0.22% to −0.03%)	−0.35% (−1.1% to −0.10%)	−0.58% (−2.2% to −0.13%)
LAO	−0.14% (−0.35% to −0.05%)	−0.59% (−1.6% to −0.17%)	−1.1% (−3.5% to −0.24%)	−0.40% (−1.00% to −0.14%)	−1.6% (−4.4% to −0.45%)	−2.8% (−9.3% to −0.62%)	−0.63% (−1.6% to −0.22%)	−2.4% (−6.6% to −0.67%)	−4.0% (−13.7% to −0.91%)
LBN	−0.01% (−0.04% to −0.00%)	−0.07% (−0.27% to −0.01%)	−0.14% (−0.82% to −0.02%)	−0.04% (−0.12% to −0.01%)	−0.18% (−0.72% to −0.04%)	−0.35% (−2.1% to −0.06%)	−0.07% (−0.19% to −0.02%)	−0.27% (−1.1% to −0.06%)	−0.50% (−2.9% to −0.08%)
LBR	−0.33% (−0.76% to −0.13%)	−1.6% (−2.9% to −0.61%)	−3.5% (−5.3% to −1.2%)	−0.93% (−2.2% to −0.38%)	−4.4% (−8.6% to −1.6%)	−9.5% (−15.2% to −3.0%)	−1.5% (−3.5% to −0.60%)	−6.6% (−13.7% to −2.5%)	−13.9% (−24.6% to −4.4%)
LBY	−0.04% (−0.11% to −0.01%)	−0.17% (−0.62% to −0.06%)	−0.35% (−1.7% to −0.09%)	−0.11% (−0.30% to −0.04%)	−0.44% (−1.7% to −0.15%)	−0.88% (−4.4% to −0.24%)	−0.17% (−0.48% to −0.07%)	−0.66% (−2.5% to −0.22%)	−1.2% (−6.2% to −0.35%)
LCA	−0.01% (−0.02% to −0.00%)	−0.03% (−0.09% to −0.01%)	−0.05% (−0.20% to −0.01%)	−0.02% (−0.05% to −0.01%)	−0.07% (−0.23% to −0.02%)	−0.12% (−0.51% to −0.03%)	−0.03% (−0.08% to −0.01%)	−0.10% (−0.34% to −0.03%)	−0.17% (−0.71% to −0.04%)
LKA	−0.07% (−0.21% to −0.03%)	−0.34% (−1.2% to −0.10%)	−0.70% (−3.1% to −0.17%)	−0.21% (−0.60% to −0.07%)	−0.91% (−3.4% to −0.26%)	−1.8% (−8.0% to −0.42%)	−0.33% (−0.95% to −0.12%)	−1.4% (−5.1% to −0.38%)	−2.5% (−11.6% to −0.60%)
LTU	−0.06% (−0.16% to −0.02%)	−0.24% (−0.78% to −0.06%)	−0.46% (−1.9% to −0.10%)	−0.17% (−0.45% to −0.05%)	−0.64% (−2.1% to −0.17%)	−1.2% (−4.9% to −0.26%)	−0.27% (−0.71% to −0.08%)	−0.96% (−3.1% to −0.26%)	−1.7% (−6.9% to −0.37%)
LUX	−0.01% (−0.02% to −0.00%)	−0.02% (−0.09% to −0.01%)	−0.04% (−0.20% to −0.01%)	−0.02% (−0.05% to −0.01%)	−0.06% (−0.23% to −0.02%)	−0.09% (−0.50% to −0.02%)	−0.03% (−0.08% to −0.01%)	−0.08% (−0.34% to −0.03%)	−0.13% (−0.71% to −0.03%)
LVA	−0.02% (−0.08% to −0.01%)	−0.08% (−0.33% to −0.02%)	−0.13% (−0.62% to −0.03%)	−0.07% (−0.21% to −0.02%)	−0.22% (−0.88% to −0.06%)	−0.32% (−1.6% to −0.07%)	−0.11% (−0.34% to −0.03%)	−0.33% (−1.3% to −0.09%)	−0.47% (−2.2% to −0.10%)
MAC	−0.07% (−0.24% to −0.02%)	−0.30% (−1.2% to −0.07%)	−0.58% (−2.9% to −0.10%)	−0.20% (−0.68% to −0.06%)	−0.79% (−3.3% to −0.19%)	−1.5% (−7.4% to −0.26%)	−0.31% (−1.1% to −0.09%)	−1.2% (−4.9% to −0.29%)	−2.1% (−10.7% to −0.37%)
MAR	−0.09% (−0.24% to −0.03%)	−0.39% (−1.4% to −0.10%)	−0.83% (−3.5% to −0.17%)	−0.24% (−0.68% to −0.08%)	−1.0% (−3.7% to −0.26%)	−2.1% (−9.1% to −0.43%)	−0.38% (−1.1% to −0.12%)	−1.6% (−5.5% to −0.39%)	−3.0% (−13.2% to −0.61%)
MDA	−0.19% (−0.50% to −0.07%)	−0.92% (−2.6% to −0.30%)	−2.1% (−5.5% to −0.51%)	−0.55% (−1.4% to −0.21%)	−2.5% (−7.2% to −0.79%)	−5.3% (−15.8% to −1.3%)	−0.87% (−2.3% to −0.33%)	−3.7% (−11.1% to −1.2%)	−7.6% (−23.9% to −1.9%)
MDG	−0.18% (−0.43% to −0.06%)	−0.81% (−2.1% to −0.20%)	−1.6% (−4.5% to −0.33%)	−0.51% (−1.2% to −0.16%)	−2.2% (−5.8% to −0.53%)	−4.1% (−12.3% to −0.83%)	−0.80% (−2.0% to −0.25%)	−3.3% (−8.9% to −0.80%)	−5.9% (−18.4% to −1.2%)
MEX	−0.01% (−0.05% to −0.01%)	−0.05% (−0.27% to −0.02%)	−0.08% (−0.62% to −0.02%)	−0.03% (−0.14% to −0.01%)	−0.12% (−0.71% to −0.04%)	−0.20% (−1.6% to −0.06%)	−0.05% (−0.23% to −0.02%)	−0.18% (−1.1% to −0.06%)	−0.28% (−2.2% to −0.08%)
MKD	−0.01% (−0.04% to −0.00%)	−0.05% (−0.19% to −0.01%)	−0.08% (−0.42% to −0.02%)	−0.04% (−0.11% to −0.01%)	−0.12% (−0.49% to −0.03%)	−0.20% (−1.1% to −0.04%)	−0.06% (−0.17% to −0.02%)	−0.19% (−0.74% to −0.05%)	−0.29% (−1.5% to −0.06%)
MLI	−0.06% (−0.19% to −0.02%)	−0.26% (−0.98% to −0.08%)	−0.53% (−2.4% to −0.12%)	−0.17% (−0.55% to −0.06%)	−0.70% (−2.6% to −0.20%)	−1.3% (−6.1% to −0.30%)	−0.27% (−0.86% to −0.10%)	−1.0% (−4.0% to −0.30%)	−1.9% (−8.8% to −0.43%)
MLT	−0.02% (−0.08% to −0.01%)	−0.11% (−0.60% to −0.04%)	−0.30% (−2.6% to −0.08%)	−0.05% (−0.22% to −0.02%)	−0.28% (−1.6% to −0.10%)	−0.75% (−6.5% to −0.19%)	−0.08% (−0.34% to −0.03%)	−0.42% (−2.4% to −0.15%)	−1.1% (−9.3% to −0.27%)
MMR	−0.18% (−0.38% to −0.07%)	−0.77% (−1.8% to −0.23%)	−1.5% (−3.8% to −0.34%)	−0.51% (−1.1% to −0.19%)	−2.1% (−4.9% to −0.63%)	−3.8% (−10.2% to −0.87%)	−0.80% (−1.7% to −0.29%)	−3.1% (−7.4% to −0.94%)	−5.5% (−15.1% to −1.3%)
MNE	−0.01% (−0.03% to −0.00%)	−0.04% (−0.12% to −0.01%)	−0.06% (−0.21% to −0.01%)	−0.03% (−0.09% to −0.01%)	−0.11% (−0.32% to −0.03%)	−0.16% (−0.54% to −0.03%)	−0.05% (−0.14% to −0.02%)	−0.17% (−0.47% to −0.04%)	−0.23% (−0.77% to −0.05%)
MNG	−0.08% (−0.24% to −0.02%)	−0.32% (−1.2% to −0.08%)	−0.57% (−2.8% to −0.11%)	−0.22% (−0.69% to −0.07%)	−0.85% (−3.1% to −0.20%)	−1.5% (−7.2% to −0.29%)	−0.35% (−1.1% to −0.10%)	−1.3% (−4.7% to −0.31%)	−2.1% (−10.4% to −0.42%)
MOZ	−0.25% (−0.52% to −0.09%)	−1.2% (−2.3% to −0.38%)	−2.4% (−4.8% to −0.68%)	−0.72% (−1.5% to −0.25%)	−3.1% (−6.7% to −1.0%)	−6.3% (−13.8% to −1.7%)	−1.1% (−2.4% to −0.40%)	−4.7% (−10.4% to −1.5%)	−9.1% (−20.7% to −2.5%)

ISO3 Code	Additional 1% reduction in ARI scenario			Additional 3% reduction in ARI scenario			Additional 5% reduction in ARI scenario		
	2030 (95% UI)	2040 (95% UI)	2050 (95% UI)	2030 (95% UI)	2040 (95% UI)	2050 (95% UI)	2030 (95% UI)	2040 (95% UI)	2050 (95% UI)
MRT	−0.06% (−0.19% to −0.02%)	−0.23% (−0.87% to −0.06%)	−0.39% (−1.8% to −0.08%)	−0.18% (−0.53% to −0.06%)	−0.63% (−2.3% to −0.16%)	−0.99% (−4.7% to −0.20%)	−0.28% (−0.84% to −0.09%)	−0.94% (−3.5% to −0.24%)	−1.4% (−6.8% to −0.28%)
MUS	−0.03% (−0.07% to −0.01%)	−0.12% (−0.37% to −0.03%)	−0.23% (−0.98% to −0.05%)	−0.08% (−0.20% to −0.03%)	−0.31% (−0.99% to −0.09%)	−0.59% (−2.5% to −0.14%)	−0.12% (−0.32% to −0.04%)	−0.46% (−1.5% to −0.14%)	−0.83% (−3.5% to −0.20%)
MWI	−0.24% (−0.59% to −0.07%)	−1.2% (−2.8% to −0.28%)	−2.6% (−5.5% to −0.49%)	−0.69% (−1.7% to −0.20%)	−3.2% (−7.8% to −0.75%)	−6.7% (−15.6% to −1.3%)	−1.1% (−2.7% to −0.32%)	−4.8% (−12.1% to −1.1%)	−9.7% (−24.5% to −1.8%)
MYS	−0.11% (−0.27% to −0.04%)	−0.54% (−1.6% to −0.15%)	−1.3% (−4.5% to −0.25%)	−0.30% (−0.78% to −0.11%)	−1.4% (−4.3% to −0.40%)	−3.3% (−11.7% to −0.64%)	−0.47% (−1.2% to −0.17%)	−2.2% (−6.5% to −0.60%)	−4.6% (−17.1% to −0.92%)
NAM	−0.35% (−0.67% to −0.13%)	−1.6% (−2.5% to −0.53%)	−3.3% (−4.8% to −0.91%)	−1.00% (−1.9% to −0.38%)	−4.3% (−7.3% to −1.4%)	−8.8% (−13.8% to −2.5%)	−1.6% (−3.1% to −0.59%)	−6.6% (−11.7% to −2.1%)	−13.0% (−21.7% to −3.5%)
NER	−0.05% (−0.15% to −0.02%)	−0.17% (−0.70% to −0.04%)	−0.29% (−1.4% to −0.06%)	−0.14% (−0.42% to −0.05%)	−0.47% (−1.9% to −0.12%)	−0.75% (−3.7% to −0.16%)	−0.22% (−0.67% to −0.07%)	−0.70% (−2.8% to −0.18%)	−1.1% (−5.3% to −0.23%)
NGA	−0.21% (−0.45% to −0.09%)	−1.00% (−2.2% to −0.37%)	−2.1% (−5.0% to −0.70%)	−0.58% (−1.3% to −0.26%)	−2.7% (−6.2% to −1.00%)	−5.5% (−13.6% to −1.8%)	−0.92% (−2.0% to −0.40%)	−4.0% (−9.4% to −1.5%)	−7.9% (−20.3% to −2.6%)
NIC	−0.02% (−0.08% to −0.01%)	−0.11% (−0.49% to −0.03%)	−0.24% (−1.5% to −0.05%)	−0.07% (−0.23% to −0.02%)	−0.28% (−1.3% to −0.08%)	−0.60% (−3.9% to −0.12%)	−0.10% (−0.36% to −0.04%)	−0.42% (−2.0% to −0.11%)	−0.85% (−5.5% to −0.17%)
NLD	−0.01% (−0.01% to −0.00%)	−0.02% (−0.08% to −0.01%)	−0.04% (−0.20% to −0.01%)	−0.01% (−0.04% to −0.01%)	−0.05% (−0.22% to −0.01%)	−0.09% (−0.51% to −0.02%)	−0.02% (−0.07% to −0.01%)	−0.08% (−0.33% to −0.02%)	−0.13% (−0.71% to −0.03%)
NOR	−0.01% (−0.04% to −0.00%)	−0.05% (−0.24% to −0.01%)	−0.10% (−0.76% to −0.02%)	−0.03% (−0.10% to −0.01%)	−0.12% (−0.63% to −0.03%)	−0.26% (−1.9% to −0.04%)	−0.04% (−0.16% to −0.01%)	−0.19% (−0.94% to −0.04%)	−0.37% (−2.7% to −0.06%)
NPL	−0.13% (−0.34% to −0.03%)	−0.60% (−1.8% to −0.13%)	−1.3% (−4.3% to −0.19%)	−0.37% (−0.98% to −0.10%)	−1.6% (−4.8% to −0.34%)	−3.2% (−11.4% to −0.50%)	−0.58% (−1.6% to −0.15%)	−2.4% (−7.3% to −0.51%)	−4.5% (−16.8% to −0.72%)
NZL	−0.01% (−0.02% to −0.00%)	−0.03% (−0.12% to −0.01%)	−0.06% (−0.33% to −0.01%)	−0.02% (−0.06% to −0.01%)	−0.08% (−0.33% to −0.02%)	−0.15% (−0.83% to −0.03%)	−0.03% (−0.10% to −0.01%)	−0.12% (−0.49% to −0.03%)	−0.21% (−1.2% to −0.04%)
OMN	−0.01% (−0.03% to −0.00%)	−0.03% (−0.14% to −0.01%)	−0.06% (−0.37% to −0.01%)	−0.02% (−0.08% to −0.01%)	−0.09% (−0.38% to −0.02%)	−0.16% (−0.93% to −0.04%)	−0.04% (−0.13% to −0.01%)	−0.14% (−0.57% to −0.04%)	−0.23% (−1.3% to −0.05%)
PAK	−0.18% (−0.35% to −0.07%)	−0.76% (−1.7% to −0.28%)	−1.6% (−3.8% to −0.48%)	−0.51% (−0.99% to −0.21%)	−2.1% (−4.7% to −0.74%)	−4.0% (−10.1% to −1.2%)	−0.80% (−1.6% to −0.33%)	−3.1% (−7.1% to −1.1%)	−5.8% (−14.8% to −1.8%)
PAN	−0.05% (−0.14% to −0.02%)	−0.23% (−0.77% to −0.06%)	−0.46% (−2.2% to −0.10%)	−0.15% (−0.41% to −0.05%)	−0.61% (−2.1% to −0.16%)	−1.2% (−5.6% to −0.25%)	−0.23% (−0.65% to −0.07%)	−0.91% (−3.1% to −0.23%)	−1.6% (−8.0% to −0.36%)
PER	−0.08% (−0.30% to −0.03%)	−0.34% (−1.4% to −0.10%)	−0.63% (−3.3% to −0.16%)	−0.23% (−0.85% to −0.09%)	−0.90% (−3.9% to −0.28%)	−1.6% (−8.7% to −0.40%)	−0.36% (−1.3% to −0.14%)	−1.3% (−5.9% to −0.42%)	−2.3% (−12.6% to −0.58%)
POL	−0.02% (−0.05% to −0.01%)	−0.06% (−0.23% to −0.01%)	−0.10% (−0.53% to −0.02%)	−0.04% (−0.13% to −0.01%)	−0.15% (−0.61% to −0.04%)	−0.26% (−1.3% to −0.05%)	−0.07% (−0.21% to −0.02%)	−0.23% (−0.91% to −0.06%)	−0.37% (−1.9% to −0.08%)
PRT	−0.01% (−0.05% to −0.00%)	−0.05% (−0.23% to −0.01%)	−0.09% (−0.50% to −0.02%)	−0.04% (−0.13% to −0.01%)	−0.14% (−0.61% to −0.04%)	−0.23% (−1.3% to −0.05%)	−0.06% (−0.21% to −0.02%)	−0.21% (−0.90% to −0.05%)	−0.32% (−1.8% to −0.07%)
PRY	−0.04% (−0.14% to −0.01%)	−0.17% (−0.79% to −0.04%)	−0.33% (−2.1% to −0.07%)	−0.11% (−0.39% to −0.03%)	−0.45% (−2.1% to −0.12%)	−0.83% (−5.4% to −0.18%)	−0.18% (−0.62% to −0.05%)	−0.68% (−3.2% to −0.17%)	−1.2% (−7.6% to −0.25%)
PSE	−0.00% (−0.02% to −0.00%)	−0.02% (−0.10% to −0.00%)	−0.03% (−0.27% to −0.01%)	−0.01% (−0.05% to −0.00%)	−0.05% (−0.27% to −0.01%)	−0.08% (−0.68% to −0.01%)	−0.02% (−0.07% to −0.01%)	−0.07% (−0.40% to −0.02%)	−0.12% (−0.96% to −0.02%)
ROU	−0.04% (−0.12% to −0.01%)	−0.15% (−0.54% to −0.04%)	−0.25% (−1.1% to −0.05%)	−0.12% (−0.34% to −0.04%)	−0.41% (−1.5% to −0.10%)	−0.64% (−2.8% to −0.12%)	−0.19% (−0.54% to −0.06%)	−0.62% (−2.2% to −0.15%)	−0.92% (−4.0% to −0.17%)
RUS	−0.09% (−0.24% to −0.03%)	−0.37% (−1.4% to −0.09%)	−0.73% (−3.4% to −0.13%)	−0.25% (−0.68% to −0.08%)	−0.99% (−3.7% to −0.23%)	−1.9% (−8.8% to −0.34%)	−0.40% (−1.1% to −0.12%)	−1.5% (−5.6% to −0.35%)	−2.6% (−12.7% to −0.49%)
RWA	−0.07% (−0.15% to −0.03%)	−0.32% (−0.80% to −0.09%)	−0.70% (−2.1% to −0.16%)	−0.20% (−0.42% to −0.08%)	−0.86% (−2.2% to −0.25%)	−1.8% (−5.3% to −0.40%)	−0.31% (−0.66% to −0.12%)	−1.3% (−3.2% to −0.38%)	−2.5% (−7.5% to −0.57%)
SAU	−0.01% (−0.05% to −0.00%)	−0.06% (−0.29% to −0.01%)	−0.11% (−0.80% to −0.02%)	−0.04% (−0.14% to −0.01%)	−0.15% (−0.78% to −0.04%)	−0.28% (−2.0% to −0.05%)	−0.06% (−0.22% to −0.02%)	−0.23% (−1.2% to −0.05%)	−0.40% (−2.8% to −0.07%)

ISO3 Code	Additional 1% reduction in ARI scenario			Additional 3% reduction in ARI scenario			Additional 5% reduction in ARI scenario		
	2030 (95% UI)	2040 (95% UI)	2050 (95% UI)	2030 (95% UI)	2040 (95% UI)	2050 (95% UI)	2030 (95% UI)	2040 (95% UI)	2050 (95% UI)
SDN	−0.09% (−0.24% to −0.03%)	−0.36% (−1.2% to −0.09%)	−0.68% (−2.9% to −0.14%)	−0.24% (−0.68% to −0.07%)	−0.97% (−3.3% to −0.24%)	−1.7% (−7.4% to −0.37%)	−0.39% (−1.1% to −0.12%)	−1.5% (−5.0% to −0.36%)	−2.5% (−10.6% to −0.53%)
SEN	−0.12% (−0.31% to −0.04%)	−0.55% (−1.6% to −0.15%)	−1.1% (−3.9% to −0.24%)	−0.35% (−0.87% to −0.11%)	−1.5% (−4.4% to −0.40%)	−2.8% (−10.2% to −0.60%)	−0.56% (−1.4% to −0.18%)	−2.2% (−6.6% to −0.60%)	−4.0% (−14.9% to −0.87%)
SGP	−0.04% (−0.11% to −0.01%)	−0.17% (−0.61% to −0.04%)	−0.36% (−1.7% to −0.06%)	−0.12% (−0.31% to −0.03%)	−0.46% (−1.6% to −0.10%)	−0.91% (−4.3% to −0.16%)	−0.18% (−0.49% to −0.05%)	−0.69% (−2.4% to −0.16%)	−1.3% (−6.0% to −0.23%)
SLE	−0.23% (−0.58% to −0.08%)	−1.0% (−2.6% to −0.30%)	−2.1% (−5.0% to −0.50%)	−0.64% (−1.7% to −0.21%)	−2.8% (−7.6% to −0.79%)	−5.5% (−14.2% to −1.3%)	−1.0% (−2.7% to −0.34%)	−4.2% (−11.9% to −1.2%)	−8.0% (−22.1% to −1.9%)
SLV	−0.04% (−0.11% to −0.01%)	−0.17% (−0.77% to −0.03%)	−0.39% (−2.5% to −0.05%)	−0.10% (−0.32% to −0.02%)	−0.46% (−2.1% to −0.08%)	−0.99% (−6.3% to −0.12%)	−0.16% (−0.50% to −0.04%)	−0.68% (−3.1% to −0.12%)	−1.4% (−9.0% to −0.17%)
SOM	−0.23% (−0.64% to −0.09%)	−1.1% (−2.7% to −0.38%)	−2.3% (−5.2% to −0.65%)	−0.65% (−1.8% to −0.26%)	−2.9% (−7.7% to −1.0%)	−5.8% (−14.6% to −1.7%)	−1.0% (−2.9% to −0.42%)	−4.4% (−12.1% to −1.5%)	−8.4% (−23.2% to −2.4%)
SRB	−0.01% (−0.07% to −0.00%)	−0.05% (−0.31% to −0.01%)	−0.08% (−0.67% to −0.02%)	−0.04% (−0.19% to −0.01%)	−0.14% (−0.82% to −0.03%)	−0.21% (−1.7% to −0.04%)	−0.07% (−0.30% to −0.02%)	−0.20% (−1.2% to −0.04%)	−0.30% (−2.4% to −0.06%)
SSD	−0.22% (−0.54% to −0.05%)	−1.1% (−2.7% to −0.21%)	−2.6% (−5.7% to −0.38%)	−0.62% (−1.5% to −0.15%)	−3.0% (−7.6% to −0.57%)	−6.6% (−16.0% to −0.97%)	−0.97% (−2.5% to −0.24%)	−4.4% (−11.7% to −0.85%)	−9.5% (−25.0% to −1.4%)
SUR	−0.02% (−0.05% to −0.01%)	−0.06% (−0.22% to −0.02%)	−0.09% (−0.44% to −0.02%)	−0.05% (−0.15% to −0.02%)	−0.16% (−0.60% to −0.05%)	−0.23% (−1.1% to −0.06%)	−0.08% (−0.24% to −0.03%)	−0.23% (−0.89% to −0.07%)	−0.33% (−1.6% to −0.09%)
SVK	−0.00% (−0.01% to −0.00%)	−0.01% (−0.05% to −0.00%)	−0.02% (−0.09% to −0.00%)	−0.01% (−0.04% to −0.00%)	−0.03% (−0.13% to −0.01%)	−0.05% (−0.22% to −0.01%)	−0.02% (−0.06% to −0.01%)	−0.05% (−0.19% to −0.01%)	−0.06% (−0.31% to −0.01%)
SVN	−0.00% (−0.02% to −0.00%)	−0.01% (−0.07% to −0.00%)	−0.02% (−0.14% to −0.00%)	−0.01% (−0.05% to −0.00%)	−0.04% (−0.20% to −0.01%)	−0.05% (−0.35% to −0.01%)	−0.02% (−0.08% to −0.01%)	−0.06% (−0.30% to −0.01%)	−0.08% (−0.50% to −0.02%)
SWE	−0.01% (−0.03% to −0.00%)	−0.04% (−0.25% to −0.01%)	−0.11% (−0.88% to −0.02%)	−0.02% (−0.10% to −0.01%)	−0.12% (−0.66% to −0.03%)	−0.27% (−2.2% to −0.05%)	−0.04% (−0.15% to −0.01%)	−0.18% (−0.98% to −0.05%)	−0.39% (−3.1% to −0.07%)
SWZ	−0.43% (−0.77% to −0.15%)	−2.0% (−2.8% to −0.67%)	−4.3% (−5.4% to −1.3%)	−1.2% (−2.2% to −0.44%)	−5.6% (−8.2% to −1.8%)	−11.7% (−15.6% to −3.4%)	−1.9% (−3.5% to −0.69%)	−8.6% (−13.3% to −2.7%)	−18.2% (−24.5% to −4.9%)
SYC	−0.02% (−0.07% to −0.01%)	−0.09% (−0.42% to −0.03%)	−0.18% (−1.1% to −0.04%)	−0.06% (−0.20% to −0.02%)	−0.25% (−1.1% to −0.07%)	−0.45% (−2.8% to −0.10%)	−0.10% (−0.32% to −0.03%)	−0.37% (−1.7% to −0.11%)	−0.63% (−4.0% to −0.15%)
SYR	−0.01% (−0.04% to −0.00%)	−0.04% (−0.18% to −0.01%)	−0.06% (−0.42% to −0.01%)	−0.03% (−0.11% to −0.01%)	−0.10% (−0.49% to −0.03%)	−0.15% (−1.0% to −0.03%)	−0.05% (−0.17% to −0.02%)	−0.15% (−0.73% to −0.04%)	−0.22% (−1.5% to −0.05%)
TCD	−0.15% (−0.40% to −0.04%)	−0.67% (−2.1% to −0.16%)	−1.4% (−5.2% to −0.25%)	−0.42% (−1.1% to −0.12%)	−1.8% (−5.9% to −0.42%)	−3.7% (−13.9% to −0.64%)	−0.66% (−1.8% to −0.19%)	−2.7% (−8.9% to −0.63%)	−5.2% (−20.6% to −0.92%)
TGO	−0.07% (−0.18% to −0.02%)	−0.37% (−1.2% to −0.09%)	−0.92% (−3.8% to −0.16%)	−0.20% (−0.52% to −0.06%)	−0.99% (−3.4% to −0.23%)	−2.3% (−10.0% to −0.40%)	−0.31% (−0.83% to −0.09%)	−1.5% (−5.1% to −0.34%)	−3.3% (−14.4% to −0.57%)
THA	−0.14% (−0.31% to −0.06%)	−0.62% (−1.6% to −0.23%)	−1.2% (−3.5% to −0.39%)	−0.41% (−0.87% to −0.18%)	−1.7% (−4.3% to −0.61%)	−3.2% (−9.2% to −0.98%)	−0.64% (−1.4% to −0.28%)	−2.5% (−6.5% to −0.91%)	−4.6% (−13.4% to −1.4%)
TJK	−0.08% (−0.19% to −0.02%)	−0.29% (−0.82% to −0.07%)	−0.48% (−1.8% to −0.10%)	−0.22% (−0.55% to −0.07%)	−0.77% (−2.2% to −0.20%)	−1.2% (−4.5% to −0.25%)	−0.35% (−0.87% to −0.11%)	−1.2% (−3.3% to −0.30%)	−1.7% (−6.4% to −0.37%)
TKM	−0.05% (−0.16% to −0.01%)	−0.18% (−0.70% to −0.03%)	−0.28% (−1.4% to −0.04%)	−0.15% (−0.46% to −0.03%)	−0.48% (−1.9% to −0.09%)	−0.71% (−3.5% to −0.11%)	−0.23% (−0.73% to −0.05%)	−0.72% (−2.8% to −0.13%)	−1.0% (−5.1% to −0.16%)
TTO	−0.02% (−0.07% to −0.01%)	−0.13% (−0.48% to −0.03%)	−0.32% (−1.5% to −0.06%)	−0.07% (−0.20% to −0.03%)	−0.34% (−1.3% to −0.09%)	−0.81% (−3.7% to −0.14%)	−0.11% (−0.31% to −0.04%)	−0.51% (−1.9% to −0.13%)	−1.2% (−5.3% to −0.21%)
TUN	−0.04% (−0.11% to −0.01%)	−0.21% (−0.72% to −0.05%)	−0.47% (−2.2% to −0.09%)	−0.12% (−0.32% to −0.04%)	−0.55% (−1.9% to −0.13%)	−1.2% (−5.5% to −0.22%)	−0.18% (−0.50% to −0.06%)	−0.82% (−2.9% to −0.20%)	−1.7% (−7.8% to −0.31%)
TUR	−0.01% (−0.03% to −0.00%)	−0.04% (−0.14% to −0.01%)	−0.07% (−0.32% to −0.02%)	−0.03% (−0.09% to −0.01%)	−0.11% (−0.38% to −0.03%)	−0.17% (−0.80% to −0.05%)	−0.05% (−0.15% to −0.02%)	−0.16% (−0.57% to −0.05%)	−0.24% (−1.1% to −0.07%)
TZA	−0.30% (−0.62% to −0.12%)	−1.4% (−2.6% to −0.48%)	−3.0% (−5.0% to −0.84%)	−0.85% (−1.8% to −0.33%)	−3.7% (−7.5% to −1.3%)	−7.8% (−14.5% to −2.1%)	−1.3% (−2.8% to −0.53%)	−5.7% (−11.6% to −1.9%)	−11.3% (−22.6% to −3.3%)

ISO3 Code	Additional 1% reduction in ARI scenario			Additional 3% reduction in ARI scenario			Additional 5% reduction in ARI scenario		
	2030 (95% UI)	2040 (95% UI)	2050 (95% UI)	2030 (95% UI)	2040 (95% UI)	2050 (95% UI)	2030 (95% UI)	2040 (95% UI)	2050 (95% UI)
UGA	−0.05% (−0.17% to −0.02%)	−0.20% (−0.74% to −0.07%)	−0.35% (−1.5% to −0.10%)	−0.15% (−0.49% to −0.06%)	−0.54% (−2.0% to −0.18%)	−0.89% (−3.8% to −0.25%)	−0.24% (−0.77% to −0.09%)	−0.82% (−3.0% to −0.27%)	−1.3% (−5.4% to −0.36%)
UKR	−0.09% (−0.25% to −0.03%)	−0.40% (−1.3% to −0.11%)	−0.81% (−3.2% to −0.18%)	−0.26% (−0.71% to −0.09%)	−1.1% (−3.6% to −0.29%)	−2.1% (−8.4% to −0.45%)	−0.42% (−1.1% to −0.14%)	−1.6% (−5.4% to −0.43%)	−2.9% (−12.1% to −0.63%)
URY	−0.03% (−0.08% to −0.01%)	−0.13% (−0.52% to −0.03%)	−0.30% (−1.6% to −0.05%)	−0.07% (−0.24% to −0.02%)	−0.34% (−1.4% to −0.09%)	−0.77% (−4.0% to −0.14%)	−0.12% (−0.38% to −0.04%)	−0.51% (−2.1% to −0.13%)	−1.1% (−5.6% to −0.19%)
USA	−0.00% (−0.01% to −0.00%)	−0.01% (−0.03% to −0.00%)	−0.01% (−0.07% to −0.00%)	−0.01% (−0.02% to −0.00%)	−0.02% (−0.09% to −0.01%)	−0.03% (−0.18% to −0.01%)	−0.01% (−0.03% to −0.00%)	−0.03% (−0.13% to −0.01%)	−0.04% (−0.25% to −0.01%)
UZB	−0.12% (−0.29% to −0.03%)	−0.54% (−1.5% to −0.13%)	−1.2% (−4.0% to −0.23%)	−0.33% (−0.83% to −0.10%)	−1.5% (−4.2% to −0.35%)	−3.0% (−10.4% to −0.58%)	−0.52% (−1.3% to −0.15%)	−2.2% (−6.3% to −0.52%)	−4.3% (−15.2% to −0.84%)
VCT	−0.02% (−0.07% to −0.01%)	−0.08% (−0.35% to −0.02%)	−0.14% (−0.88% to −0.04%)	−0.06% (−0.19% to −0.02%)	−0.21% (−0.93% to −0.07%)	−0.36% (−2.2% to −0.09%)	−0.09% (−0.30% to −0.03%)	−0.31% (−1.4% to −0.10%)	−0.51% (−3.1% to −0.13%)
VEN	−0.02% (−0.07% to −0.01%)	−0.09% (−0.38% to −0.02%)	−0.18% (−0.96% to −0.03%)	−0.06% (−0.19% to −0.02%)	−0.24% (−1.0% to −0.05%)	−0.46% (−2.4% to −0.07%)	−0.10% (−0.30% to −0.03%)	−0.36% (−1.5% to −0.08%)	−0.66% (−3.4% to −0.11%)
YEM	−0.02% (−0.08% to −0.01%)	−0.08% (−0.33% to −0.02%)	−0.12% (−0.66% to −0.03%)	−0.06% (−0.21% to −0.02%)	−0.21% (−0.88% to −0.06%)	−0.31% (−1.7% to −0.08%)	−0.10% (−0.34% to −0.03%)	−0.32% (−1.3% to −0.09%)	−0.45% (−2.4% to −0.11%)
ZAF	−0.42% (−0.77% to −0.15%)	−2.0% (−2.8% to −0.64%)	−4.0% (−5.3% to −1.2%)	−1.2% (−2.2% to −0.43%)	−5.4% (−8.4% to −1.7%)	−11.6% (−15.5% to −3.2%)	−1.9% (−3.6% to −0.68%)	−8.3% (−13.5% to −2.6%)	−17.4% (−24.8% to −4.6%)
ZMB	−0.17% (−0.45% to −0.06%)	−0.72% (−2.2% to −0.19%)	−1.4% (−4.7% to −0.31%)	−0.47% (−1.3% to −0.16%)	−1.9% (−6.0% to −0.52%)	−3.6% (−13.0% to −0.79%)	−0.74% (−2.0% to −0.25%)	−2.9% (−9.3% to −0.78%)	−5.2% (−19.6% to −1.1%)
ZWE	−0.14% (−0.39% to −0.05%)	−0.60% (−1.8% to −0.19%)	−1.2% (−4.3% to −0.28%)	−0.41% (−1.1% to −0.15%)	−1.6% (−5.1% to −0.50%)	−3.2% (−11.4% to −0.73%)	−0.64% (−1.8% to −0.24%)	−2.4% (−7.7% to −0.75%)	−4.6% (−16.7% to −1.0%)

Appendix Table 9. Projected prevalence of overall Mtb immunoreactivity by country, age group, and year (2024 and 2050), under the status quo and three scenarios of additional ARI reduction (1%, 3%, and 5%)

		Status Quo		2050 (95% UI) with additional reduction in ARI scenario		
Country	Age group, y	2024 (95% UI)	2050 (95% UI)	Additional 1% reduction	Additional 3% reduction	Additional 5% reduction
China	0–14	1.3% (0.67% to 2.8%)	0.54% (0.11% to 2.9%)	0.42% (0.09% to 2.3%)	0.25% (0.05% to 1.4%)	0.15% (0.03% to 0.84%)
	15–34	7.3% (5.9% to 10.2%)	2.8% (0.89% to 11.0%)	2.4% (0.80% to 9.4%)	1.9% (0.66% to 7.1%)	1.5% (0.53% to 5.4%)
	35–54	17.9% (14.0% to 25.6%)	6.9% (3.7% to 16.9%)	6.5% (3.6% to 15.4%)	5.9% (3.4% to 13.0%)	5.4% (3.3% to 11.4%)
	55–74	41.0% (22.7% to 80.9%)	16.4% (12.9% to 25.2%)	16.0% (12.7% to 23.8%)	15.5% (12.3% to 21.8%)	15.1% (12.0% to 20.4%)
	75+	72.8% (28.3% to 99.9%)	34.9% (23.4% to 62.5%)	34.4% (22.9% to 62.4%)	33.7% (22.3% to 62.3%)	33.3% (21.8% to 62.2%)
	Total	14.7% (10.7% to 22.7%)	5.8% (3.4% to 13.5%)	5.5% (3.3% to 12.2%)	5.0% (3.1% to 10.1%)	4.7% (2.9% to 8.7%)
India	0–14	3.3% (1.8% to 5.8%)	1.3% (0.33% to 5.6%)	0.98% (0.25% to 4.4%)	0.59% (0.15% to 2.7%)	0.36% (0.09% to 1.7%)
	15–34	18.1% (14.5% to 22.7%)	7.0% (2.6% to 20.3%)	6.1% (2.3% to 17.6%)	4.8% (1.8% to 13.4%)	3.8% (1.5% to 10.3%)
	35–54	34.2% (29.1% to 42.6%)	16.7% (9.4% to 34.2%)	15.8% (9.1% to 31.4%)	14.3% (8.6% to 26.9%)	13.5% (8.2% to 23.6%)
	55–74	50.8% (42.4% to 60.0%)	34.4% (26.2% to 49.1%)	33.8% (25.9% to 47.1%)	32.6% (25.5% to 43.8%)	31.8% (25.2% to 41.4%)
	75+	66.7% (51.5% to 87.5%)	49.3% (40.1% to 62.0%)	48.7% (39.8% to 60.5%)	48.0% (39.4% to 58.7%)	47.4% (39.2% to 57.3%)
	Total	25.4% (20.4% to 30.8%)	13.2% (7.7% to 26.2%)	12.5% (7.4% to 23.9%)	11.3% (6.8% to 20.1%)	10.5% (6.4% to 18.2%)
Philippines	0–14	6.4% (3.6% to 11.8%)	3.0% (0.69% to 14.5%)	2.4% (0.54% to 11.6%)	1.4% (0.32% to 7.3%)	0.87% (0.19% to 4.5%)
	15–34	29.4% (23.7% to 38.0%)	14.8% (5.0% to 44.5%)	12.9% (4.4% to 39.2%)	10.1% (3.5% to 30.3%)	8.0% (2.8% to 23.5%)
	35–54	54.1% (46.2% to 66.5%)	30.5% (17.4% to 58.8%)	29.0% (16.8% to 55.3%)	26.3% (15.9% to 49.3%)	24.5% (15.2% to 44.0%)
	55–74	75.9% (58.3% to 96.8%)	54.3% (45.2% to 72.8%)	53.1% (44.6% to 71.0%)	51.7% (43.8% to 67.3%)	50.4% (42.9% to 64.2%)
	75+	91.2% (61.6% to 100.0%)	76.2% (63.4% to 92.3%)	75.4% (63.0% to 91.9%)	74.2% (62.0% to 91.4%)	73.1% (61.0% to 90.9%)
	Total	40.1% (32.6% to 49.8%)	23.0% (14.3% to 48.5%)	21.7% (13.8% to 44.3%)	19.5% (12.5% to 37.3%)	18.0% (11.3% to 31.9%)
Viet Nam	0–14	2.8% (1.3% to 6.5%)	1.1% (0.14% to 9.3%)	0.89% (0.11% to 7.4%)	0.54% (0.06% to 4.6%)	0.32% (0.04% to 2.8%)
	15–34	15.6% (12.3% to 21.1%)	6.1% (1.4% to 28.6%)	5.3% (1.3% to 24.8%)	4.1% (1.1% to 18.7%)	3.2% (0.89% to 14.3%)
	35–54	37.2% (26.7% to 57.8%)	14.3% (7.0% to 39.0%)	13.6% (6.8% to 35.8%)	12.4% (6.6% to 30.8%)	11.5% (6.5% to 27.0%)
	55–74	71.8% (34.7% to 99.6%)	34.0% (24.9% to 52.8%)	33.6% (24.7% to 50.1%)	32.4% (24.5% to 45.9%)	31.6% (24.0% to 44.4%)
	75+	95.6% (37.5% to 100.0%)	65.0% (37.5% to 97.2%)	63.2% (36.9% to 97.2%)	62.3% (36.4% to 97.2%)	62.1% (36.1% to 97.2%)
	Total	27.7% (19.5% to 41.2%)	11.7% (6.9% to 30.5%)	11.4% (6.8% to 27.4%)	10.4% (6.4% to 22.6%)	9.6% (6.1% to 19.5%)

Appendix Table 10. Projected percent of individuals with recently acquired Mtb immunoreactivity (i.e., within the past two years) by country, age group, and year (2024 and 2050), under the status quo and three scenarios of additional ARI reduction (1%, 3%, and 5%)

		Status Quo		2050 (95% UI) with additional reduction in ARI scenario		
Country	Age group, y	2024 (95% UI)	2050 (95% UI)	Additional 1% reduction	Additional 3% reduction	Additional 5% reduction
China	0–14	0.30% (0.13% to 0.72%)	0.12% (0.02% to 0.72%)	0.09% (0.02% to 0.55%)	0.05% (0.01% to 0.31%)	0.03% (0.00% to 0.17%)
	15–34	0.36% (0.16% to 0.84%)	0.15% (0.03% to 0.84%)	0.11% (0.02% to 0.64%)	0.06% (0.01% to 0.37%)	0.03% (0.01% to 0.21%)
	35–54	0.32% (0.14% to 0.74%)	0.14% (0.03% to 0.77%)	0.10% (0.02% to 0.59%)	0.06% (0.01% to 0.34%)	0.03% (0.01% to 0.19%)
	55–74	0.23% (0.05% to 0.63%)	0.12% (0.02% to 0.71%)	0.09% (0.02% to 0.54%)	0.05% (0.01% to 0.31%)	0.03% (0.01% to 0.18%)
	75+	0.10% (0.00% to 0.47%)	0.10% (0.02% to 0.61%)	0.07% (0.01% to 0.47%)	0.04% (0.01% to 0.28%)	0.02% (0.00% to 0.16%)
	Total	0.32% (0.14% to 0.74%)	0.14% (0.03% to 0.78%)	0.10% (0.02% to 0.60%)	0.06% (0.01% to 0.34%)	0.03% (0.01% to 0.19%)
India	0–14	0.77% (0.39% to 1.4%)	0.28% (0.06% to 1.5%)	0.21% (0.05% to 1.1%)	0.11% (0.03% to 0.64%)	0.06% (0.01% to 0.36%)
	15–34	0.83% (0.42% to 1.6%)	0.33% (0.08% to 1.6%)	0.25% (0.06% to 1.2%)	0.14% (0.03% to 0.73%)	0.08% (0.02% to 0.42%)
	35–54	0.65% (0.33% to 1.3%)	0.30% (0.07% to 1.3%)	0.22% (0.05% to 1.0%)	0.13% (0.03% to 0.62%)	0.07% (0.02% to 0.36%)
	55–74	0.48% (0.25% to 0.99%)	0.23% (0.06% to 1.0%)	0.17% (0.04% to 0.78%)	0.10% (0.02% to 0.47%)	0.05% (0.01% to 0.27%)
	75+	0.31% (0.10% to 0.72%)	0.18% (0.05% to 0.74%)	0.13% (0.03% to 0.58%)	0.08% (0.02% to 0.34%)	0.04% (0.01% to 0.20%)
	Total	0.71% (0.35% to 1.4%)	0.30% (0.07% to 1.4%)	0.22% (0.05% to 1.1%)	0.12% (0.03% to 0.65%)	0.07% (0.02% to 0.37%)
Philippines	0–14	1.5% (0.74% to 3.0%)	0.69% (0.13% to 3.6%)	0.52% (0.10% to 2.8%)	0.29% (0.05% to 1.7%)	0.16% (0.03% to 0.97%)
	15–34	1.4% (0.74% to 2.8%)	0.77% (0.16% to 3.0%)	0.59% (0.12% to 2.5%)	0.34% (0.06% to 1.6%)	0.19% (0.03% to 0.98%)
	35–54	0.94% (0.41% to 1.9%)	0.63% (0.14% to 2.1%)	0.48% (0.10% to 1.8%)	0.28% (0.06% to 1.1%)	0.16% (0.03% to 0.70%)
	55–74	0.48% (0.05% to 1.5%)	0.43% (0.09% to 1.4%)	0.33% (0.07% to 1.1%)	0.19% (0.04% to 0.75%)	0.10% (0.02% to 0.46%)
	75+	0.16% (0.00% to 1.3%)	0.24% (0.02% to 0.98%)	0.18% (0.02% to 0.79%)	0.10% (0.01% to 0.49%)	0.06% (0.01% to 0.30%)
	Total	1.1% (0.56% to 2.3%)	0.67% (0.14% to 2.6%)	0.51% (0.10% to 2.1%)	0.29% (0.06% to 1.3%)	0.16% (0.03% to 0.80%)
Viet Nam	0–14	0.64% (0.23% to 1.8%)	0.26% (0.02% to 2.4%)	0.19% (0.02% to 1.8%)	0.11% (0.01% to 1.1%)	0.06% (0.01% to 0.61%)
	15–34	0.71% (0.25% to 1.9%)	0.31% (0.03% to 2.3%)	0.23% (0.02% to 1.9%)	0.13% (0.01% to 1.1%)	0.07% (0.01% to 0.67%)
	35–54	0.49% (0.17% to 1.6%)	0.28% (0.03% to 1.9%)	0.21% (0.02% to 1.5%)	0.12% (0.01% to 0.93%)	0.06% (0.01% to 0.56%)
	55–74	0.22% (0.00% to 1.3%)	0.21% (0.02% to 1.5%)	0.16% (0.02% to 1.2%)	0.09% (0.01% to 0.73%)	0.05% (0.00% to 0.44%)
	75+	0.03% (0.00% to 1.2%)	0.11% (0.00% to 1.3%)	0.08% (0.00% to 1.0%)	0.05% (0.00% to 0.62%)	0.03% (0.00% to 0.37%)
	Total	0.55% (0.21% to 1.7%)	0.28% (0.03% to 2.1%)	0.21% (0.02% to 1.6%)	0.12% (0.01% to 0.99%)	0.06% (0.01% to 0.59%)

Appendix Table 11. Sensitivity analysis considering reversion. Projected proportion of individuals with recently acquired Mtb immunoreactivity (within the past two years) by country, age group, and year (2024 and 2050), under the status quo and three scenarios of additional ARI reduction (1%, 3%, and 5%)

		Status Quo		2050 (95% UI) with additional reduction in ARI scenario		
Country	Age group, y	2024 (95% UI)	2050 (95% UI)	Additional 1% reduction	Additional 3% reduction	Additional 5% reduction
China	0–14	0.87% (0.39% to 2.1%)	0.34% (0.06% to 2.1%)	0.26% (0.05% to 1.6%)	0.14% (0.03% to 0.90%)	0.08% (0.01% to 0.50%)
	15–34	1.0% (0.47% to 2.4%)	0.42% (0.08% to 2.4%)	0.32% (0.06% to 1.9%)	0.18% (0.03% to 1.1%)	0.10% (0.02% to 0.61%)
	35–54	0.92% (0.40% to 2.2%)	0.41% (0.08% to 2.2%)	0.30% (0.06% to 1.7%)	0.17% (0.03% to 0.99%)	0.09% (0.02% to 0.56%)
	55–74	0.66% (0.13% to 1.8%)	0.36% (0.07% to 2.0%)	0.27% (0.05% to 1.6%)	0.15% (0.03% to 0.91%)	0.08% (0.01% to 0.52%)
	75+	0.29% (0.00% to 1.4%)	0.28% (0.05% to 1.8%)	0.21% (0.04% to 1.4%)	0.12% (0.02% to 0.80%)	0.06% (0.01% to 0.46%)
	Total	0.93% (0.41% to 2.1%)	0.40% (0.08% to 2.3%)	0.30% (0.06% to 1.7%)	0.17% (0.03% to 0.99%)	0.09% (0.02% to 0.56%)
India	0–14	2.2% (1.1% to 4.2%)	0.80% (0.19% to 4.2%)	0.60% (0.14% to 3.2%)	0.33% (0.07% to 1.9%)	0.18% (0.04% to 1.1%)
	15–34	2.4% (1.2% to 4.5%)	0.96% (0.23% to 4.6%)	0.72% (0.17% to 3.6%)	0.40% (0.09% to 2.1%)	0.22% (0.05% to 1.2%)
	35–54	1.9% (0.96% to 3.7%)	0.86% (0.21% to 3.8%)	0.65% (0.16% to 3.0%)	0.36% (0.09% to 1.8%)	0.20% (0.05% to 1.0%)
	55–74	1.4% (0.73% to 2.9%)	0.67% (0.17% to 2.9%)	0.51% (0.13% to 2.3%)	0.28% (0.07% to 1.4%)	0.16% (0.04% to 0.79%)
	75+	0.90% (0.29% to 2.1%)	0.52% (0.13% to 2.2%)	0.39% (0.10% to 1.7%)	0.22% (0.05% to 0.99%)	0.12% (0.03% to 0.57%)
	Total	2.1% (1.0% to 4.0%)	0.86% (0.21% to 4.1%)	0.64% (0.15% to 3.2%)	0.36% (0.08% to 1.9%)	0.20% (0.04% to 1.1%)
Philippines	0–14	4.3% (2.1% to 8.7%)	2.0% (0.38% to 10.5%)	1.5% (0.28% to 8.2%)	0.84% (0.15% to 4.9%)	0.46% (0.08% to 2.8%)
	15–34	4.2% (2.1% to 8.1%)	2.2% (0.46% to 8.8%)	1.7% (0.34% to 7.2%)	0.97% (0.19% to 4.7%)	0.55% (0.10% to 2.8%)

Country	Age group, y	Status Quo		2050 (95% UI) with additional reduction in ARI scenario		
		2024 (95% UI)	2050 (95% UI)	Additional 1% reduction	Additional 3% reduction	Additional 5% reduction
	35–54	2.7% (1.2% to 5.5%)	1.8% (0.40% to 6.2%)	1.4% (0.30% to 5.1%)	0.81% (0.16% to 3.3%)	0.45% (0.09% to 2.0%)
	55–74	1.4% (0.14% to 4.4%)	1.2% (0.26% to 4.0%)	0.94% (0.19% to 3.3%)	0.54% (0.10% to 2.2%)	0.30% (0.06% to 1.3%)
	75+	0.46% (0.00% to 3.7%)	0.68% (0.07% to 2.8%)	0.52% (0.05% to 2.3%)	0.30% (0.03% to 1.4%)	0.17% (0.02% to 0.86%)
	Total	3.3% (1.6% to 6.6%)	1.9% (0.41% to 7.4%)	1.5% (0.30% to 6.0%)	0.84% (0.16% to 3.8%)	0.47% (0.09% to 2.3%)
Viet Nam	0–14	1.9% (0.65% to 5.1%)	0.74% (0.07% to 6.9%)	0.55% (0.05% to 5.3%)	0.31% (0.03% to 3.1%)	0.17% (0.01% to 1.8%)
	15–34	2.1% (0.73% to 5.6%)	0.89% (0.09% to 6.8%)	0.67% (0.07% to 5.4%)	0.38% (0.04% to 3.3%)	0.21% (0.02% to 2.0%)
	35–54	1.4% (0.49% to 4.6%)	0.81% (0.08% to 5.5%)	0.61% (0.06% to 4.4%)	0.34% (0.03% to 2.7%)	0.19% (0.02% to 1.6%)
	55–74	0.63% (0.01% to 3.8%)	0.62% (0.07% to 4.3%)	0.46% (0.05% to 3.5%)	0.26% (0.03% to 2.1%)	0.14% (0.01% to 1.3%)
	75+	0.10% (0.00% to 3.4%)	0.32% (0.01% to 3.8%)	0.24% (0.00% to 3.0%)	0.13% (0.00% to 1.8%)	0.07% (0.00% to 1.1%)
	Total	1.6% (0.60% to 5.0%)	0.80% (0.08% to 6.0%)	0.60% (0.06% to 4.7%)	0.34% (0.03% to 2.9%)	0.18% (0.02% to 1.7%)

Appendix Table 12. Projected annual risk of tuberculosis disease per 100,000 persons in the year of arrival by country, age group, and year (2024 and 2050), under the status quo and three scenarios of additional ARI reduction (1%, 3%, and 5%), China, India, Philippines, and Viet Nam

Country	Age group, y	Status Quo		2050 (95% UI) with additional reduction in ARI scenario		
		2024 (95% UI)	2050 (95% UI)	Additional 1% reduction	Additional 3% reduction	Additional 5% reduction
China	0–14	5.9 (2.7–14.8)	2.3 (0.4–14.5)	1.7 (0.3–11.0)	1.0 (0.2–6.3)	0.5 (0.1–3.6)
	15–34	11.1 (6.5–22.5)	4.5 (1.0–21.8)	3.6 (0.8–17.4)	2.4 (0.6–11.4)	1.6 (0.4–7.4)
	35–54	18.5 (13.4–27.4)	7.2 (3.1–24.8)	6.3 (2.9–20.8)	5.1 (2.6–15.1)	4.4 (2.4–11.5)
	55–74	34.3 (21.1–57.9)	13.5 (9.1–30.4)	12.9 (8.8–26.9)	11.8 (8.5–21.6)	11.3 (8.1–17.9)
	75+	54.0 (25.3–76.7)	27.8 (17.9–47.4)	27.0 (17.2–46.5)	25.5 (16.0–45.9)	24.7 (15.3–44.7)
	Total	16.4 (11.0–24.9)	6.4 (3.0–22.7)	5.5 (2.7–18.6)	4.4 (2.3–12.7)	3.8 (2.1–9.1)
India	0–14	14.6 (7.3–32.9)	5.5 (1.2–29.2)	4.1 (0.9–22.4)	2.3 (0.5–13.0)	1.3 (0.3–7.4)
	15–34	25.9 (16.7–46.3)	10.6 (3.1–41.4)	8.6 (2.6–33.7)	5.8 (1.8–22.2)	4.0 (1.3–14.5)
	35–54	35.4 (25.5–51.8)	16.7 (7.9–45.1)	14.9 (7.4–38.2)	12.3 (6.7–28.0)	10.6 (6.1–22.8)
	55–74	44.4 (33.8–59.6)	28.3 (19.1–51.6)	26.7 (18.5–46.3)	25.0 (17.7–38.3)	23.7 (17.2–35.3)
	75+	53.6 (41.7–71.6)	38.3 (27.5–58.5)	37.1 (26.9–56.4)	35.5 (26.0–51.8)	34.6 (25.6–49.4)
	Total	29.7 (20.1–47.4)	14.5 (6.8–41.9)	12.7 (6.3–34.7)	10.1 (5.4–24.2)	8.5 (4.9–18.3)
Philippines	0–14	29.6 (13.8–58.6)	13.4 (2.5–61.5)	10.1 (1.9–48.4)	5.7 (1.0–29.2)	3.2 (0.6–17.1)
	15–34	44.8 (28.9–72.7)	23.5 (6.2–79.1)	19.3 (5.1–67.5)	13.0 (3.5–47.5)	8.9 (2.6–32.8)
	35–54	54.4 (42.2–75.2)	32.0 (13.6–76.5)	28.4 (12.7–67.9)	23.1 (11.5–53.4)	20.0 (10.8–41.9)
	55–74	63.2 (51.4–80.2)	45.5 (33.1–78.4)	43.6 (31.8–72.2)	39.9 (29.9–61.2)	38.1 (28.9–53.6)
	75+	67.1 (54.3–84.8)	58.6 (45.3–81.3)	57.2 (44.0–77.5)	55.2 (41.7–73.7)	53.4 (40.0–73.0)
	Total	47.9 (34.5–72.0)	27.1 (12.2–76.0)	23.5 (11.0–65.6)	18.5 (9.0–48.7)	15.2 (8.1–36.7)
Viet Nam	0–14	12.4 (4.6–30.2)	4.8 (0.5–41.4)	3.6 (0.4–31.9)	2.0 (0.2–18.8)	1.1 (0.1–10.8)
	15–34	22.6 (13.4–45.5)	9.1 (1.4–53.9)	7.4 (1.2–44.1)	4.9 (0.9–29.9)	3.4 (0.7–20.3)
	35–54	36.7 (25.2–56.1)	14.7 (5.7–57.9)	13.1 (5.4–50.0)	10.7 (4.9–37.6)	9.2 (4.6–29.1)
	55–74	55.5 (33.4–79.1)	28.5 (18.3–59.9)	27.1 (17.7–53.7)	25.1 (17.1–43.9)	23.6 (16.3–36.7)
	75+	64.9 (35.8–86.0)	50.4 (29.4–78.3)	49.1 (27.4–78.1)	47.6 (26.5–76.4)	45.7 (25.2–76.4)
	Total	30.2 (21.2–47.3)	12.9 (5.9–53.9)	11.1 (5.5–45.5)	9.1 (5.0–32.8)	7.9 (4.6–24.4)

Appendix Table 13. Sensitivity analysis considering reversion. Projected annual risk of tuberculosis disease per 100,000 persons in the year of arrival by country, age group, and year (2024 and 2050), under the status quo and three scenarios of additional ARI reduction (1%, 3%, and 5%), China, India, Philippines, and Viet Nam

		Status Quo		2050 (95% UI) with additional reduction in ARI scenario		
Country	Age group, y	2024 (95% UI)	2050 (95% UI)	Additional 1% reduction	Additional 3% reduction	Additional 5% reduction
China	0–14	15.4 (6.8–39.2)	5.9 (1.1–38.1)	4.4 (0.8–29.0)	2.5 (0.4–16.5)	1.4 (0.2–9.3)
	15–34	22.3 (11.7–50.8)	9.0 (1.8–49.9)	7.0 (1.5–38.9)	4.3 (0.9–23.6)	2.7 (0.6–14.3)
	35–54	28.5 (18.4–53.5)	11.7 (3.8–51.2)	9.8 (3.4–40.8)	7.0 (2.9–26.2)	5.3 (2.6–17.5)
	55–74	41.9 (28.1–64.3)	17.3 (10.4–52.1)	15.6 (9.7–42.9)	13.3 (9.0–31.1)	12.0 (8.5–23.9)
	75+	57.8 (34.6–76.8)	31.1 (20.0–56.3)	29.9 (18.9–51.1)	27.6 (17.8–47.2)	25.8 (16.3–46.0)
	Total	26.0 (16.9–50.2)	10.6 (3.7–48.7)	8.8 (3.4–38.5)	6.2 (2.9–24.1)	4.7 (2.4–15.6)
India	0–14	37.9 (17.9–86.3)	13.9 (3.1–77.1)	10.4 (2.3–59.0)	5.8 (1.2–34.0)	3.2 (0.7–19.3)
	15–34	51.2 (29.7–103.4)	20.9 (5.5–92.2)	16.3 (4.4–73.3)	10.1 (2.8–45.7)	6.5 (1.9–28.3)
	35–54	55.3 (35.4–97.1)	26.0 (10.6–87.7)	21.7 (9.6–72.0)	16.1 (7.6–49.0)	12.9 (6.7–33.9)
	55–74	59.1 (43.3–90.8)	35.6 (21.8–83.0)	32.4 (20.7–70.7)	27.9 (18.9–52.6)	25.4 (17.7–42.3)
	75+	63.8 (50.1–88.1)	44.1 (30.1–79.0)	41.4 (29.2–71.1)	38.0 (27.3–59.0)	36.0 (26.1–53.8)
	Total	51.1 (30.9–96.4)	23.5 (9.2–86.9)	19.5 (8.1–70.1)	14.2 (6.6–45.8)	10.8 (5.5–30.5)
Philippines	0–14	77.3 (35.7–156.2)	35.6 (6.4–158.7)	26.9 (4.8–122.8)	15.1 (2.6–72.8)	8.3 (1.4–42.4)
	15–34	90.3 (50.6–160.5)	47.4 (11.1–164.5)	36.9 (9.0–133.9)	23.0 (5.8–89.7)	15.1 (3.7–59.5)
	35–54	83.4 (57.6–129.6)	51.9 (18.3–140.5)	43.6 (16.1–117.8)	31.9 (13.1–87.7)	24.7 (11.6–64.7)
	55–74	78.3 (59.5–117.9)	58.5 (37.0–118.6)	52.3 (35.6–104.1)	45.6 (32.8–82.1)	41.2 (30.2–67.4)
	75+	75.9 (59.6–114.6)	66.0 (49.9–109.2)	63.6 (47.4–97.5)	58.9 (45.1–84.4)	55.7 (42.0–74.2)
	Total	83.7 (51.5–139.5)	48.5 (15.6–149.4)	39.4 (14.0–122.4)	27.5 (11.8–86.5)	20.2 (9.4–61.2)
Viet Nam	0–14	32.2 (11.6–78.7)	12.5 (1.2–108.9)	9.4 (0.9–84.3)	5.2 (0.5–49.4)	2.9 (0.2–28.4)
	15–34	44.5 (21.3–95.4)	18.3 (2.2–122.0)	14.2 (1.8–98.6)	8.8 (1.2–63.2)	5.5 (0.9–38.8)
	35–54	52.9 (34.9–95.7)	23.1 (7.1–111.6)	19.4 (6.4–92.8)	14.4 (5.5–63.7)	11.2 (4.9–44.8)
	55–74	64.8 (47.2–95.7)	34.6 (19.9–104.3)	32.0 (18.9–89.8)	28.2 (17.9–64.7)	25.6 (17.3–49.6)
	75+	68.7 (53.3–93.8)	55.8 (34.6–98.4)	54.3 (32.1–88.0)	50.9 (29.6–78.2)	48.0 (26.5–78.0)
	Total	46.9 (29.4–92.1)	20.8 (7.2–113.0)	17.4 (6.6–92.3)	12.6 (5.6–61.2)	9.6 (5.0–38.6)

Appendix Table 14. Projected annual risk of tuberculosis disease per 100,000 persons in the year of arrival by country and year (2024 and 2050), under the status quo and three scenarios of additional ARI reduction (1%, 3%, and 5%), including analysis accounting for reversion, all other countries

	Status quo		2050 (95% UI) with additional reduction in ARI scenario		
ISO3 Code	2024 (95% UI)	2050 (95% UI)	1% reduction	3% reduction	5% reduction
PRIMARY ANALYSIS					
AFG	35.7 (25.4–59.8)	23.9 (8.9–77.8)	20.4 (8.1–65.3)	15.1 (7.1–46.0)	12.0 (6.4–33.1)
AGO	54.2 (33.5–95.0)	56.4 (16.9–135.1)	47.7 (14.7–124.1)	34.2 (11.5–104.6)	25.7 (9.7–75.9)
ALB	3.9 (2.2–9.0)	2.2 (0.7–14.8)	1.9 (0.6–11.7)	1.4 (0.5–7.4)	1.1 (0.4–4.8)
ARE	2.7 (0.8–10.7)	0.3 (0.1–0.9)	0.2 (0.1–0.8)	0.2 (0.1–0.8)	0.2 (0.1–0.8)
ARG	5.5 (3.6–9.1)	2.0 (0.9–10.0)	1.8 (0.8–8.2)	1.4 (0.7–5.5)	1.2 (0.7–3.9)
ARM	10.3 (5.9–24.6)	8.9 (2.1–46.6)	7.3 (1.9–37.5)	5.2 (1.6–24.2)	3.9 (1.4–15.8)
ATG	1.0 (0.6–2.5)	1.1 (0.3–9.9)	0.9 (0.2–7.8)	0.6 (0.2–4.9)	0.4 (0.1–3.1)
AUS	1.4 (0.8–3.2)	1.1 (0.3–12.0)	0.9 (0.2–9.5)	0.6 (0.2–5.9)	0.5 (0.2–3.7)
AUT	2.6 (1.3–8.6)	0.8 (0.3–2.7)	0.7 (0.3–2.2)	0.6 (0.3–1.5)	0.5 (0.3–1.1)
AZE	35.3 (26.3–45.4)	8.0 (4.9–12.5)	7.9 (4.7–12.4)	7.7 (4.6–12.0)	7.6 (4.5–11.9)
BDI	28.1 (19.2–44.5)	12.5 (5.1–51.5)	11.0 (4.8–42.5)	8.8 (4.3–29.3)	7.4 (3.9–20.6)
BEL	2.5 (1.3–5.9)	1.1 (0.4–4.6)	0.9 (0.4–3.6)	0.7 (0.3–2.4)	0.6 (0.3–1.7)
BEN	15.8 (9.4–24.7)	6.1 (2.4–22.7)	5.4 (2.2–18.6)	4.4 (1.9–12.9)	3.8 (1.8–9.3)

	Status quo		2050 (95% UI) with additional reduction in ARI scenario		
ISO3 Code	2024 (95% UI)	2050 (95% UI)	1% reduction	3% reduction	5% reduction
BFA	13.2 (7.8–23.7)	11.0 (2.8–53.2)	9.2 (2.5–42.9)	6.4 (2.2–27.8)	4.8 (1.9–18.1)
BGD	47.5 (30.8–81.2)	44.4 (15.2–121.5)	37.7 (13.3–111.4)	27.0 (10.1–88.9)	19.8 (8.4–66.7)
BGR	7.8 (4.6–14.1)	2.7 (1.2–16.0)	2.4 (1.1–12.8)	2.0 (1.0–8.3)	1.7 (0.9–5.9)
BHR	4.4 (2.9–7.4)	1.7 (0.6–7.1)	1.5 (0.6–5.7)	1.2 (0.5–3.8)	1.0 (0.5–2.7)
BHS	3.0 (1.7–9.1)	0.9 (0.4–3.2)	0.8 (0.4–2.6)	0.7 (0.4–1.9)	0.6 (0.4–1.4)
BIH	13.0 (8.4–22.7)	5.0 (2.2–25.8)	4.4 (2.0–21.3)	3.6 (1.8–14.5)	3.2 (1.7–10.0)
BLR	13.1 (8.0–27.5)	9.6 (2.6–64.3)	8.1 (2.4–52.6)	5.9 (2.1–34.9)	4.6 (1.8–23.3)
BLZ	6.6 (3.9–13.2)	3.6 (1.2–22.6)	3.0 (1.1–18.3)	2.3 (0.9–12.0)	1.8 (0.8–7.9)
BOL	26.5 (18.9–44.7)	13.5 (5.5–53.5)	11.6 (5.1–44.5)	8.8 (4.5–31.0)	7.4 (4.1–22.4)
BRA	9.5 (5.5–16.1)	4.3 (1.3–19.7)	3.7 (1.2–16.0)	2.8 (1.2–10.6)	2.4 (1.0–7.3)
BRB	0.2 (0.1–1.8)	0.0 (0.0–0.1)	0.0 (0.0–0.1)	0.0 (0.0–0.1)	0.0 (0.0–0.1)
BRN	12.6 (7.7–24.6)	9.6 (2.6–60.0)	7.9 (2.5–48.9)	5.6 (2.1–32.5)	4.2 (1.8–21.7)
BTN	35.2 (26.6–48.0)	9.3 (5.2–21.9)	8.8 (5.0–19.3)	8.3 (4.8–15.1)	7.9 (4.7–13.4)
BWA	40.7 (27.7–63.6)	20.4 (9.3–79.8)	17.8 (8.4–68.8)	14.4 (7.3–50.8)	12.1 (6.6–37.7)
CAF	47.4 (34.5–68.7)	18.9 (9.2–50.0)	17.4 (8.9–43.2)	14.8 (8.4–32.9)	13.4 (7.9–26.1)
CHE	1.9 (1.0–6.9)	0.6 (0.3–3.1)	0.5 (0.2–2.5)	0.4 (0.2–1.6)	0.4 (0.2–1.1)
CHL	4.4 (2.4–12.8)	1.4 (0.6–6.4)	1.3 (0.5–5.2)	1.0 (0.5–3.4)	0.8 (0.4–2.3)
CIV	33.2 (24.5–53.7)	14.0 (6.6–54.5)	12.5 (6.4–45.8)	10.3 (6.0–32.7)	8.9 (5.7–24.1)
CMR	38.3 (24.4–70.5)	28.6 (10.1–93.9)	24.2 (9.1–80.9)	17.9 (7.8–58.9)	13.8 (6.7–42.8)
COD	55.4 (35.7–93.9)	56.3 (15.6–126.0)	47.5 (13.9–116.0)	34.7 (11.3–89.4)	26.0 (10.0–69.0)
COG	57.1 (29.7–125.3)	72.8 (17.4–150.7)	62.2 (15.0–145.1)	45.0 (11.3–129.6)	33.1 (9.3–107.6)
COL	7.6 (4.6–14.0)	3.1 (1.3–15.7)	2.6 (1.2–12.6)	2.1 (1.1–8.3)	1.8 (1.0–5.7)
COM	10.5 (6.2–18.9)	7.0 (2.0–45.7)	5.9 (1.8–36.9)	4.3 (1.5–23.9)	3.3 (1.4–15.6)
CRI	3.8 (2.1–9.4)	1.0 (0.5–6.4)	0.9 (0.4–5.1)	0.8 (0.4–3.4)	0.7 (0.3–2.3)
CUB	3.5 (1.8–11.3)	1.0 (0.4–3.4)	0.9 (0.4–2.9)	0.8 (0.3–2.0)	0.6 (0.3–1.5)
CYP	1.2 (0.7–2.8)	1.4 (0.3–12.1)	1.2 (0.3–9.6)	0.8 (0.2–6.0)	0.5 (0.2–3.8)
CZE	2.8 (1.3–10.0)	0.5 (0.3–1.1)	0.5 (0.2–1.0)	0.4 (0.2–0.9)	0.4 (0.2–0.9)
DEU	2.2 (1.1–8.3)	0.5 (0.3–2.0)	0.5 (0.2–1.6)	0.4 (0.2–1.2)	0.4 (0.2–1.0)
DJI	76.3 (45.2–133.4)	93.9 (29.6–136.9)	83.1 (25.8–132.7)	61.8 (20.4–121.2)	46.4 (17.0–107.1)
DMA	5.3 (0.8–21.4)	0.5 (0.2–3.2)	0.5 (0.2–3.2)	0.4 (0.2–3.2)	0.4 (0.2–3.2)
DNK	1.8 (0.8–4.1)	0.9 (0.2–5.3)	0.8 (0.2–4.2)	0.6 (0.2–2.7)	0.4 (0.2–1.8)
DOM	16.1 (10.9–25.9)	5.0 (2.3–17.8)	4.5 (2.2–14.7)	3.9 (2.0–10.5)	3.4 (1.9–7.9)
DZA	14.9 (10.2–26.6)	8.6 (3.3–31.3)	7.3 (3.0–25.3)	5.3 (2.6–16.8)	4.2 (2.3–11.5)
ECU	15.5 (9.6–26.4)	5.5 (2.5–23.6)	4.8 (2.3–19.2)	4.0 (2.0–13.3)	3.4 (1.9–9.3)
EGY	5.9 (3.4–13.7)	2.4 (1.0–9.8)	2.1 (0.9–7.9)	1.7 (0.8–5.3)	1.4 (0.7–3.6)
ERI	21.5 (14.1–32.8)	9.8 (3.8–37.8)	8.5 (3.5–31.2)	6.7 (3.1–21.7)	5.6 (2.8–15.6)
ESP	3.2 (1.7–7.5)	1.3 (0.5–8.3)	1.2 (0.5–6.6)	0.9 (0.4–4.3)	0.8 (0.4–2.9)
EST	5.8 (3.5–13.1)	1.9 (0.9–6.4)	1.7 (0.8–5.3)	1.4 (0.8–3.7)	1.3 (0.7–2.7)
ETH	29.1 (21.6–42.9)	12.1 (5.6–40.7)	10.7 (5.3–34.0)	8.7 (4.7–24.2)	7.5 (4.5–18.0)
FIN	1.9 (1.0–6.5)	0.6 (0.2–2.3)	0.5 (0.2–1.9)	0.4 (0.2–1.3)	0.3 (0.2–1.0)
FJI	15.4 (8.9–26.3)	10.4 (3.1–46.7)	8.8 (2.9–38.2)	6.4 (2.4–25.7)	4.8 (2.1–17.4)
FRA	2.8 (1.5–7.7)	1.1 (0.4–5.1)	1.0 (0.4–4.1)	0.7 (0.4–2.8)	0.6 (0.3–1.9)
GAB	63.1 (38.6–109.3)	59.0 (18.6–137.2)	50.1 (16.7–130.7)	36.6 (14.0–111.5)	28.0 (12.1–87.6)
GBR	2.7 (1.5–4.9)	2.9 (0.6–14.4)	2.3 (0.5–11.4)	1.6 (0.4–7.3)	1.1 (0.4–4.7)
GEO	28.0 (20.1–38.3)	7.9 (3.9–20.5)	7.4 (3.7–17.4)	6.6 (3.6–13.4)	6.1 (3.5–11.2)
GHA	37.0 (25.2–69.7)	22.7 (8.2–100.1)	19.3 (7.7–84.2)	14.7 (6.9–60.4)	11.9 (6.1–44.0)
GIN	26.7 (18.5–41.7)	12.8 (5.4–65.6)	11.1 (5.0–54.2)	8.8 (4.4–37.1)	7.1 (4.2–26.0)
GMB	19.3 (12.3–29.9)	12.3 (5.0–46.5)	10.3 (4.4–37.9)	7.6 (3.7–25.3)	5.9 (3.1–17.3)

	Status quo		2050 (95% UI) with additional reduction in ARI scenario		
ISO3 Code	2024 (95% UI)	2050 (95% UI)	1% reduction	3% reduction	5% reduction
GNB	50.3 (30.5–87.7)	50.4 (13.4–132.2)	42.6 (11.7–127.7)	30.9 (9.8–105.8)	23.3 (8.7–82.0)
GRC	1.4 (0.8–3.9)	0.5 (0.2–4.8)	0.5 (0.2–3.8)	0.4 (0.2–2.4)	0.3 (0.1–1.5)
GRD	0.3 (0.2–0.5)	0.2 (0.1–1.1)	0.2 (0.1–0.9)	0.1 (0.0–0.6)	0.1 (0.0–0.4)
GTM	17.0 (11.4–30.6)	12.0 (4.0–57.6)	10.1 (3.6–47.3)	7.4 (2.9–31.5)	5.7 (2.4–21.1)
GUY	19.1 (11.9–33.4)	11.3 (3.6–52.4)	9.5 (3.2–43.4)	7.0 (3.0–29.9)	5.6 (2.6–20.9)
HKG	15.7 (10.0–26.9)	7.8 (2.9–40.9)	6.6 (2.7–33.6)	5.0 (2.3–23.0)	4.1 (2.1–16.3)
HND	13.1 (7.5–23.3)	3.2 (1.8–12.8)	3.0 (1.7–10.7)	2.7 (1.5–7.7)	2.5 (1.4–6.0)
HRV	6.0 (3.1–14.9)	1.3 (0.7–3.1)	1.2 (0.6–2.7)	1.1 (0.5–2.3)	1.0 (0.5–2.1)
HTI	33.0 (21.3–59.2)	20.6 (6.8–80.2)	17.7 (6.2–68.4)	13.3 (5.3–49.1)	10.7 (5.1–35.4)
HUN	5.0 (2.5–13.3)	1.1 (0.6–2.7)	1.0 (0.6–2.5)	0.9 (0.5–1.9)	0.8 (0.5–1.6)
IDN	59.6 (41.3–101.1)	47.4 (17.9–118.8)	40.8 (16.6–106.5)	31.0 (14.0–84.8)	24.8 (12.2–65.2)
IRL	2.3 (1.3–6.9)	0.8 (0.4–4.9)	0.7 (0.3–4.0)	0.5 (0.3–2.6)	0.5 (0.2–1.8)
IRN	5.7 (3.0–12.4)	3.5 (0.9–16.8)	2.9 (0.8–13.5)	2.1 (0.7–8.8)	1.6 (0.6–5.9)
IRQ	9.9 (5.9–22.0)	12.2 (2.5–90.8)	10.0 (2.2–74.9)	6.7 (1.8–49.9)	4.8 (1.5–32.8)
ISL	0.9 (0.5–3.0)	0.4 (0.1–2.3)	0.4 (0.1–1.9)	0.3 (0.1–1.2)	0.2 (0.1–0.8)
ISR	1.3 (0.8–2.5)	0.5 (0.2–2.1)	0.5 (0.2–1.6)	0.4 (0.2–1.1)	0.3 (0.2–0.8)
ITA	1.5 (0.9–3.6)	0.8 (0.3–4.1)	0.6 (0.2–3.2)	0.5 (0.2–2.1)	0.4 (0.2–1.4)
JAM	1.1 (0.7–3.2)	0.7 (0.2–3.6)	0.6 (0.2–2.8)	0.4 (0.2–1.8)	0.3 (0.1–1.2)
JOR	1.6 (0.8–5.2)	0.6 (0.2–3.5)	0.6 (0.2–2.8)	0.4 (0.2–1.8)	0.4 (0.2–1.3)
JPN	5.6 (3.1–12.5)	1.7 (0.8–8.8)	1.5 (0.7–7.1)	1.2 (0.6–4.8)	1.1 (0.6–3.4)
KAZ	20.7 (11.7–43.6)	17.5 (4.3–92.9)	14.7 (3.9–77.4)	10.6 (3.4–52.7)	8.0 (2.9–35.8)
KEN	32.0 (20.7–60.2)	40.7 (9.8–126.3)	33.4 (8.5–113.0)	22.8 (7.2–83.2)	16.3 (6.2–60.2)
KGZ	28.2 (18.3–50.1)	14.7 (5.5–57.0)	12.8 (5.1–47.6)	9.9 (4.6–33.5)	8.3 (4.2–24.1)
KHM	58.9 (45.3–84.3)	29.8 (15.3–75.4)	27.1 (14.5–67.1)	22.2 (12.9–53.6)	19.5 (12.0–42.2)
KNA	1.5 (0.7–3.3)	1.8 (0.4–11.2)	1.4 (0.4–8.9)	1.0 (0.3–5.6)	0.7 (0.2–3.6)
KOR	17.7 (12.7–30.1)	7.7 (2.7–42.1)	6.7 (2.5–34.5)	5.2 (2.3–23.3)	4.3 (2.2–16.2)
KWT	5.0 (3.2–8.7)	2.8 (0.9–10.9)	2.4 (0.8–8.9)	1.8 (0.7–6.0)	1.4 (0.6–4.2)
LAO	48.2 (34.0–71.1)	23.4 (9.9–65.8)	20.5 (9.2–56.0)	17.2 (8.6–40.9)	14.9 (8.0–32.7)
LBN	3.7 (2.0–7.6)	2.5 (0.6–15.0)	2.0 (0.5–11.9)	1.4 (0.4–7.6)	1.1 (0.4–5.0)
LBR	56.3 (34.0–108.2)	71.0 (20.9–136.2)	60.3 (17.9–129.4)	43.7 (13.5–112.5)	31.5 (10.9–99.8)
LBY	9.7 (6.3–16.3)	6.0 (2.1–32.1)	4.9 (1.9–25.9)	3.6 (1.5–16.8)	2.8 (1.3–11.1)
LCA	2.5 (1.4–7.3)	0.9 (0.4–3.3)	0.8 (0.3–2.6)	0.6 (0.3–1.7)	0.5 (0.3–1.2)
LKA	14.7 (8.6–28.6)	11.7 (3.3–62.2)	9.6 (3.0–50.2)	6.8 (2.4–33.6)	5.2 (2.1–22.8)
LTU	13.6 (7.9–22.8)	7.8 (2.6–31.1)	6.5 (2.5–25.1)	4.8 (2.1–16.5)	3.9 (2.0–11.3)
LUX	1.7 (1.0–3.7)	0.7 (0.3–3.4)	0.6 (0.2–2.8)	0.5 (0.2–1.8)	0.4 (0.2–1.3)
LVA	13.0 (7.8–21.4)	3.3 (1.7–10.0)	3.1 (1.6–8.4)	2.7 (1.5–6.4)	2.5 (1.4–5.2)
MAC	16.4 (10.2–34.2)	10.5 (3.2–59.2)	8.7 (3.0–48.9)	6.3 (2.4–33.4)	4.9 (2.2–22.9)
MAR	20.0 (12.9–33.5)	14.2 (4.5–68.4)	12.0 (4.2–56.2)	8.9 (3.5–38.3)	6.8 (3.0–25.9)
MDA	32.8 (19.5–67.1)	36.4 (9.4–123.1)	30.0 (8.4–108.9)	21.0 (6.5–81.4)	15.3 (5.5–58.6)
MDG	47.9 (31.5–73.4)	28.8 (9.5–89.0)	25.2 (9.1–78.3)	19.4 (8.1–58.6)	16.1 (7.3–44.0)
MEX	7.0 (3.6–14.6)	2.0 (0.9–11.5)	1.8 (0.9–9.3)	1.5 (0.8–6.2)	1.3 (0.7–4.3)
MKD	5.4 (2.7–11.2)	1.7 (0.8–7.1)	1.5 (0.7–5.7)	1.2 (0.6–3.9)	1.1 (0.6–2.9)
MLI	14.9 (10.4–25.9)	9.3 (3.2–41.5)	7.8 (2.9–33.8)	5.7 (2.6–22.5)	4.4 (2.3–15.3)
MLT	2.2 (1.3–6.3)	5.5 (1.4–70.3)	4.4 (1.1–56.8)	2.8 (0.8–36.2)	1.8 (0.6–22.6)
MMR	48.4 (33.6–68.2)	28.1 (10.5–76.8)	24.7 (9.6–65.8)	19.5 (8.5–47.8)	15.9 (7.9–35.2)
MNE	8.8 (4.7–18.0)	1.9 (1.0–4.0)	1.7 (1.0–3.5)	1.6 (0.9–2.8)	1.4 (0.8–2.6)
MNG	33.0 (23.4–50.5)	12.7 (5.4–49.7)	11.4 (5.2–41.3)	9.5 (4.8–28.8)	8.3 (4.4–21.0)
MOZ	56.9 (37.6–89.2)	44.7 (15.8–106.9)	38.2 (14.2–100.3)	29.5 (12.2–82.5)	23.1 (10.6–63.7)

	Status quo		2050 (95% UI) with additional reduction in ARI scenario		
ISO3 Code	2024 (95% UI)	2050 (95% UI)	1% reduction	3% reduction	5% reduction
MRT	28.7 (22.0–42.1)	9.3 (4.5–34.3)	8.5 (4.3–28.9)	7.5 (4.1–20.4)	6.7 (3.9–15.7)
MUS	6.3 (4.0–11.9)	3.9 (1.4–20.5)	3.3 (1.3–16.6)	2.4 (1.1–10.9)	1.8 (0.9–7.2)
MWI	41.5 (25.2–80.2)	44.8 (9.9–132.8)	37.6 (8.9–126.2)	26.8 (7.5–95.3)	19.9 (6.4–69.1)
MYS	17.8 (10.1–31.7)	23.0 (4.9–92.1)	19.1 (4.2–76.9)	12.8 (3.2–52.4)	8.9 (2.7–35.3)
NAM	68.0 (44.0–112.2)	63.4 (20.4–126.9)	54.9 (18.1–117.3)	40.5 (15.1–99.4)	30.9 (13.4–80.9)
NER	27.5 (19.3–38.0)	8.2 (3.8–24.7)	7.6 (3.7–20.7)	6.5 (3.4–15.1)	5.9 (3.4–12.1)
NGA	38.0 (25.7–68.9)	36.7 (13.2–111.3)	30.5 (11.7–95.1)	21.7 (9.1–67.7)	16.2 (7.6–49.2)
NIC	5.4 (3.2–11.0)	4.0 (1.1–30.1)	3.3 (0.9–24.0)	2.3 (0.7–15.2)	1.8 (0.7–9.7)
NLD	1.6 (0.9–5.9)	0.6 (0.3–3.6)	0.6 (0.2–2.9)	0.4 (0.2–1.9)	0.4 (0.2–1.3)
NOR	1.7 (0.8–3.7)	1.8 (0.3–15.1)	1.4 (0.3–11.9)	1.0 (0.2–7.4)	0.7 (0.2–4.6)
NPL	29.3 (18.4–47.6)	21.7 (5.8–85.0)	18.1 (5.3–71.4)	13.2 (4.6–49.7)	10.4 (4.1–34.6)
NZL	1.8 (1.0–3.7)	1.0 (0.3–6.3)	0.8 (0.3–5.0)	0.6 (0.2–3.2)	0.5 (0.2–2.1)
OMN	2.7 (1.5–7.0)	1.2 (0.5–6.7)	1.0 (0.4–5.4)	0.8 (0.4–3.4)	0.6 (0.3–2.3)
PAK	39.6 (26.8–61.8)	27.3 (10.2–77.7)	23.2 (9.2–65.3)	17.6 (7.7–45.8)	14.2 (6.8–33.1)
PAN	10.0 (5.9–18.6)	7.7 (2.2–40.9)	6.3 (2.0–33.6)	4.4 (1.5–22.5)	3.4 (1.4–15.2)
PER	25.8 (17.5–45.3)	12.0 (5.0–59.0)	10.3 (4.8–49.5)	8.1 (4.4–34.4)	6.7 (3.9–23.8)
POL	5.4 (2.9–11.8)	2.0 (0.8–8.9)	1.7 (0.7–7.1)	1.4 (0.7–4.8)	1.2 (0.6–3.4)
PRT	6.6 (3.5–13.5)	2.0 (0.9–8.9)	1.8 (0.8–7.2)	1.5 (0.8–4.7)	1.3 (0.7–3.3)
PRY	9.2 (5.7–20.8)	5.8 (1.8–41.2)	4.8 (1.6–33.3)	3.5 (1.3–21.7)	2.7 (1.2–14.3)
PSE	1.3 (0.8–3.2)	0.6 (0.2–4.5)	0.5 (0.2–3.6)	0.4 (0.2–2.4)	0.3 (0.1–1.6)
ROU	20.3 (13.6–30.4)	6.2 (3.1–17.4)	5.7 (2.9–14.8)	5.0 (2.7–11.1)	4.4 (2.5–8.9)
RUS	18.3 (11.7–36.5)	12.3 (3.5–70.3)	10.4 (3.3–57.9)	7.7 (2.8–39.1)	6.0 (2.4–26.7)
RWA	13.5 (8.3–22.3)	11.7 (3.3–42.9)	9.6 (3.0–34.8)	6.9 (2.5–22.7)	5.0 (2.1–14.8)
SAU	3.1 (1.7–6.6)	1.9 (0.6–17.1)	1.6 (0.5–13.5)	1.2 (0.4–8.5)	0.9 (0.4–5.5)
SDN	22.1 (14.8–36.5)	12.5 (4.3–55.1)	10.7 (4.0–45.3)	8.0 (3.6–31.2)	6.4 (3.4–22.3)
SEN	27.4 (19.0–48.5)	18.7 (6.5–73.9)	15.8 (5.9–62.1)	11.6 (5.0–43.6)	8.9 (4.5–30.9)
SGP	8.7 (4.8–14.8)	6.0 (1.6–33.0)	5.0 (1.4–26.5)	3.6 (1.3–17.1)	2.7 (1.1–11.3)
SLE	50.8 (34.1–90.1)	40.0 (13.0–131.3)	34.1 (11.8–119.5)	25.3 (10.0–93.3)	19.4 (9.0–70.4)
SLV	7.9 (4.4–14.1)	6.9 (1.7–48.3)	5.7 (1.4–38.7)	4.0 (1.1–24.8)	2.9 (1.0–16.6)
SOM	49.8 (33.5–91.4)	42.6 (14.3–126.9)	36.2 (12.7–118.5)	26.3 (10.2–93.7)	20.1 (8.4–69.2)
SRB	8.5 (4.6–17.8)	2.2 (1.1–12.0)	2.0 (1.0–9.7)	1.7 (0.9–6.9)	1.5 (0.8–5.0)
SSD	36.2 (21.6–79.3)	47.0 (8.4–149.2)	39.2 (7.3–139.6)	27.0 (5.5–105.5)	19.6 (5.0–75.5)
SUR	12.0 (7.3–21.4)	2.7 (1.5–8.1)	2.6 (1.5–6.9)	2.3 (1.4–4.9)	2.2 (1.3–4.1)
SVK	3.7 (1.7–10.5)	0.7 (0.3–1.6)	0.6 (0.3–1.4)	0.6 (0.3–1.3)	0.5 (0.3–1.1)
SVN	4.0 (1.8–11.7)	0.7 (0.4–2.6)	0.7 (0.4–2.3)	0.6 (0.3–1.7)	0.6 (0.3–1.4)
SWE	1.5 (0.8–3.5)	1.9 (0.4–14.5)	1.5 (0.3–11.5)	1.0 (0.2–7.2)	0.7 (0.2–4.6)
SWZ	67.6 (37.0–120.7)	89.6 (25.0–146.8)	77.7 (21.6–141.5)	58.1 (16.0–123.7)	42.3 (13.0–105.3)
SYC	4.8 (2.8–10.0)	3.0 (1.0–22.1)	2.5 (0.9–17.7)	1.8 (0.8–11.4)	1.4 (0.7–7.5)
SYR	5.1 (3.1–8.8)	1.3 (0.6–7.0)	1.2 (0.6–5.8)	1.0 (0.6–4.1)	0.9 (0.5–3.0)
TCD	26.2 (15.3–57.2)	24.6 (5.3–109.3)	20.4 (5.0–92.1)	14.5 (4.6–63.9)	10.8 (3.9–43.7)
TGO	11.1 (6.6–21.5)	16.3 (2.9–84.4)	13.2 (2.6–68.5)	8.8 (2.0–44.8)	6.0 (1.6–29.5)
THA	33.0 (22.0–48.8)	22.2 (8.5–68.1)	19.0 (7.6–57.8)	14.3 (6.1–40.4)	11.2 (5.5–28.2)
TJK	23.8 (15.8–38.7)	9.5 (4.1–31.4)	8.5 (3.9–26.6)	7.1 (3.8–19.7)	6.1 (3.4–14.3)
TKM	22.1 (15.3–34.1)	6.8 (3.2–23.9)	6.3 (3.1–20.0)	5.6 (3.1–14.5)	5.1 (3.0–11.1)
TTO	3.6 (2.1–7.1)	5.9 (1.1–35.1)	4.7 (0.9–27.8)	3.0 (0.7–17.3)	2.1 (0.5–10.8)
TUN	6.4 (3.6–12.8)	8.0 (1.6–48.5)	6.5 (1.4–38.8)	4.3 (1.1–25.2)	3.0 (0.9–16.5)
TUR	4.8 (2.6–10.8)	1.4 (0.7–5.2)	1.2 (0.6–4.3)	1.0 (0.6–3.0)	0.9 (0.5–2.2)
TZA	56.9 (35.2–98.2)	57.3 (16.1–123.6)	48.5 (14.1–117.8)	35.4 (11.9–101.0)	26.5 (10.1–80.8)

ISO3 Code	Status quo		2050 (95% UI) with additional reduction in ARI scenario		
	2024 (95% UI)	2050 (95% UI)	1% reduction	3% reduction	5% reduction
UGA	20.1 (13.3–32.8)	7.4 (3.5–27.7)	6.6 (3.2–22.9)	5.5 (3.0–16.0)	4.8 (2.7–11.7)
UKR	17.9 (9.7–33.9)	13.7 (3.8–62.4)	11.4 (3.5–51.1)	8.1 (2.9–34.4)	6.2 (2.6–23.9)
URY	5.1 (2.6–10.6)	5.1 (1.1–29.1)	4.2 (1.0–23.2)	2.8 (0.8–15.0)	2.0 (0.6–10.0)
USA	1.2 (0.6–5.9)	0.3 (0.2–1.2)	0.3 (0.1–0.9)	0.2 (0.1–0.7)	0.2 (0.1–0.5)
UZB	19.6 (11.8–37.2)	20.0 (4.9–79.1)	16.5 (4.4–65.7)	11.5 (3.7–45.0)	8.4 (3.0–30.0)
VCT	6.4 (3.7–11.7)	2.6 (1.1–15.4)	2.3 (1.0–12.4)	1.8 (0.9–8.1)	1.5 (0.8–5.5)
VEN	4.9 (2.9–10.0)	3.1 (0.8–18.4)	2.6 (0.8–14.8)	1.9 (0.7–9.7)	1.5 (0.6–6.6)
YEM	15.9 (9.9–26.8)	3.9 (1.9–12.0)	3.6 (1.8–10.0)	3.3 (1.7–7.6)	3.0 (1.6–6.1)
ZAF	67.9 (38.4–123.2)	88.5 (23.6–141.7)	76.0 (20.6–139.8)	55.9 (16.2–125.4)	40.9 (12.6–110.5)
ZMB	46.3 (31.2–73.0)	25.7 (9.8–97.3)	22.3 (8.9–84.2)	17.8 (7.8–63.1)	14.9 (7.3–47.3)
ZWE	32.5 (22.0–58.3)	22.8 (7.0–88.2)	19.3 (6.4–74.8)	14.1 (5.5–51.7)	11.1 (5.0–35.6)
SENSITIVITY ANALYSIS, ASSUMING INCREASED RISK OF RECENTLY ACQUIRED MTB IMMUNOREACTIVITY					
AFG	64.5 (38.0–122.0)	44.9 (12.2–166.3)	36.4 (10.5–138.6)	24.5 (8.5–93.5)	17.2 (7.2–61.9)
AGO	109.3 (53.4–215.8)	115.7 (26.8–295.9)	94.5 (22.8–269.0)	63.2 (16.9–215.5)	42.7 (12.5–156.9)
ALB	7.1 (3.6–16.5)	4.1 (1.0–35.6)	3.3 (0.9–27.8)	2.2 (0.6–16.9)	1.6 (0.5–10.3)
ARE	3.0 (1.1–10.8)	0.3 (0.2–0.9)	0.3 (0.1–0.9)	0.2 (0.1–0.8)	0.2 (0.1–0.8)
ARG	9.0 (5.4–17.2)	3.4 (1.0–22.1)	2.8 (0.9–17.2)	2.0 (0.8–10.6)	1.5 (0.7–6.8)
ARM	20.7 (9.1–59.3)	17.8 (3.2–112.8)	14.2 (2.7–89.4)	9.0 (2.0–55.5)	6.0 (1.6–34.2)
ATG	2.1 (1.0–5.9)	2.3 (0.4–24.7)	1.8 (0.3–19.1)	1.1 (0.3–11.5)	0.7 (0.2–6.9)
AUS	2.6 (1.3–7.0)	2.4 (0.4–29.6)	1.9 (0.4–23.0)	1.2 (0.3–13.9)	0.8 (0.2–8.4)
AUT	4.0 (2.1–9.5)	1.2 (0.4–5.7)	1.0 (0.4–4.4)	0.7 (0.3–2.8)	0.6 (0.3–1.8)
AZE	40.3 (30.5–54.6)	8.3 (5.3–12.9)	8.1 (5.0–12.6)	7.9 (4.7–12.3)	7.7 (4.6–12.0)
BDI	47.8 (28.2–94.0)	20.0 (6.8–105.4)	16.7 (6.0–85.6)	12.2 (5.0–56.8)	9.4 (4.4–38.0)
BEL	4.4 (2.4–8.3)	1.8 (0.6–10.6)	1.5 (0.5–8.2)	1.0 (0.4–5.0)	0.8 (0.3–3.1)
BEN	25.3 (14.6–43.7)	10.0 (3.6–47.3)	8.2 (3.0–37.4)	6.0 (2.4–23.7)	4.6 (2.0–15.5)
BFA	25.1 (13.0–52.3)	21.9 (4.3–129.7)	17.4 (3.6–103.0)	11.2 (2.7–64.1)	7.5 (2.2–39.5)
BGD	92.4 (55.2–183.3)	89.5 (23.0–259.0)	72.9 (19.6–236.2)	47.7 (14.8–185.0)	32.5 (11.2–132.9)
BGR	12.8 (7.1–27.6)	4.3 (1.5–36.3)	3.6 (1.4–28.4)	2.7 (1.1–17.6)	2.1 (1.0–10.9)
BHR	7.1 (4.0–12.9)	2.9 (0.8–15.4)	2.4 (0.7–12.0)	1.6 (0.6–7.4)	1.3 (0.5–4.7)
BHS	5.0 (2.6–10.3)	1.3 (0.5–7.1)	1.1 (0.5–5.5)	0.9 (0.4–3.4)	0.7 (0.4–2.1)
BIH	21.7 (12.4–48.3)	8.1 (2.7–53.1)	6.7 (2.5–42.3)	4.9 (2.1–27.0)	3.8 (1.9–17.7)
BLR	26.1 (13.6–66.8)	18.6 (3.8–149.8)	14.7 (3.4–120.1)	9.6 (2.5–76.0)	6.6 (2.2–47.6)
BLZ	12.3 (6.7–27.5)	6.8 (1.7–49.7)	5.4 (1.5–39.3)	3.6 (1.2–24.5)	2.5 (1.0–15.4)
BOL	43.9 (27.5–95.2)	23.2 (7.1–114.5)	19.1 (6.3–92.6)	13.4 (5.3–60.1)	9.7 (4.6–39.7)
BRA	16.0 (8.9–28.3)	7.5 (1.8–42.7)	6.1 (1.5–33.7)	4.2 (1.3–21.0)	3.1 (1.2–13.3)
BRB	0.3 (0.2–1.8)	0.1 (0.0–0.2)	0.0 (0.0–0.2)	0.0 (0.0–0.1)	0.0 (0.0–0.1)
BRN	23.8 (13.7–59.8)	18.7 (4.0–141.4)	14.9 (3.3–113.2)	9.7 (2.5–71.7)	6.5 (2.2–45.0)
BTN	44.6 (31.4–65.8)	10.9 (6.0–36.9)	10.2 (5.6–30.7)	9.0 (5.0–22.1)	8.3 (4.8–17.0)
BWA	69.5 (42.6–128.9)	34.4 (12.1–160.1)	28.4 (10.9–134.7)	20.5 (9.0–93.2)	15.3 (7.5–63.9)
CAF	70.1 (45.1–130.3)	27.0 (10.2–95.5)	23.3 (9.8–78.8)	18.4 (9.0–53.5)	15.2 (8.4–38.1)
CHE	3.1 (1.6–7.9)	1.0 (0.3–7.2)	0.8 (0.3–5.6)	0.6 (0.2–3.4)	0.4 (0.2–2.1)
CHL	6.9 (3.4–15.1)	2.2 (0.7–14.3)	1.8 (0.6–11.1)	1.4 (0.5–6.9)	1.1 (0.5–4.3)
CIV	52.1 (35.5–107.9)	22.7 (8.0–112.9)	19.0 (7.0–91.8)	13.6 (6.4–61.0)	10.7 (6.1–40.7)
CMR	75.3 (40.2–154.0)	53.5 (14.7–201.0)	43.4 (12.6–171.1)	28.7 (9.7–119.0)	20.4 (8.1–80.0)
COD	108.7 (58.3–214.7)	114.7 (23.4–277.2)	94.1 (19.8–261.5)	63.4 (15.1–184.6)	42.6 (12.1–135.6)
COG	123.7 (55.3–303.4)	155.6 (32.2–338.8)	128.7 (26.0–314.0)	86.6 (17.5–277.1)	57.9 (12.5–225.6)
COL	12.7 (7.7–24.1)	5.3 (1.6–35.5)	4.3 (1.4–27.8)	3.0 (1.2–17.1)	2.3 (1.1–10.7)
COM	18.9 (10.2–42.9)	13.5 (3.2–110.4)	10.8 (2.6–87.7)	7.0 (2.0–54.6)	4.9 (1.5–33.7)

	Status quo		2050 (95% UI) with additional reduction in ARI scenario		
ISO3 Code	2024 (95% UI)	2050 (95% UI)	1% reduction	3% reduction	5% reduction
CRI	5.8 (3.1–12.8)	1.6 (0.6–13.8)	1.3 (0.5–10.8)	1.0 (0.5–6.7)	0.8 (0.4–4.2)
CUB	5.2 (2.7–12.5)	1.6 (0.6–7.6)	1.3 (0.5–5.9)	1.0 (0.4–3.6)	0.8 (0.3–2.5)
CYP	2.5 (1.2–6.4)	3.1 (0.5–29.4)	2.4 (0.4–22.9)	1.5 (0.3–13.9)	0.9 (0.2–8.4)
CZE	3.5 (1.9–10.3)	0.6 (0.3–1.8)	0.6 (0.3–1.5)	0.5 (0.2–1.0)	0.4 (0.2–1.0)
DEU	3.3 (1.8–8.7)	0.8 (0.3–4.4)	0.7 (0.3–3.4)	0.5 (0.2–2.1)	0.4 (0.2–1.3)
DJI	155.2 (74.3–290.6)	191.4 (50.2–285.1)	166.7 (41.8–267.6)	117.7 (29.7–243.5)	82.1 (22.2–204.1)
DMA	5.6 (1.0–21.5)	0.6 (0.2–3.2)	0.6 (0.2–3.2)	0.5 (0.2–3.2)	0.5 (0.2–3.2)
DNK	3.3 (1.4–7.2)	1.7 (0.3–12.0)	1.4 (0.3–9.4)	0.9 (0.2–5.7)	0.6 (0.2–3.5)
DOM	23.7 (15.3–42.2)	7.3 (2.8–34.3)	6.2 (2.5–27.5)	4.9 (2.2–17.9)	4.0 (2.1–12.1)
DZA	25.9 (16.2–54.0)	16.0 (4.7–71.9)	12.9 (4.1–56.5)	8.5 (3.2–35.0)	6.0 (2.6–22.0)
ECU	23.5 (14.7–44.3)	8.9 (3.2–51.8)	7.5 (2.8–40.9)	5.4 (2.4–25.2)	4.2 (2.1–16.0)
EGY	9.6 (5.4–16.7)	4.2 (1.2–20.9)	3.4 (1.1–16.4)	2.4 (0.9–10.2)	1.8 (0.8–6.4)
ERI	35.5 (21.2–63.8)	17.0 (5.0–78.8)	14.0 (4.5–62.6)	9.8 (3.7–40.4)	7.3 (3.1–26.7)
ESP	5.5 (2.8–12.2)	2.2 (0.6–18.8)	1.8 (0.5–14.6)	1.3 (0.5–8.9)	1.0 (0.4–5.5)
EST	9.5 (5.5–16.6)	2.8 (1.0–12.8)	2.3 (1.0–10.1)	1.8 (0.9–6.5)	1.5 (0.8–4.3)
ETH	47.5 (31.1–79.9)	20.0 (7.3–81.5)	16.7 (6.5–66.2)	12.1 (5.5–43.6)	9.2 (4.8–29.2)
FIN	2.9 (1.6–7.2)	0.8 (0.3–5.0)	0.7 (0.3–3.9)	0.5 (0.2–2.4)	0.4 (0.2–1.6)
FJI	27.5 (14.6–54.0)	20.8 (4.1–102.8)	16.6 (3.7–82.1)	10.7 (3.0–52.0)	7.4 (2.5–33.1)
FRA	4.6 (2.5–10.2)	1.9 (0.6–11.0)	1.6 (0.5–8.6)	1.1 (0.4–5.3)	0.8 (0.4–3.4)
GAB	126.6 (62.3–251.7)	119.1 (27.9–298.5)	98.0 (23.7–268.5)	66.1 (17.8–230.4)	44.7 (14.5–173.1)
GBR	5.7 (2.6–11.9)	6.1 (1.1–34.5)	4.8 (0.9–26.8)	3.0 (0.6–16.2)	1.9 (0.4–9.9)
GEO	38.8 (25.1–63.2)	10.7 (4.5–37.2)	9.3 (4.2–30.1)	7.6 (3.8–20.3)	6.7 (3.6–14.4)
GHA	65.5 (38.3–159.2)	40.8 (10.6–217.7)	33.3 (9.5–187.4)	22.8 (7.9–124.7)	16.4 (7.0–82.5)
GIN	43.4 (27.4–90.6)	22.0 (6.8–145.2)	18.1 (6.2–117.1)	12.7 (5.2–75.1)	9.6 (4.4–48.2)
GMB	34.2 (21.0–65.9)	24.1 (8.3–106.9)	19.1 (6.9–84.7)	12.5 (4.9–52.9)	8.6 (3.8–33.4)
GNB	98.8 (49.0–203.4)	102.2 (20.4–289.4)	83.4 (17.4–260.6)	55.7 (13.4–219.7)	37.8 (10.2–163.0)
GRC	2.4 (1.3–5.6)	0.9 (0.3–11.4)	0.8 (0.2–8.8)	0.5 (0.2–5.3)	0.4 (0.2–3.2)
GRD	0.6 (0.3–1.2)	0.4 (0.1–2.7)	0.3 (0.1–2.1)	0.2 (0.1–1.2)	0.1 (0.0–0.8)
GTM	32.1 (18.7–65.1)	23.8 (6.2–132.8)	19.0 (5.1–106.9)	12.2 (3.8–68.3)	8.3 (3.1–43.3)
GUY	33.7 (18.3–69.5)	21.7 (5.0–119.2)	17.4 (4.4–94.1)	11.4 (3.5–59.6)	7.8 (3.0–38.5)
HKG	26.6 (16.4–55.6)	14.2 (3.7–89.0)	11.7 (3.2–70.9)	7.9 (2.8–45.1)	5.6 (2.3–29.1)
HND	18.9 (11.4–32.1)	4.4 (2.0–24.6)	3.9 (1.9–19.6)	3.1 (1.7–12.8)	2.7 (1.5–8.7)
HRV	8.1 (4.3–16.4)	1.5 (0.7–5.9)	1.4 (0.7–4.7)	1.2 (0.6–3.1)	1.1 (0.5–2.2)
HTI	58.4 (33.9–125.6)	37.5 (9.4–174.8)	30.7 (8.0–145.7)	21.0 (6.6–98.5)	15.0 (5.4–65.6)
HUN	7.1 (3.9–15.1)	1.4 (0.7–4.7)	1.3 (0.6–3.8)	1.1 (0.6–2.6)	0.9 (0.5–2.2)
IDN	111.5 (67.5–201.8)	91.7 (27.9–224.4)	75.6 (23.5–209.3)	51.4 (17.1–160.6)	35.6 (14.6–121.1)
IRL	3.7 (2.0–8.5)	1.2 (0.5–11.3)	1.0 (0.4–8.8)	0.7 (0.3–5.4)	0.6 (0.3–3.3)
IRN	10.1 (4.8–22.6)	6.8 (1.2–38.6)	5.4 (1.0–30.2)	3.5 (0.9–18.5)	2.4 (0.7–11.5)
IRQ	20.1 (10.0–54.6)	26.3 (4.1–222.7)	20.6 (3.4–181.9)	12.9 (2.4–117.9)	8.3 (1.8–74.5)
ISL	1.6 (0.9–3.8)	0.8 (0.2–5.6)	0.6 (0.2–4.3)	0.4 (0.1–2.6)	0.3 (0.1–1.6)
ISR	2.2 (1.3–4.2)	1.0 (0.2–4.7)	0.8 (0.2–3.6)	0.5 (0.2–2.2)	0.4 (0.2–1.4)
ITA	2.8 (1.6–5.2)	1.4 (0.4–9.7)	1.1 (0.3–7.5)	0.7 (0.2–4.5)	0.5 (0.2–2.8)
JAM	2.1 (1.1–4.5)	1.4 (0.3–8.3)	1.1 (0.3–6.4)	0.7 (0.2–3.8)	0.5 (0.2–2.3)
JOR	2.8 (1.3–6.1)	1.1 (0.3–8.1)	0.9 (0.3–6.3)	0.6 (0.2–3.8)	0.5 (0.2–2.4)
JPN	8.8 (4.9–16.5)	2.6 (1.0–18.9)	2.2 (0.9–14.9)	1.6 (0.7–9.2)	1.3 (0.6–5.9)
KAZ	41.9 (20.0–106.8)	35.0 (6.2–208.6)	27.8 (5.2–171.5)	17.8 (4.2–113.0)	12.3 (3.5–73.1)
KEN	67.8 (35.7–140.1)	89.2 (16.9–286.8)	70.7 (13.9–249.7)	45.2 (9.7–184.3)	29.1 (7.4–124.7)
KGZ	51.1 (27.8–113.3)	24.8 (7.2–120.8)	20.5 (6.3–99.0)	14.4 (5.2–64.8)	10.8 (4.6–42.6)

	Status quo		2050 (95% UI) with additional reduction in ARI scenario		
ISO3 Code	2024 (95% UI)	2050 (95% UI)	1% reduction	3% reduction	5% reduction
KHM	93.5 (63.5–161.8)	47.3 (19.3–146.5)	39.6 (17.4–124.3)	29.4 (14.8–88.5)	23.6 (13.3–64.4)
KNA	3.3 (1.4–8.1)	3.9 (0.7–27.3)	3.0 (0.5–21.2)	1.9 (0.4–12.8)	1.2 (0.3–7.7)
KOR	26.3 (16.5–56.6)	13.4 (3.5–92.6)	10.8 (3.1–73.8)	7.6 (2.6–46.7)	5.7 (2.3–29.7)
KWT	9.1 (4.8–17.1)	5.2 (1.3–23.6)	4.2 (1.1–18.4)	2.8 (0.8–11.5)	2.0 (0.7–7.3)
LAO	76.7 (47.1–137.3)	36.6 (12.0–130.0)	30.9 (10.8–111.2)	23.1 (9.7–77.8)	18.0 (8.7–52.3)
LBN	6.5 (3.5–13.4)	4.9 (0.8–35.1)	3.9 (0.6–27.4)	2.5 (0.6–16.7)	1.7 (0.5–10.2)
LBR	117.7 (61.2–257.8)	146.1 (39.7–292.8)	121.1 (32.1–274.1)	82.1 (21.5–228.3)	55.1 (15.4–204.2)
LBY	17.1 (10.1–34.0)	11.8 (3.4–72.3)	9.4 (2.8–57.2)	6.0 (2.1–35.7)	4.1 (1.7–22.5)
LCA	4.1 (2.2–8.1)	1.6 (0.5–7.2)	1.3 (0.4–5.6)	0.9 (0.3–3.5)	0.7 (0.3–2.2)
LKA	27.7 (14.2–70.7)	24.1 (5.3–155.7)	19.2 (4.4–123.8)	12.1 (3.3–77.0)	7.9 (2.6–47.4)
LTU	26.0 (13.1–49.8)	14.2 (3.7–71.8)	11.4 (3.1–56.7)	7.8 (2.5–35.1)	5.4 (2.2–21.8)
LUX	2.8 (1.5–6.2)	1.2 (0.3–7.5)	1.0 (0.3–5.8)	0.7 (0.2–3.6)	0.5 (0.2–2.3)
LVA	19.4 (11.9–33.1)	4.4 (2.0–20.3)	3.9 (1.9–16.0)	3.2 (1.6–10.2)	2.8 (1.5–7.0)
MAC	29.6 (17.1–82.4)	19.6 (4.2–132.7)	15.9 (3.6–107.4)	10.6 (3.1–68.9)	7.3 (2.5–43.7)
MAR	36.4 (20.4–76.3)	28.4 (6.1–162.9)	22.5 (5.4–132.1)	14.6 (4.3–84.3)	10.1 (3.7–53.1)
MDA	69.2 (34.5–162.0)	76.1 (14.9–272.4)	61.0 (12.5–237.5)	39.5 (9.1–174.4)	26.0 (7.1–119.1)
MDG	80.5 (43.9–149.9)	52.3 (12.7–187.9)	42.8 (11.3–156.0)	29.2 (9.1–108.2)	21.5 (8.4–75.2)
MEX	9.9 (5.8–18.9)	2.9 (1.2–25.3)	2.5 (1.1–19.8)	1.9 (0.9–12.2)	1.5 (0.8–7.7)
MKD	8.4 (4.5–14.9)	2.7 (0.9–15.2)	2.2 (0.8–11.9)	1.7 (0.7–7.4)	1.3 (0.6–4.7)
MLI	26.3 (16.6–56.8)	17.3 (4.3–93.6)	14.0 (3.8–74.5)	9.5 (3.1–46.8)	6.5 (2.6–29.5)
MLT	5.1 (2.6–16.0)	13.0 (2.8–179.6)	10.1 (2.2–144.3)	6.1 (1.4–91.1)	3.7 (0.9–56.0)
MMR	84.2 (49.3–140.5)	48.3 (14.6–156.5)	39.8 (12.7–131.3)	28.0 (10.2–90.3)	21.1 (8.8–60.9)
MNE	11.8 (6.7–20.5)	2.5 (1.2–7.3)	2.2 (1.1–5.9)	1.8 (1.0–4.0)	1.6 (0.9–2.9)
MNG	49.0 (30.9–91.9)	19.4 (6.0–107.8)	16.4 (5.7–87.2)	12.4 (5.3–56.5)	10.0 (5.0–36.8)
MOZ	106.2 (54.4–191.7)	84.6 (24.3–219.7)	68.8 (20.7–193.9)	47.0 (15.5–153.5)	33.0 (12.7–112.5)
MRT	42.3 (29.7–78.7)	13.6 (5.6–67.5)	11.6 (5.2–54.3)	9.1 (4.3–35.9)	7.7 (4.0–24.6)
MUS	11.4 (6.8–24.1)	7.6 (2.0–47.2)	6.0 (1.7–37.0)	4.0 (1.4–22.8)	2.7 (1.1–14.3)
MWI	83.8 (39.5–191.1)	94.2 (15.2–291.2)	75.8 (12.8–270.2)	49.6 (9.6–203.5)	32.8 (7.8–141.0)
MYS	36.7 (17.5–75.7)	49.9 (8.6–216.0)	39.5 (7.0–178.4)	24.7 (4.8–118.0)	15.6 (3.6–75.8)
NAM	136.7 (69.8–254.3)	120.0 (30.6–260.1)	99.5 (26.2–247.8)	68.9 (20.1–202.5)	48.8 (15.7–154.9)
NER	37.6 (26.1–62.8)	11.0 (4.7–50.6)	9.7 (4.3–40.5)	8.0 (3.7–26.1)	6.8 (3.4–17.6)
NGA	76.5 (45.1–155.0)	75.8 (22.5–241.3)	60.8 (18.3–204.2)	39.1 (12.7–138.8)	26.3 (10.0–95.3)
NIC	10.1 (5.4–21.8)	8.2 (1.6–74.6)	6.5 (1.3–58.6)	4.1 (1.0–35.8)	2.7 (0.8–21.7)
NLD	2.8 (1.5–6.7)	1.1 (0.3–8.3)	0.9 (0.3–6.4)	0.6 (0.2–3.9)	0.5 (0.2–2.4)
NOR	3.4 (1.4–9.0)	3.9 (0.5–37.9)	3.0 (0.4–29.5)	1.9 (0.3–17.8)	1.2 (0.2–10.7)
NPL	53.1 (27.1–107.1)	43.0 (8.3–194.3)	34.3 (7.0–158.2)	22.4 (5.5–104.1)	15.3 (4.8–67.7)
NZL	3.2 (1.7–6.9)	1.8 (0.4–15.1)	1.5 (0.4–11.7)	1.0 (0.3–7.1)	0.6 (0.2–4.3)
OMN	4.6 (2.5–9.9)	2.1 (0.6–15.8)	1.7 (0.5–12.3)	1.2 (0.4–7.4)	0.9 (0.4–4.6)
PAK	73.2 (43.3–130.6)	49.4 (15.5–170.6)	40.3 (13.2–140.0)	27.5 (9.9–92.4)	19.5 (8.0–60.5)
PAN	20.2 (10.2–42.8)	15.9 (3.0–96.4)	12.6 (2.6–76.0)	8.0 (2.1–46.8)	5.2 (1.6–28.9)
PER	41.6 (27.1–102.9)	20.8 (6.3–131.4)	16.9 (5.6–106.0)	11.8 (4.9–68.3)	8.8 (4.5–44.3)
POL	9.2 (4.7–16.8)	3.3 (1.1–19.8)	2.7 (0.9–15.5)	1.9 (0.8–9.5)	1.5 (0.7–6.0)
PRT	10.1 (5.4–18.1)	3.1 (1.1–19.7)	2.6 (1.0–15.4)	2.0 (0.8–9.5)	1.6 (0.8–6.0)
PRY	16.8 (8.7–42.8)	11.5 (2.4–97.5)	9.1 (2.1–77.1)	5.8 (1.7–47.9)	4.0 (1.4–29.6)
PSE	2.4 (1.1–5.6)	1.0 (0.2–10.4)	0.8 (0.2–8.0)	0.5 (0.2–4.8)	0.4 (0.2–2.9)
ROU	29.5 (19.8–50.7)	8.8 (3.5–32.7)	7.5 (3.3–26.4)	6.0 (3.0–17.8)	5.0 (2.7–12.6)
RUS	34.8 (19.1–81.6)	23.6 (4.8–152.8)	19.0 (4.2–126.1)	12.4 (3.4–83.2)	8.5 (3.0–53.0)
RWA	26.1 (14.0–51.4)	23.6 (5.0–99.8)	18.7 (4.1–79.3)	12.2 (3.1–49.7)	8.0 (2.6–31.1)

	Status quo		2050 (95% UI) with additional reduction in ARI scenario		
ISO3 Code	2024 (95% UI)	2050 (95% UI)	1% reduction	3% reduction	5% reduction
SAU	5.4 (2.9–12.5)	3.7 (0.7–41.6)	3.0 (0.7–32.3)	1.9 (0.5–19.5)	1.3 (0.5–11.8)
SDN	37.5 (21.9–80.5)	23.4 (5.5–124.9)	18.9 (4.9–100.6)	12.6 (4.2–64.3)	9.0 (3.7–41.0)
SEN	50.9 (29.4–106.7)	35.8 (8.5–164.3)	28.9 (7.6–134.2)	19.1 (6.3–87.7)	13.4 (5.1–57.5)
SGP	15.8 (8.6–31.9)	12.1 (2.2–76.0)	9.6 (1.9–60.2)	6.1 (1.5–37.5)	4.1 (1.3–23.2)
SLE	95.3 (51.7–209.3)	77.1 (17.9–262.6)	62.9 (15.6–228.5)	42.3 (12.5–198.3)	29.2 (10.4–141.2)
SLV	14.2 (7.6–32.4)	14.1 (2.4–120.1)	11.1 (2.1–95.1)	7.1 (1.6–58.9)	4.7 (1.2–36.0)
SOM	93.4 (52.1–214.2)	84.1 (22.7–250.0)	69.1 (19.0–241.5)	45.6 (14.1–191.8)	31.6 (10.9–141.3)
SRB	12.5 (7.3–23.1)	3.0 (1.2–26.0)	2.6 (1.1–20.4)	2.1 (1.0–12.6)	1.7 (0.9–8.0)
SSD	77.2 (31.5–190.0)	102.2 (12.7–345.4)	82.2 (10.8–309.0)	52.9 (8.1–234.5)	34.3 (6.2–160.8)
SUR	16.6 (10.7–28.4)	3.5 (1.7–15.0)	3.1 (1.6–12.1)	2.6 (1.5–8.1)	2.4 (1.4–5.7)
SVK	5.0 (2.3–11.4)	0.8 (0.4–2.8)	0.7 (0.4–2.2)	0.6 (0.3–1.6)	0.6 (0.3–1.3)
SVN	5.2 (2.7–12.3)	0.9 (0.4–5.2)	0.9 (0.4–4.1)	0.7 (0.4–2.7)	0.6 (0.3–1.9)
SWE	3.0 (1.5–8.3)	4.2 (0.6–35.0)	3.3 (0.5–27.3)	2.0 (0.3–16.5)	1.3 (0.3–9.9)
SWZ	147.1 (68.7–291.6)	189.3 (46.2–317.3)	161.2 (37.7–301.9)	110.8 (25.7–262.6)	75.2 (18.4–207.4)
SYC	8.7 (5.2–20.5)	5.8 (1.4–51.9)	4.6 (1.2–40.5)	3.0 (1.0–24.7)	2.0 (0.8–15.2)
SYR	7.6 (4.2–14.3)	2.0 (0.8–14.2)	1.7 (0.7–11.2)	1.3 (0.6–7.2)	1.1 (0.5–4.8)
TCD	52.9 (25.7–136.6)	49.9 (8.2–258.2)	39.8 (6.8–214.6)	26.0 (5.1–143.2)	17.0 (4.6–92.7)
TGO	23.4 (11.5–52.0)	36.7 (4.9–206.4)	28.7 (4.0–169.3)	17.6 (2.8–107.2)	11.1 (2.2–66.3)
THA	61.6 (35.4–105.0)	43.2 (12.1–146.1)	34.9 (10.3–120.9)	23.0 (8.2–81.2)	16.1 (6.6–54.0)
TJK	40.3 (24.4–79.9)	14.5 (5.5–61.7)	12.3 (4.8–49.5)	9.2 (3.9–32.4)	7.3 (3.8–22.4)
TKM	33.2 (21.4–64.4)	9.4 (3.8–46.9)	8.2 (3.5–37.6)	6.5 (3.1–24.6)	5.7 (3.1–16.8)
TTO	8.0 (3.7–17.2)	13.5 (1.9–87.0)	10.5 (1.6–68.6)	6.4 (1.1–42.2)	4.0 (0.8–25.7)
TUN	13.2 (6.2–31.5)	17.4 (2.8–122.7)	13.6 (2.3–97.0)	8.5 (1.6–59.7)	5.4 (1.2–36.3)
TUR	7.4 (3.9–14.1)	2.2 (0.8–11.1)	1.9 (0.8–8.7)	1.4 (0.7–5.5)	1.1 (0.6–3.6)
TZA	114.2 (61.3–217.9)	115.6 (27.0–264.8)	94.0 (22.4–247.0)	62.4 (16.0–206.3)	42.5 (12.3–162.1)
UGA	31.5 (20.7–62.1)	11.4 (4.4–58.4)	9.6 (4.0–46.4)	7.2 (3.3–29.6)	5.7 (3.0–19.4)
UKR	36.1 (17.7–80.3)	27.6 (5.9–146.4)	21.9 (4.8–118.5)	14.2 (3.7–75.4)	9.5 (3.0–47.5)
URY	9.8 (4.6–25.3)	10.9 (1.7–68.7)	8.6 (1.4–54.4)	5.4 (1.1–33.6)	3.5 (0.8–20.7)
USA	1.9 (0.9–6.1)	0.5 (0.2–2.5)	0.4 (0.2–1.9)	0.3 (0.1–1.2)	0.2 (0.1–0.8)
UZB	40.2 (20.2–88.3)	41.3 (7.6–181.3)	32.9 (6.4–151.2)	20.9 (4.8–99.6)	13.6 (3.8–63.1)
VCT	10.8 (6.2–22.9)	4.5 (1.4–35.2)	3.7 (1.3–27.6)	2.5 (1.0–16.9)	2.0 (0.9–10.5)
VEN	9.0 (4.6–20.4)	6.2 (1.1–40.9)	4.9 (1.0–32.1)	3.2 (0.8–19.8)	2.1 (0.7–12.3)
YEM	21.8 (14.7–35.0)	4.9 (2.3–24.2)	4.4 (2.1–19.2)	3.8 (1.8–12.4)	3.4 (1.7–8.3)
ZAF	144.2 (67.0–300.7)	185.9 (40.2–307.8)	157.6 (33.3–304.1)	108.9 (23.6–263.1)	74.0 (17.8–223.3)
ZMB	78.4 (43.0–150.5)	45.2 (12.0–205.9)	37.1 (10.9–176.2)	26.0 (9.5–122.8)	19.1 (8.2–85.0)
ZWE	59.8 (35.0–138.1)	42.7 (10.0–199.9)	34.8 (8.6–164.9)	23.5 (6.7–109.2)	16.7 (5.6–71.4)

Appendix Table 15. Relative difference (%) in risk of tuberculosis disease among those with Mtb immunoreactivity, compared to status quo (2024), under additional ARI reduction scenarios. Estimates are shown for 1%, 3%, and 5% additional annual ARI reductions by 2030 and 2050

	Additional 1% reduction in ARI scenario		Additional 3% reduction in ARI scenario		Additional 5% reduction in ARI scenario	
Country	2030 (95% UI)	2050 (95% UI)	2030 (95% UI)	2050 (95% UI)	2030 (95% UI)	2050 (95% UI)
Primary analysis						
China	−2.5 (−4.0 to −1.3)	−7.1 (−9.5 to −3.3)	−7.2 (−11.3 to −3.6)	−17.9 (−25.5 to −7.8)	−11.2 (−17.9 to −5.6)	−24.9 (−37.1 to −10.5)
India	−2.8 (−4.1 to −1.7)	−6.8 (−9.1 to −3.5)	−7.8 (−11.5 to −4.6)	−17.3 (−24.7 to −8.3)	−12.1 (−18.1 to −7.2)	−24.2 (−36.1 to −11.2)
Philippines	−2.9 (−3.8 to −1.6)	−7.0 (−9.1 to −3.8)	−8.3 (−10.9 to −4.5)	−18.7 (−24.3 to −9.2)	−13.0 (−17.2 to −7.0)	−27.0 (−35.2 to −12.5)
Viet Nam	−2.4 (−4.0 to −0.85)	−7.0 (−9.3 to −2.0)	−6.9 (−11.6 to −2.4)	−17.6 (−24.8 to −4.7)	−10.7 (−18.4 to −3.7)	−24.6 (−36.7 to −6.2)
Sensitivity analysis allowing for reversion of immunoreactivity						
China	−4.5 (−5.5 to −2.9)	−12.2 (−14.2 to −7.6)	−12.7 (−15.7 to −8.0)	−31.0 (−36.1 to −18.0)	−19.8 (−24.8 to −12.4)	−43.4 (−51.1 to −24.2)
India	−4.7 (−5.5 to −3.4)	−11.8 (−13.5 to −7.8)	−13.2 (−15.7 to −9.6)	−30.1 (−34.5 to −18.8)	−20.7 (−24.8 to −14.9)	−42.2 (−50.2 to −25.3)
Philippines	−4.7 (−5.4 to −3.4)	−11.5 (−13.5 to −7.9)	−13.5 (−15.3 to −9.4)	−30.1 (−34.4 to −20.2)	−21.1 (−24.2 to −14.6)	−43.6 (−50.2 to −27.6)
Viet Nam	−4.4 (−5.5 to −2.0)	−11.8 (−13.7 to −5.1)	−12.3 (−15.6 to −5.7)	−30.2 (−35.5 to −11.8)	−19.3 (−24.6 to −8.9)	−42.9 (−50.3 to −15.5)

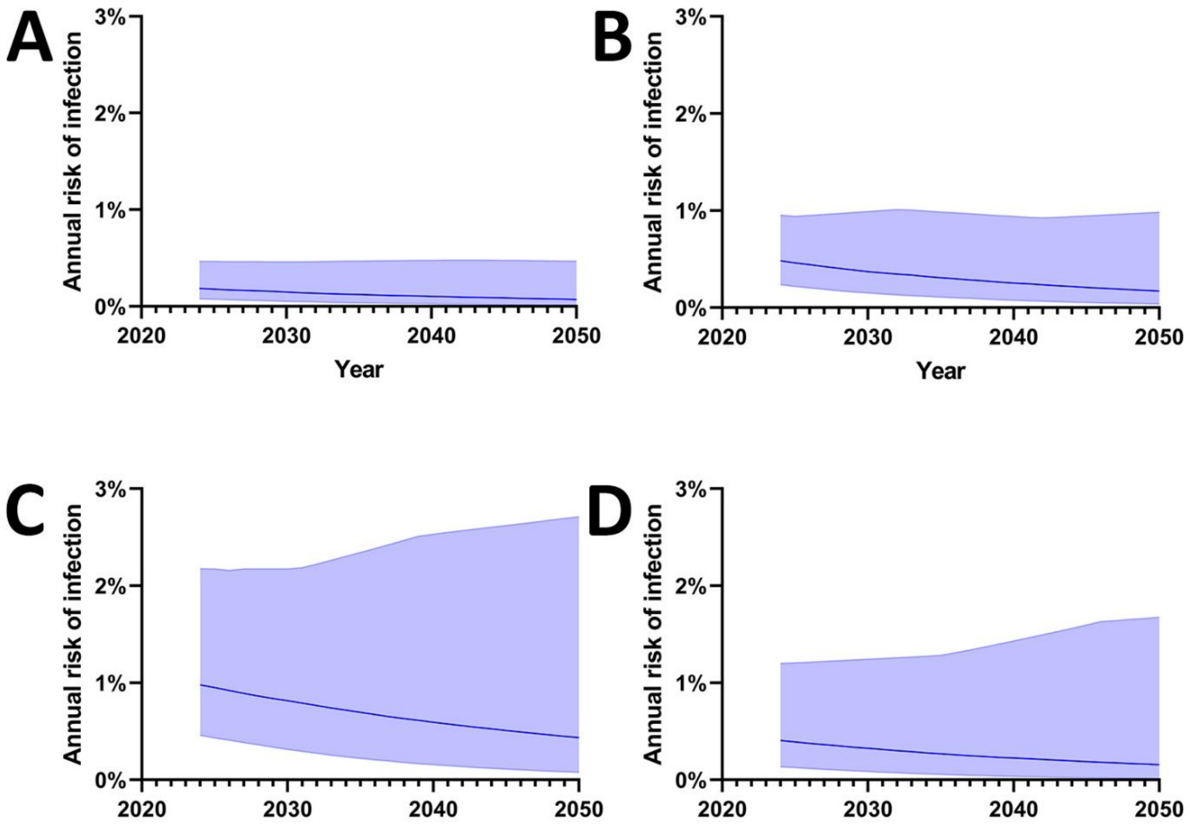

Appendix Figure 1. Projected annual risk of tuberculosis infection (ARI) trends under the status quo scenario from 2024 to 2050 for India, China, the Philippines, and Viet Nam. Note: Solid lines represent median estimates; shaded areas indicate 95% UI.

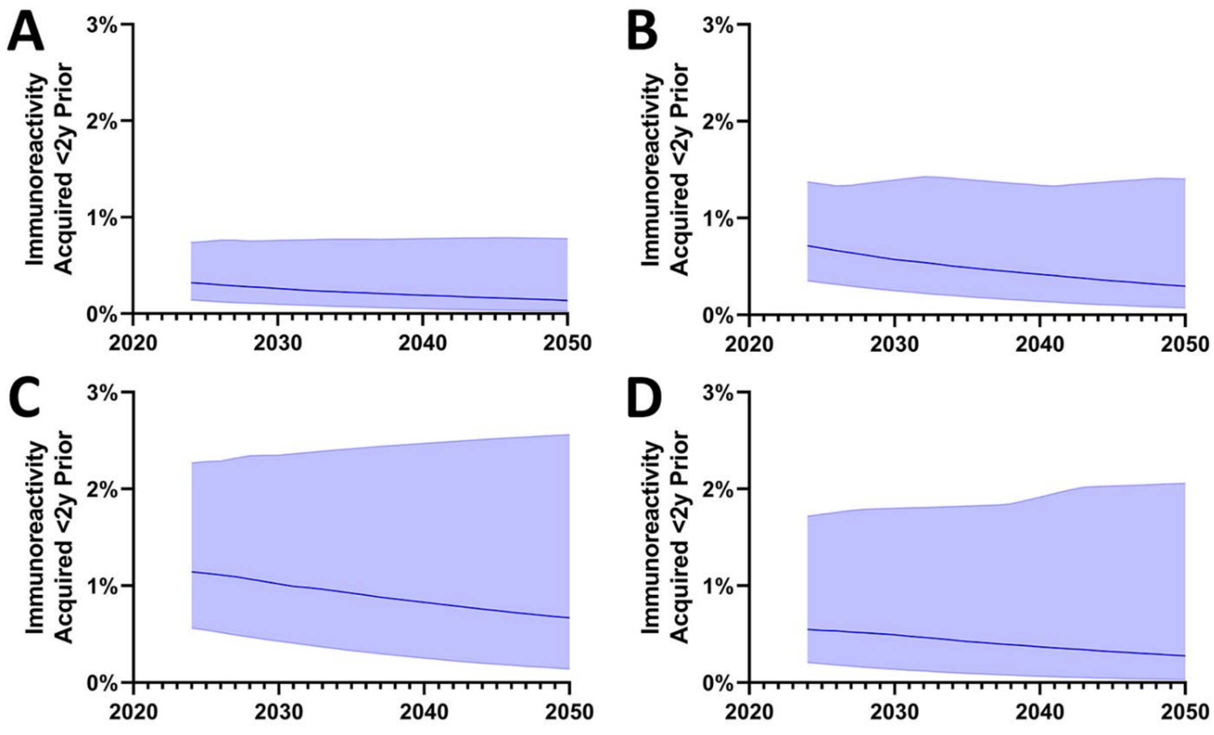

Appendix Figure 2. Projected prevalence of recently acquired Mtb immunoreactivity among immigrants from China, India, the Philippines, and Viet Nam under the status quo scenario, 2024–2050. Note: Solid lines represent median estimates; shaded areas indicate 95% uncertainty intervals (UI).

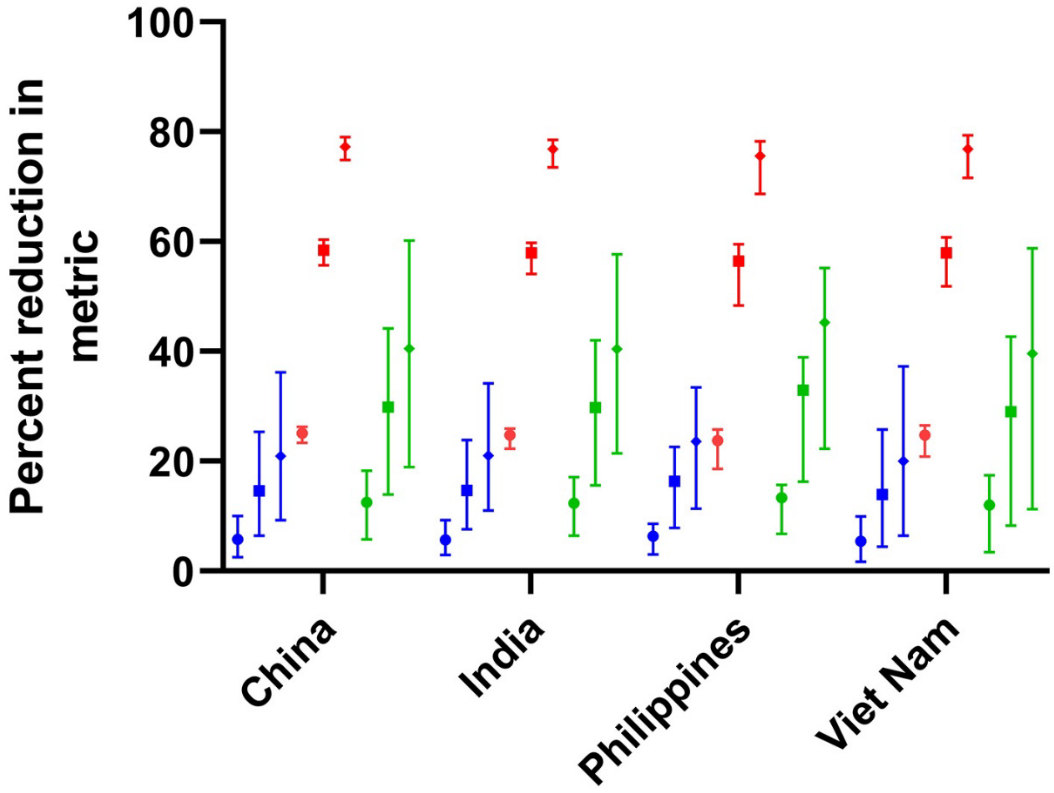

Appendix Figure 3. Relative reductions in tuberculosis indices by 2050 among immigrants from China, India, the Philippines, and Viet Nam under additional annual risk of infection (ARI) reduction scenarios, compared to the status quo (2024). Note: Points represent median relative reductions; error bars indicate 95% uncertainty intervals (UI). Blue represents overall tuberculosis immunoreactivity prevalence, red represents recent tuberculosis immunoreactivity prevalence, and green represents annual tuberculosis disease risk under additional ARI reduction scenarios: ● 1%, ■ 3%, and ♦ 5%.
